# Polymeric Smart Materials with Incorporated Small Molecules for Tissue Engineering: A Comprehensive Review

**DOI:** 10.3390/ijms27198704

**Published:** 2026-09-29

**Authors:** Polina Serbun, Roman Shaikenov, Petr Snetkov, Anna Kosova, Mikhail Budanov, Aleksandr Rybalka, Svetlana Morozkina

**Affiliations:** 1 Institute of Advanced Data Transfer Systems, ITMO University, Kronverkskiy Pr., 49, Bldg. A, 197198 Saint Petersburg, Russia; polinaserbunkl@gmail.com (P.S.); kosova@itmo.ru (A.K.); mikhailbudanov.2003@gmail.com (M.B.); al.v.rybalka@itmo.ru (A.R.); morozkina.svetlana@gmail.com (S.M.); 2 Progressive Materials and Additive Technologies Center, Kabardino-Balkarian State University Named After H.M. Berbekov, St. Chernyshevsky, 173, 360004 Nalchik, Russia; 3 Department of Translational Biomedicine, Saint Petersburg Research Institute of Phthisiopulmonology, Ligovsky Prospect, 2-4, 191036 Saint Petersburg, Russia; 4 Institute of Medicine, Saint Petersburg State University, Universitetskaya Emb., 7-9, 199034 Saint Petersburg, Russia; 5 International Research Centre for Nano Handling and Manufacturing of China, Changchun University of Science and Technology, Weixing Road, 7186, Changchun 130022, China

**Keywords:** polymeric smart materials, stimuli-responsive polymers, small molecule incorporation, tissue engineering, drug release, preclinical evaluation

## Abstract

This review presents an interdisciplinary analysis of small-molecule-encapsulated smart polymer materials for tissue engineering. Key design principles, including biocompatibility, degradation, and mechanical properties, are discussed, as well as the fabrication methods (hydrogels, electrospinning, porous matrices, and 3D printing). The following strategies for the immobilization of therapeutic agents are compared: physical retention, affinity binding, secondary carriers, post-loading, and covalent conjugation. Four main release mechanisms are analyzed, as well as material characterization, and the release kinetics are discussed. Preclinical in vitro and in vivo studies, including the disease models and the mechanistic validation, are presented, as well as a critical review of the clinical trials, identifying the following main reasons for failure: the lack of the superiority over the standard therapy, the complexity of the manufacturing, and the scale-up. The role of artificial intelligence in the acceleration of property prediction and system optimization is highlighted. Limitations are outlined and it is shown that successful translation requires a minimum level of sufficient complexity and a reproducible relationship between the structure, release, and the therapeutic window.

## 1. Introduction

Tissue engineering and regenerative medicine have evolved into multidisciplinary fields that integrate materials science, cell biology and clinical practice to restore damaged tissues and organs. The classical paradigm of using permanent implants or inert scaffolds has gradually given way to a more dynamic vision: biomaterials that not only provide temporary mechanical support but also actively participate in the healing process by delivering bioactive signals and responding to the changing microenvironment [1].

In this context, stimuli-responsive or “smart” polymers have attracted considerable attention. These materials undergo reversible changes in their physical or chemical properties upon exposure to external triggers such as temperature, pH, enzymes, light or magnetic fields. Such responsiveness enables on-demand drug release, dynamic modulation of scaffold mechanics and shape-morphing behavior, offering unprecedented opportunities for the creation of constructs that mimic the complexity of native tissues [2]. When combined with incorporated small bioactive molecules (including drugs, growth factors and signaling agents), these polymer matrices become multifunctional platforms capable of spatiotemporally controlled therapeutic delivery and the active guidance of cell behavior.

Despite these promising attributes, the translation of smart polymeric systems from laboratory concepts to clinical reality faces substantial obstacles. Scalability of fabrication, long-term stability of both thepolymer and incorporated cargo, batch-to-batch reproducibility and stringent regulatory requirements remain major barriers. Moreover, the inherent complexity of biological systems often means that in vitro performance does not reliably predict in vivo outcomes. These challenges necessitate a comprehensive, multidisciplinary approach that integrates advanced manufacturing techniques, thorough physicochemical characterization, release kinetics modeling and both preclinical and clinical evaluations.

Recently, artificial intelligence and machine learning have begun to reshape the biomaterials landscape. Predictive models can now estimate mechanical properties, degradation rates, and drug release profiles from chemical structure and processing parameters, dramatically accelerating the design–synthesis–testing cycle. These computational tools promise to reduce the development time and cost and enable personalized scaffold design tailored to individual patient needs.

The Russian scientific community has a strong tradition in macromolecular chemistry and biomaterials research, with numerous academic and research institutions actively contributing to the synthesis of biodegradable polymers, composite hydrogels, and stimuli-responsive systems for tissue regeneration. This thematic collection, “State-of-the-Art Macromolecules in Russia”, provides an ideal platform to showcase these national achievements alongside global progress.

### 1.1. Aim and Structure of This Review

This review presents a systematic, multidisciplinary overview of polymeric smart materials with incorporated small molecules for tissue engineering. We discuss the fundamental principles and physicochemical properties of these systems, survey the main methods of their preparation and examine the release profiles and stimulus-triggered mechanisms of action. We then review the preclinical investigations, covering both in vitro and in vivo models, as well as the current status of clinical trials. Finally, we critically analyze the scope and limitations of the developed approaches and materials with and without AI-driven approaches, identifying the key translational barriers and unmet clinical needs.

Unlike previously published reviews—which focused primarily on the general classification of stimulus-responsive polymers, drug delivery systems, clinical translation, or specific aspects of the application of smart biomaterials in tissue engineering—this review examines smart polymeric materials incorporating small molecules as a unified, multi-level system for tissue engineering. Thus, Zafaryab and Vig primarily focus on nanogels as platforms for the delivery of therapeutic agents in cancer therapy and wound healing [3], whereas Mohammadzadeh et al. provide a broader overview of stimuli-responsive biomaterials for the regeneration of cardiac, bone, and skin tissues [4]. Municoy et al. [5] and another review [6] classify stimuli-responsive polymers by stimulus type and mechanism, but do not systematically link small-molecule incorporation strategies to release kinetics or biological outcome. Subramanian et al. provide a detailed analysis of clinical translation and regulatory barriers, yet their focus remains on delivery systems rather than on the polymer–small molecule–tissue function triad [7]. Parmar et al. emphasize AI-driven design and predictive modeling, but do not address physicochemical compatibility between small molecules and polymer matrices or the resulting release behavior [8]. In contrast to these reviews, the present work integrates material design, molecular incorporation, release mechanisms, and biological response within a single analytical framework and extends this analysis from preclinical evidence to clinical studies and translational barriers.

### 1.2. Artificial Intelligence and Machine Learning in Smart Polymer Design

In recent years, the integration of artificial intelligence (AI) and machine learning (ML) has emerged as a transformative force in biomaterials science, offering powerful tools to address the inherent complexity of designing stimuli-responsive polymeric systems for tissue engineering. Biomaterials research has traditionally relied on a trial-and-error method, involving numerous experiments driven largely by experience, leading to substantial waste of resources, including manpower, time, materials, and finances [9].

This challenge has prompted research groups worldwide, including many Russian universities, to explore AI-driven approaches for accelerating the design and optimization of smart polymer systems. Below, we survey the main domains of AI application, highlighting both international advances and specific Russian contributions.

The application of AI to smart polymer development spans multiple complementary domains. First, ML models enable the prediction of the fundamental material properties, including mechanical strength, degradation kinetics and swelling behavior, directly from molecular structure and processing parameters, dramatically reducing the need for exhaustive experimental screening. Advanced deep-learning frameworks now model mechanical performance and mass-loss behavior from the molecular descriptors, thermal properties and spectroscopic signals, guiding the balance between degradability and toughness in biodegradable polymers [9].

In a representative Russian study, researchers at Tomsk Polytechnic University systematically compared Box–Behnken design, traditional machine learning algorithms and artificial neural networks for predicting fiber diameter and tensile strength of the electrospun polycaprolactone scaffolds. The neural network models accurately predicted the fiber diameter and provided reliable forecasts of the tensile strength, significantly outperforming conventional design-of-experiments approaches. This work demonstrates that the neural networks can be effective even on small experimental datasets with complex dependencies [10].

Second, ML has proven exceptionally valuable for the prediction and optimization of the drug release kinetics from the polymeric matrices, which is a critical capability for the design of smart materials with the incorporated small molecules. Traditional mathematical and empirical prediction methods are limited in their ability to capture the complex relationships among polymer composition, fabrication parameters and release behavior [11]. ML-based approaches, particularly artificial neural networks, have demonstrated significant promise in the estimation of release profiles across the diverse delivery systems, including matrix tablets, microspheres, hydrogels.

Third, AI is revolutionizing the design and characterization of scaffolds for tissue engineering. ML models have been developed to predict the inflammatory response of macrophages on nanofiber scaffolds, a critical parameter for the determination of the scaffold biocompatibility and regenerative potential. The Random Forest model achieved 92.8% accuracy in predicting inflammatory responses using the tumor necrosis factor-alpha as the output. Furthermore, deep-learning models, particularly convolutional neural networks, have demonstrated the potential to classify macrophage phenotypes directly from scanning electron microscopy images. The integration of graph theory with ML enables the quantitative analysis of scaffold connectivity patterns, revealing that electrospun membranes closely mimic the extracellular matrix architecture.

In parallel, Russian researchers are applying ML to the design and characterization of smart polymer systems. A collaborative team from Lomonosov Moscow State University and Skolkovo Institute of Science and Technology has developed IPECnet: the world’s first machine learning-based model for the prediction of the area of water solubility of the interpolyelectrolyte complexes (IPECs) for biomedical applications [12]. The model accounts for both physicochemical properties of polyelectrolytes and chemical structures of their monomeric units, enabling the rational design of smart polymer systems for bactericidal coatings as well as drug delivery in the future.

Fourth, AI is accelerating the optimization of the fabrication processes. Decision tree models have been developed to predict the bioink viscosity across the various compositions with high accuracy, significantly reducing the trial-and-error associated with the 3D bioprinting [13]. ML techniques have also been successfully applied to predict the scaffold strength, enabling data-driven optimization of the additive manufacturing parameters.

The international community has made substantial progress in the application of AI to smart polymer systems as well. These efforts span several interconnected directions.

First, the predictive modeling of drug release kinetics has advanced significantly. For example, a comprehensive review of ML approaches for the modeling of drug release demonstrated that ML uncovers the relationships between the formulation parameters and release behavior that the conventional models cannot capture [14]. The study further showed that the artificial neural networks can estimate the release profiles across diverse delivery systems, including hydrogels, microspheres and implants, capturing the complex nonlinear relationships between the polymer composition and release behavior.

Second, AI-assisted design of stimuli-responsive nanocarriers represents a major frontier. A comprehensive review highlights that the supervised ML algorithms can predict the drug-loading efficiency and release profiles of polymeric nanoparticles based solely on compositional characteristics [15]. This capability is directly relevant to smart polymer systems incorporating small molecules.

Third, high-throughput screening and formulation optimization have been accelerated by ML. Supervised learning methods trained on the formulation descriptors can reliably predict the long-acting drug release profiles, allowing inverse design of polymer-drug systems [16].

Taken together, these international efforts illustrate the breadth of AI applications in smart polymer research. When compared with the Russian contributions described above, several observations emerge.

Both Russian and international researchers have made substantial progress in the predictive modeling and optimization of polymer properties using AI. However, the application of AI specifically to the design of stimuli-responsive polymer systems and high-throughput autonomous experimentation remains an active area of development worldwide. Continued collaboration and data sharing between research groups, including those in the Russian Federation and abroad, will be essential for advancing the field.

Collectively, these AI-driven approaches enhance prediction accuracy, optimize material properties, accelerate development and enable innovations such as personalized medicine and responsive biomaterials. However, significant challenges remain, including data quality and availability, computational complexity, model interpretability and the need for interdisciplinary collaboration and regulatory frameworks to ensure safe and equitable AI integration in biomedical fields. The synergy among high-throughput experimentation, automated polymer synthesis and machine learning strategies is now enabling efficient data-driven design of polymer biomaterials, empowering both experts and non-experts to develop customized materials with tailored properties. As the field matures, AI is expected to become an indispensable partner in the rational design of next-generation smart polymeric materials for tissue engineering.

## 2. Basic Principles of Materials Design

Natural polymers and their composites with synthetic materials are capable of closely matching the characteristics of various tissues and promoting their regeneration, before subsequently biodegrading without the need for removal from the patient’s body. Polymeric biomaterials are increasingly used in tissue engineering due to their advantages and characteristics, such as an adaptable structure, exceptional flexibility, high biocompatibility, physiological activity and high mechanical strength. This has led to a significant body of research into polymeric materials in tissue engineering and, consequently, into the study of polymeric matrices as drug delivery systems. This field has subsequently developed into stimulus-responsive systems, in which drug release can be induced by external stimuli [17].

The use of such materials opens up a wide range of possibilities for the treatment of various pathological conditions. Such systems are being actively investigated for the regeneration of bone and cartilage tissue, the treatment of osteoarthritis [18] and bone defects [19], and the development of modern wound dressings for the management of acute and chronic wounds, including diabetic ulcers [20]. Furthermore, the polymer matrices incorporating small molecules are regarded as a promising platform for the regeneration of skin, soft tissues, nervous tissue and other organs [21,22].

The development of smart polymeric materials for tissue engineering is a multifactorial task that requires balancing biological, physicochemical, and engineering parameters. Unlike conventional biomaterials, modern polymeric systems are expected not only to provide the structural support for damaged tissues but also to actively participate in tissue regeneration by interacting with the biological microenvironment and controlling the release of incorporated therapeutic agents [23,24,25,26]. Therefore, the rational design of such materials should simultaneously consider biocompatibility, biodegradability, mechanical properties, scaffold architecture, loading strategy, and stimulus-responsive behavior [23,25].

Biocompatibility remains one of the primary requirements for polymeric materials intended for tissue engineering. Besides the absence of cytotoxicity, an ideal scaffold should support cell adhesion, proliferation, migration, and differentiation while minimizing undesirable immune reactions and chronic inflammation [24,25]. Rather than acting as biologically inert implants, modern polymeric matrices are increasingly designed to actively communicate with the surrounding tissues by mimicking the extracellular matrix (ECM) [25]. Such biomimetic behavior is achieved not only through the chemical composition of the material but also by reproducing its mechanical and topographical characteristics, including stiffness, porosity, surface morphology, and anisotropy, all of which regulate cellular behavior through the mechanotransduction pathways. Because no single material simultaneously possesses the optimal biological activity and mechanical performance, hybrid systems combining natural and synthetic polymers have become an attractive strategy to combine the advantages of both classes of biomaterials and minimize their limitations [25].

Controlled biodegradation represents another key principle in scaffold design. Ideally, the degradation rate of the polymer should closely match the rate of new tissue formation, allowing the implanted material to gradually transfer mechanical functions to the regenerating tissue. Excessively rapid degradation may lead to premature loss of structural integrity, whereas overly slow degradation can interfere with tissue remodeling and prolong the foreign body response [24]. Equally important, the degradation products should be non-toxic and safely metabolized or eliminated from the body [23,24]. For this reason, biodegradable aliphatic polyesters such as PLA, PGA, PLGA, and PCL are widely employed because their degradation kinetics can be precisely adjusted by varying the polymer composition and molecular weight [23]. Since regeneration rates differ substantially among tissues and depend on patient-specific factors such as age or pathology, tailoring the degradation profile is an essential aspect of personalized biomaterial design [24].

The structural and mechanical characteristics of polymeric scaffolds are also critical for successful tissue regeneration. Highly interconnected porous architectures facilitate cell infiltration, vascularization, and efficient transport of oxygen, nutrients, signaling molecules, and metabolic waste. Pore size and surface area directly influence the availability of cell-binding sites and therefore determine cell attachment and migration efficiency [24]. However, increasing porosity often compromises mechanical strength, making it necessary to optimize the scaffold architecture according to the target tissue [24,25]. Likewise, mechanical properties should resemble those of native tissues to provide adequate support throughout regeneration without causing mechanical mismatch [23,24,25]. Consequently, scaffold design requires balancing porosity, permeability, degradation behavior, and mechanical stability.

Another challenge is the use of polymeric scaffolds as carriers for biologically active small molecules. The physicochemical properties of the therapeutic compound, including molecular weight, hydrophobicity, electric charge, and chemical stability, largely determine the selection of the polymer matrix and the loading strategy [4,23]. Hydrophobic drugs are commonly incorporated into amphiphilic polymeric micelles possessing hydrophobic cores, whereas hydrophilic compounds are more effectively protected within hydrogels or polyelectrolyte complexes [23]. Furthermore, the biochemical characteristics of the target tissue should also be considered during the material design. For example, systems intended for tumor therapy are frequently engineered to remain stable under physiological conditions while releasing their payload in the acidic tumor microenvironment [4]. Such compatibility between the therapeutic molecules and polymer carriers improves loading efficiency, protects drugs from premature degradation, and increases local therapeutic concentration while reducing systemic side effects [4,23].

**Figure 1:**
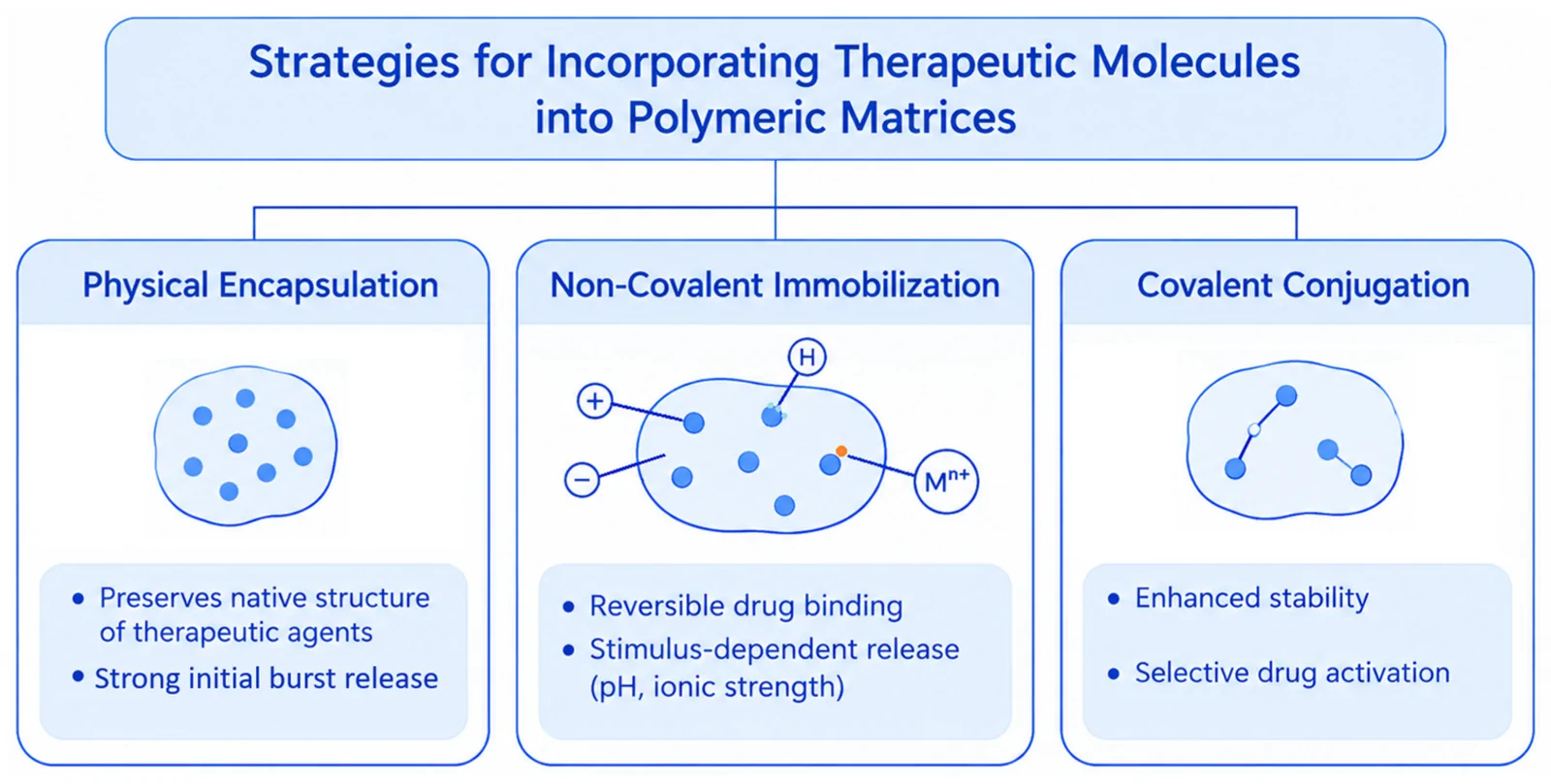
Three main approaches to immobilization of the drug molecule in the matrix. Each approach provides a different immobilization strength and, therefore, significantly affects the release kinetics.

The method used to incorporate therapeutic molecules into the matrices significantly affects the drug loading capacity and release kinetics. The following three principal approaches are generally employed: physical encapsulation, non-covalent immobilization, and covalent conjugation. Physical encapsulation preserves the native structure of sensitive therapeutic agents but is often associated with an undesirable burst release [23]. Non-covalent interactions, including electrostatic forces, hydrogen bonding, and coordination with metal ions, provide reversible drug binding and enable the stimulus-dependent release through changes in pH or ionic strength. In contrast, covalent conjugation through cleavable ester, acetal, hydrazone, or disulfide linkages produces polymer–drug conjugates with enhanced stability during circulation while allowing the selective drug activation under pathological conditions (Figure 1). Therefore, careful selection of the immobilization strategy has become an essential component of rational biomaterial design [4,23].

One of the defining characteristics of modern smart biomaterials is their ability to respond dynamically to external or endogenous stimuli [26]. Responsive polymers may be activated by physical, chemical, or biological signals [26,27]. Physical stimuli include temperature, light, magnetic fields, ultrasound, and electrical stimulation [4,27]. Thermoresponsive polymers such as PNIPAAm undergo a reversible phase transition near physiological temperature, making them suitable for injectable in situ gelling systems. Light-responsive systems enable precise spatial and temporal regulation of drug release, whereas electrically conductive and magnetically responsive polymers allow remote external control of therapeutic activity. Chemical responsiveness is primarily based on changes in pH, ionic strength, or redox potential, while biological responsiveness relies mainly on enzyme-sensitive polymers that selectively degrade in pathological tissues [4,27]. More recently, multiresponsive materials combining two or more mechanisms have captured the attention of researchers because they enable cascade-controlled drug release and more accurately reproduce the complexity of pathological microenvironments. Such multifunctional systems are considered one of the most promising directions in the development of adaptive biomaterials for tissue engineering and regenerative medicine [4,26].

Although the fundamental principles discussed above provide general guidelines for the development of smart polymeric materials, their practical implementation is rarely straightforward. The design of multifunctional biomaterials represents a typical multi-objective optimization problem in which the improvement of one characteristic frequently compromises another. Consequently, the goal of material design is not to maximize every individual property but rather to achieve an optimal balance between biological performance, structural stability, responsiveness, and clinical applicability [24,26,28]. As emphasized in recent reviews, the optimal combination of properties is highly application-specific, and no universal polymeric platform can simultaneously satisfy the requirements of all tissues or therapeutic practices [24,26,29].

One of the most significant trade-offs exists between biological activity and mechanical performance. Natural polymers, including collagen, gelatin, hyaluronic acid, chitosan, and alginate, possess excellent biocompatibility and closely resemble the extracellular matrix, thereby promoting cell adhesion, migration, and differentiation [23,24,25]. However, these materials generally exhibit poor mechanical strength, rapid degradation, and limited structural stability, restricting their use in load-bearing applications [24,28]. In contrast, synthetic polymers such as PLA, PLGA, PCL, and PEG provide superior mechanical properties, reproducible manufacturing, and tunable degradation kinetics but usually lack intrinsic biological recognition sites. Therefore, they require additional surface modification or incorporation of bioactive molecules to achieve comparable cellular responses [23,24,25,26]. Consequently, current scaffold design increasingly relies on hybrid systems that combine natural and synthetic polymers to exploit the advantages of both material classes while minimizing their individual limitations [24,25].

Another important compromise concerns the relationship between scaffold architecture and mechanical integrity. Highly porous structures facilitate cell infiltration, vascularization, nutrient diffusion, and metabolic waste transport, all of which are essential for successful tissue regeneration [23,24]. Increasing pore interconnectivity also improves the accessibility of incorporated therapeutic agents and enhances tissue ingrowth. Nevertheless, excessive porosity reduces stiffness and load-bearing capacity, potentially leading to premature structural collapse before sufficient tissue regeneration has occurred [24,28,29]. However, dense polymer networks provide greater mechanical support but limit cellular penetration and mass transport within the scaffold [24,28]. Therefore, the scaffold architecture should always be optimized according to the biomechanical and biological requirements of the target tissue [24,29].

A similar balance must be achieved between material functionality and translational feasibility. The incorporation of multiple responsive mechanisms, sequential drug release systems, or multifunctional therapeutic components substantially expands the capabilities of smart biomaterials and enables better adaptation to complex pathological microenvironments [4,26,27]. However, increasing material complexity also complicates synthesis, characterization, manufacturing, sterilization, quality control, and large-scale production. More sophisticated systems often face additional challenges related to batch-to-batch reproducibility, regulatory approval, long-term safety evaluation, and production cost, all of which may significantly limit clinical translation despite excellent laboratory performance. For this reason, recent studies increasingly emphasize that the successful clinical implementation of smart biomaterials depends not only on maximizing functionality but also on maintaining sufficient simplicity, reproducibility, and manufacturability [26,29].

Overall, the recent advances indicate a gradual shift in biomaterial design philosophy. Rather than pursuing universal multifunctional platforms with the highest possible number of responsive features, current research increasingly focuses on rationally balancing material properties according to the intended clinical application [24,28]. Such an application-oriented design strategy improves the likelihood of successful clinical translation [26,29].

## 3. Investigations of Materials’ Properties

### 3.1. Chemical Characterization

**Figure 2:**
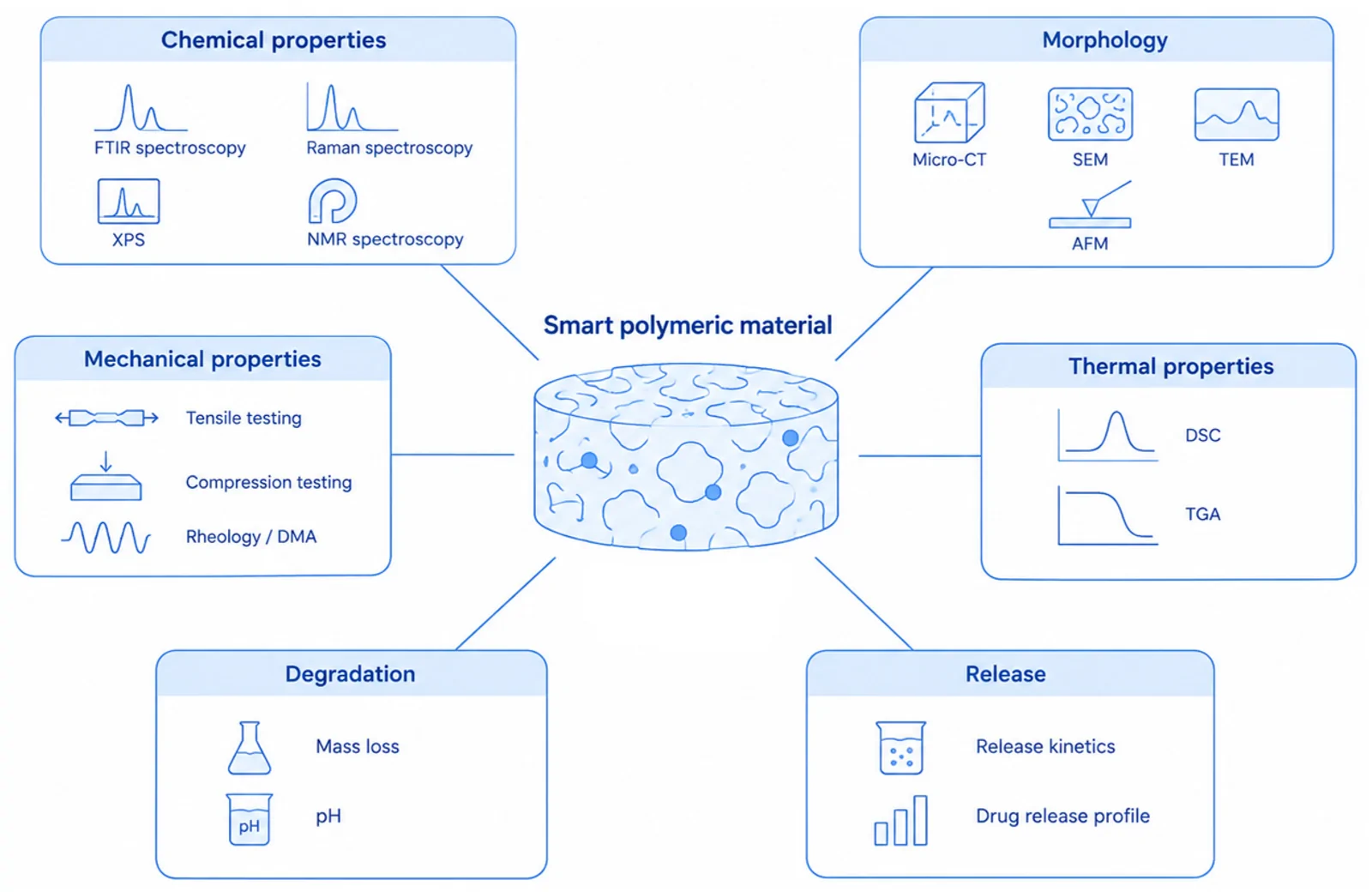
The main areas of characterization of polymeric biomedical materials, as well as the methods used and parameters studied in these areas.

A deep understanding of the chemical composition, molecular architecture, and supramolecular organization of polymeric materials is essential for their rational design in tissue engineering. Modern smart biomaterials require a detailed study of their chemical functionality, cross-linking chemistry, interactions between polymers and drugs, and molecular transformations caused by degradation [30,31]. The effectiveness of polymer scaffolds—ranging from their ability to support cell adhesion to their ability to control the release of therapeutic agents—is primarily determined by the chemical nature of the polymer chain, the spatial distribution of functional groups, and the dynamic evolution of the molecular structure under physiological conditions. Spectroscopic and analytical methods allow these chemical characteristics to be investigated at various scales [32,33]. Chemical characterization methods, as well as other methods that will be discussed in Section 3, are presented in Figure 2.

Among the most widely used methods for the characterization of polymers, Fourier-transform infrared spectroscopy (FTIR) plays a central role. This method is sensitive to molecular vibrations and capable of identifying specific functional groups, hydrogen bonds, and conformational ordering [30,33]. In the study of hydrogels, FTIR analysis allows for the determination of crosslinking mechanisms by identifying shifts in absorption peaks and the appearance or disappearance of bands associated with newly formed chemical bonds. For example, the crosslinking mechanism of hydrogels prepared from mixtures of polyacrylic acid and polyvinyl alcohol can be unambiguously identified as chemical crosslinking, primarily mediated by the formation of ester bonds, as evidenced by the appearance of characteristic carbonyl group absorption bands [30].

Beyond simple qualitative analysis, advanced FTIR techniques, such as FTIR spectroscopic imaging, have revolutionized the characterization of heterogeneous polymer systems, providing both chemical and spatial information simultaneously. Unlike traditional infrared spectroscopy, which provides an averaged signal across the entire sample, Fourier-transform infrared (FTIR) spectroscopy allows for the visualization of the distribution of functional groups, changes in crystallinity, chain orientation using polarized radiation, and the concentration of components in multiphase systems, including polymer blends, composites, and biodegradable matrices. As highlighted in the review, key areas of FTIR application include the investigation of the miscibility of polymer blends, real-time monitoring of polymerization and degradation processes, and identification of composition gradients or the migration of minor components—which is particularly valuable for the analysis of drug-eluting scaffolds, where low-molecular-weight additives may be heterogeneously distributed within the polymer matrix. When combined with chemometric algorithms, IR spectroscopy allows spectral profiles to be extracted even from minor components, making it an indispensable tool for both determining fundamental structure and ensuring quality control in the development of biomaterials [33].

In addition, diffuse reflectance infrared Fourier-transform (DRIFT) spectroscopy enables the analysis of solid polymeric materials that are difficult to study using conventional transmission modes as follows: powders, fibers, coatings, and heterogeneous composites. DRIFT spectroscopy is based on the interaction of infrared radiation with the sample surface and the collection of diffusely reflected light, providing detailed information about the molecular vibrations of functional groups located on the material’s surface. This method has proven particularly valuable for the characterization of biodegradable polymers, where surface chemistry critically influences protein adsorption and degradation behavior. For example, the DRIFT method has been successfully applied to monitor chemical changes in polymers exposed to environmental factors, thermal aging, or chemical modification, as well as to determine the degree of esterification in polysaccharide-based materials such as pectin and cellulose derivatives. Notably, DRIFT analysis of chitosan/β-1,3-glucan/hydroxyapatite biocomposites revealed characteristic spectral regions corresponding to the stretching vibrations of OH groups in hydrated water, as well as the stretching vibrations of CH and NH groups. These vibrational modes and carbonate/phosphate groups provide important information about the molecular composition of scaffolds for bone tissue engineering. Although DRIFT faces challenges related to quantitative analysis and dependence on sample homogeneity, its non-destructive nature and real-time analysis capabilities make it an invaluable tool for the chemical characterization of biopolymers and their composites [32].

Raman spectroscopy is another powerful method that provides information about molecular structure with minimal interference from water. This makes it particularly well-suited for hydrated polymer systems, such as hydrogels and biological matrices [31,34]. Raman scattering is an inelastic process in which the measured vibrational frequencies allow for the identification of chemical composition and the characterization of molecular structure. However, traditional Raman spectroscopy suffers from inherently weak signals, which have led to the development of advanced coherent Raman methods. Among these, coherent anti-Stokes Raman scattering (CARS) microscopy has become a revolutionary method of chemical imaging, capable of investigating local molecular composition, concentration, and even orientation with signal intensities orders of magnitude higher than those of spontaneous Raman scattering. This enables high-throughput imaging of large areas with excellent spectroscopic accuracy. CARS offers several unique advantages for polymer science: the blue-shifted anti-Stokes radiation does not interfere with fluorescence, and the high imaging speed allows for real-time monitoring of dynamic processes. In polymer research, CARS microscopy has been applied to a wide range of studies, including phase separation and crystallization in polymer blends, quantitative analysis of three-dimensional distribution of substances, visualization of molecular orientation in polymer fibers, as well as monitoring of drug release, water diffusion, self-recovery reactions, and the kinetics of photopolymerization. The spectroscopic precision of CARS allows for the differentiation between crystalline and amorphous polymer phases, monomeric and polymeric species, as well as pristine and oxidized polymer chains. Furthermore, polarization-controlled CARS enables the characterization of local chain orientation, providing insight into the heterogeneity in biopolymer fibers such as cellulose. Despite challenges related to non-resonant background contributions and the complexity of data interpretation, recent advances in data processing and machine learning have significantly improved the extraction of resonance spectra, similar to Raman spectra, from raw CARS data [31].

For the study of surface chemistry at the atomic level, X-ray photoelectron spectroscopy (XPS) is the leading method for the determination of the elemental composition and bonding configurations of the top 1–10 nm of polymer material surfaces. XPS is based on the photoelectric effect, in which electrons ejected from the material’s surface upon X-ray irradiation are analyzed to determine not only the elemental composition but also the chemical and electronic environment of the constituent atoms. This surface sensitivity is particularly important for biomaterials, where many functional properties—including cell adhesion, protein adsorption, biocompatibility, and degradation behavior—are determined by the surface characteristics. XPS allows for the qualitative and quantitative determination of elements such as carbon, oxygen, nitrogen, sulfur, silicon, and phosphorus, providing information on molecular components and functional groups. For example, XPS analysis of chitosan films reveals distinct C1s, O1s, and N1s spectra corresponding to the bond configurations such as C–C/C–H, C–OH, N–C=O, and O–C–O, allowing for detailed characterization of the surface modifications, functionalization, and contaminants. In tissue engineering applications, XPS plays an important role in confirming the successful surface modification of polymer scaffolds, assessing the surface composition of biocomposites, and quantifying protein adsorption. This method also allows for depth profiling using argon ion sputtering, revealing the distribution of elements across the material layers, although this approach can be challenging for soft polymeric materials due to structural damage. With detection limits ranging from 0.1 to 0.5 atomic percent and the ability to differentiate various biomolecules—proteins, polysaccharides, and lipids—based on characteristic functional groups, XPS remains an important tool for understanding the relationship between structure and properties [32]. A prime example of the practical application of RFES for monitoring the surface modification of biomedical composites is the work conducted by a team from several Russian research centers, in which polylactide- and hydroxyapatite-based composites (PLA/HA) were studied using the XPS method. XPS made it possible to demonstrate the selectivity of the modification process at the level of individual phases of the composite: hydroxyapatite proved to be practically inert to plasma exposure, whereas the main changes affected specifically the polymer phase—the atomic ratio [C]/[O] decreased, indicating surface oxidation, and new amide/carbonyl bonds (N–C=O/C=O) with a bond energy of 287.9–288.0 eV were formed. However, the authors specifically note a methodological limitation: due to the closeness of the bond energies, these two types of bonds cannot be unambiguously distinguished using XPS alone or by spectral deconvolution. The use of XPS to monitor the plasma modification of bioresorbable polymers is generally consistent with the widespread international practice of enhancing of biocompatibility of PLA scaffolds through plasma treatment [34].

Nuclear magnetic resonance (NMR) spectroscopy offers great potential to study the molecular structure, dynamics, and interactions of polymeric materials at the atomic level, making it indispensable for understanding of “polymer–drug interactions,” degradation mechanisms, and supramolecular organization in drug delivery systems. NMR methods are widely used to characterize biodegradable and bioabsorbable polymers, including polylactic acid (PLA), polyglycolic acid (PGA), polycaprolactone (PCL), and their copolymers. For amphiphilic block copolymers, such as PEG–PLA and PEG–PLGA, NMR studies in solution and in the solid state using ^1^H and ^13^C NMR have revealed the formation of solvent-dependent “core–shell” structures, where the hydrophobic polyester core securely encapsulates the therapeutic agents, while the hydrophilic PEG chains are oriented toward the aqueous medium, enhancing solubility and prolonging circulation in the bloodstream. Quantitative analysis using 1D ^1^H NMR revealed the orientation of PEG’s ethylene units depending on its molecular weight, which affects the surface functionality and circulation time. The molecular weight of the polyester blocks also affects the homogeneity of the nanoparticles: a lower-molecular-weight PLA (2 kDa) creates two distinct chemical environments observable in NMR, whereas a higher-molecular-weight PLA yields more homogeneous interphase structures. Diffusion-ordered NMR (DOSY) spectroscopy allows for the assessment of encapsulation efficiency. Furthermore, the integration of narrow and broad signals allows for the quantitative determination of free and bound polymer concentrations, which is important for the optimization of gene delivery systems, as demonstrated with polyethyleneimine (PEI)–DNA systems, where an excess of free polymer contributes to cellular toxicity. Delivery systems based on natural polysaccharides, including chitosan, dextran, alginate, and cellulose, present more complex challenges for characterization due to batch-to-batch variability and a broad molecular weight distribution. Nevertheless, NMR is widely used to study their structure, dynamics, and interactions in complex biological matrices, providing valuable insights into the carriers [35].

In addition to the basic spectroscopic methods described above, supplementary methods provide information necessary for a comprehensive characterization of polymers. Ultraviolet-visible (UV–Vis) spectroscopy enables the quantitative assessment of drug loading, encapsulation efficiency, and release kinetics of therapeutic agents incorporated into polymer matrices [35]. Mass spectrometry, especially when combined with chromatographic separation, allows for the identification of products and determination of degradation pathways, which is essential for ensuring the safety and biocompatibility of biodegradable implant materials [31]. Together, these methods provide a comprehensive analytical toolkit that enables researchers to correlate the chemical structure with the functional characteristics, optimize material design, and predict the in vivo behavior of the polymer systems for use in tissue engineering.

### 3.2. Morphological Characterization

The morphological characteristics of polymer scaffolds are critical factors determining their biological properties and their potential for cell adhesion, proliferation, and migration on the material [36,37]. Unlike the overall chemical composition, which primarily determines biocompatibility and degradation kinetics, the three-dimensional architecture and surface topography create a physical microenvironment in which cells perceive and respond to mechanical signals, ultimately guiding tissue formation [35,36]. Consequently, morphological characterization is a critical factor in the design of biomaterials [35,38].

Scanning electron microscopy (SEM) is among the most widely used methods for assessing morphology due to its exceptional resolution, large depth of field, and ability to reveal surface topography and internal architecture with nanometer precision [32,36,38]. SEM works by scanning a focused electron beam across the surface of a sample and detecting secondary or backscattered electrons to construct high-resolution images that capture fiber diameter, pore size, pore shape, and surface texture [32,36]. In the context of tissue engineering, SEM has proven invaluable for evaluating the microarchitecture of biomaterials and detecting defects. For example, SEM was used to characterize the electroformed nanofiber matrices made of gelatin for cardiac tissue engineering, which exhibited fiber diameters of 200–600 nm and mechanical properties similar to those of human myocardium. Similarly, SEM analysis of chitosan films containing selenium nanoparticles revealed successful incorporation of the nanoparticles and their influence on surface morphology, which correlated with enhanced proliferation of H9C2 cells. This method is also important for the evaluation of porous scaffolds produced by lyophilization, particle leaching, or 3D printing, as it allows the visualization of pore interconnections, pore wall thickness, and the distribution of the incorporated bioactive particles [36,38]. As highlighted in the reviews, SEM is particularly valuable for the assessment of the precision of scaffold fabrication—for example, to confirm the preservation of oriented porous structures after processing or to detect the presence of residual pore-forming agent particles [38]. However, SEM typically requires sample dehydration and a conductive coating (e.g., gold or platinum sputtering), which can lead to artifacts in fragile hydrogel structures [36]. Despite these limitations, SEM remains one of the most popular methods for the characterization of materials in tissue engineering.

Transmission electron microscopy (TEM) extends the capabilities of electron microscopy to the atomic and molecular scale, offering unprecedented resolution for studying the internal nanostructure of polymeric materials, the dispersion of nanofillers, and the ultrastructure of “polymer–drug” interfaces [25,36,38]. Unlike SEM, which provides information about the surface and near-surface layer, TEM requires ultrathin sections (typically 50–100 nm) through which the electron beam passes, allowing for the visualization of internal features such as crystalline domains, phase-separation regions, and the distribution of nanoparticles in the matrix. In tissue engineering applications, TEM is used to characterize the internal morphology of electroformed fibers, revealing the arrangement of polymer chains and the presence of “core–shell” structures in the coaxial electroformed scaffolds [25]. For example, during the development of the conductive CNT/GelMA hydrogel fibers for spinal cord regeneration, TEM images confirmed the uniform distribution of carbon nanotubes within the GelMA matrix. This nanoscale characteristic is crucial for understanding how the incorporated conductive materials influence the electrical and mechanical properties of the scaffold, as well as its ability to support neuronal regeneration and differentiation [37]. TEM also plays an important role in the evaluation of the encapsulation of therapeutic agents in polymer nanoparticles, allowing for the visualization of drug-loaded cores and the assessment of the particle size distribution. This method is particularly valuable for the characterization of self-assembled nanostructures, such as peptide amphiphilic nanofibers or polymer–peptide conjugates. However, TEM requires extensive sample preparation, including fixation, dehydration, embedding, and sectioning, which can lead to artifacts and is time-consuming [25]. Furthermore, the high-vacuum environment and exposure to the electron beam can damage sensitive polymer samples.

Atomic force microscopy (AFM) has become an indispensable tool for the nanoscale characterization of polymer biomaterials, offering a unique opportunity to simultaneously image surface topography, quantitatively assess mechanical properties, and map chemical interactions with a resolution of less than 1 nm [25,36,38]. Unlike electron microscopy techniques, AFM operates in air, liquid, or vacuum environments and does not require conductive coatings or complex sample preparation, making it particularly well-suited for studying hydrated hydrogels and biological samples under physiological conditions [36,38]. The fundamental principle of AFM involves raster-scanning the sample surface with a sharp probe and measuring the deflection of the cantilever to construct a three-dimensional topographic map. As highlighted in the reviews, the primary application of AFM in biopolymer research is the visualization of surface topography, which provides detailed information on surface roughness, porosity, and microstructural heterogeneity. For example, AFM imaging of chitosan and curdlan matrices in contact mode revealed significant structural differences between the two materials. An experiment with different polymer concentrations demonstrated how composition and concentration affect surface morphology. In addition to the topography, modern AFM instruments equipped with the PeakForce (PF QNM) quantitative nanomechanical mapping system allow for the simultaneous mapping of topographical and mechanical properties, including Young’s modulus, adhesion, and deformation. In the context of tissue engineering, AFM is widely used to characterize chitosan membranes, gelatin hydrogels, polycaprolactone composites, and cellulose-based materials, providing important information on how processing conditions and additives affect the surface roughness, mechanical properties, and, ultimately, cellular responses. Furthermore, force spectroscopy based on AFM allows for the quantitative assessment of molecular interactions, such as protein adsorption and cell adhesion forces to the matrix, which are fundamental for understanding the biocompatibility of biomaterials. However, AFM has limitations as follows: low scanning speed, limited imaging depth, and potential artifacts caused by the tip when studying soft samples. The combination of AFM with infrared spectroscopy (AFM-IR) and tip-enhanced Raman scattering (TERS) represents a state-of-the-art method for the chemical characterization of nanoscale samples, enabling simultaneous topographical and chemical imaging [25,38]. A clear example of the use of AFM for the quantitative assessment of the morphology of multicomponent scaffolds is the work conducted by the Biotechnology Center of the Russian Academy of Sciences in collaboration with Moscow State University, in which semi-contact AFM was used to study composites based on poly-3-hydroxybutyrate, chitosan, and hydroxyapatite, with the mineral filler contents ranging from 5 to 50 mass-%. It was shown that the dependence of the RMS roughness parameter on the hydroxyapatite content is non-monotonic: the value increased from 50 nm (pure PGB) to 154 nm upon the addition of 5% hydroxyapatite, then decreased to 77 nm at 20%, and at 50%, the surface was completely covered by aggregates of the mineral component. The authors note that AFM alone cannot explain the cause of this non-monotonic behavior; therefore, the data were supplemented with results from Fourier-transform infrared (FTIR) spectroscopy and differential scanning calorimetry (DSC). This allowed observation of the changes in the surface relief detected by AFM to be interpreted as the result of purely physical, rather than chemical, interactions between the components. This non-monotonic nature of the filler’s effect on the surface morphology is consistent with the data obtained for similar PGB/hydroxyapatite composites by international research groups [39].

While SEM, TEM, and AFM provide detailed information about the surface and near-surface layer, micro-computed tomography (micro-CT) offers non-destructive three-dimensional imaging of the matrix architecture, allowing for the quantitative assessment of the internal porous structure, interconnections, and spatial distribution of components throughout the entire volume of the matrix. Micro-CT works by acquiring a series of two-dimensional X-ray projections as the sample rotates, which are then computationally reconstructed into a three-dimensional image. This technique is particularly valuable for applications in tissue engineering, as it allows for the characterization of scaffolds in their natural, hydrated state without cutting or dehydration, thereby preserving their true three-dimensional architecture [25,38]. As noted in a review of oriented porous polymer scaffolds, micro-CT analysis played a crucial role in confirming the anisotropic porous structure of the scaffolds fabricated by supercritical fluid foaming, in which cells grow along the foaming direction—a feature that enhances cell proliferation and differentiation [38]. In cardiac tissue engineering, micro-CT was used to evaluate the effectiveness of 3D-printed bilayer scaffolds for cartilage and bone repair, demonstrating that a 3D-printed GelMA composite scaffold repaired the cartilage and bone damage in rabbits, bringing the tissue closer to normal [37]. Similarly, in the development of artificial tracheal scaffolds combining electroformed PCL nanofibers and 3D-printed PCL microfibers, micro-CT analysis showed that groups containing chondrocytes formed cartilage at the defect sites, confirming the efficacy of the matrix [36]. The quantitative capabilities of micro-CT extend to measuring porosity and pore size distribution—parameters that are essential for the prediction of cell infiltration, nutrient transport, and vascularization. However, micro-CT suffers from limited resolution compared to electron microscopy (typically 1–50 μm), and distinguishing between the polymer and soft tissues can be challenging without contrast agents. Nevertheless, combining micro-CT with histological methods allows for the observation of tissue proliferation and scaffold degradation over time [25,38].

Roughness at the micro- and nanoscales is a critically important morphological parameter that directly influences cell–material interactions, modulating protein adsorption, cell adhesion, proliferation, and differentiation through mechanotransduction pathways. Cells respond to topographical signals by altering the cytoskeleton and activating signaling cascades that ultimately regulate gene expression and cell fate [25,38]. As mentioned earlier, AFM is often the method of choice for studying roughness. For example, AFM analysis of chitosan films containing free and nanoencapsulated copaiba essential oil revealed significant changes in the surface roughness parameters, with the inclusion of dye molecules leading to the formation of larger defects. Similarly, the effect of crosslinking conditions on chitosan membranes was investigated, revealing a significant change in the crosslinking density and surface topology depending on pH [38]. Additionally, increased surface roughness can promote the adsorption of fibronectin and vitronectin, enhancing cell adhesion. However, excessive roughness can be detrimental, causing stress concentration and reducing cell proliferation [25]. Therefore, the optimization of surface roughness requires careful consideration of the target cell type and the intended application of the tissue.

Overall, the morphological characterization of polymer scaffolds for tissue engineering requires a multi-component approach, covering scales ranging from the nanometer-scale surface features to millimeter-scale scaffold architecture. The use of these methods, combined with the quantitative analysis of pore size, porosity, and surface roughness, provides a comprehensive understanding of the matrix morphology, which is essential for the optimization of the material’s design.

### 3.3. Mechanical Properties

**Figure 3:**
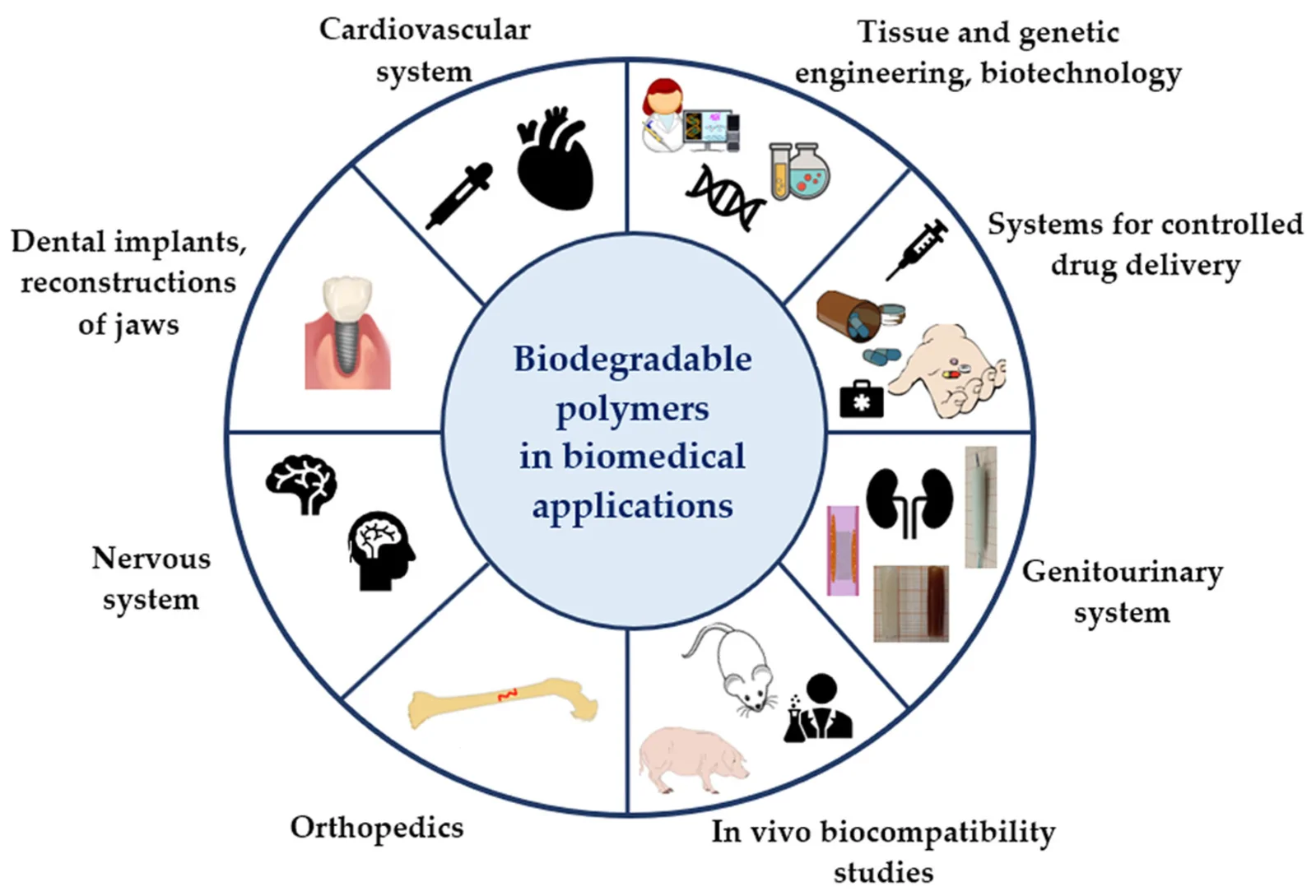
The mechanical properties of the polymeric materials must be appropriate for the purposes for which they are created and also be suitable for implantation of the material into the corresponding tissues [43]. Distributed under the terms and conditions of the Creative Commons Attribution (CC BY) license.

The mechanical properties of polymer biomaterials are fundamental factors determining their clinical efficacy, as they determine the ability of scaffolds to provide structural support, withstand physiological loads, and maintain functional integrity throughout the tissue regeneration process [40]. Mechanical characterization examines the dynamic interaction between the material and the surrounding tissue, encompassing everything from the elastic response to small deformations to viscoelastic and fatigue behavior under cyclic physiological loading [40,41]. Indeed, the mechanical mismatch between the implanted scaffold and the surrounding native tissue remains one of the primary causes of implant failure, leading to premature rupture of vascular grafts or insufficient load transfer in tendon reconstruction [41,42]. Examples of various uses of polymers with different mechanical properties are shown in Figure 3. Consequently, comprehensive mechanical characterization—encompassing tensile strength, compressive modulus, Young’s modulus, viscoelasticity, rheology, and fatigue behavior—is a critical factor in tissue engineering.

Tensile strength is the maximum stress a material can withstand before failure under uniaxial tension. This is one of the most fundamental mechanical parameters for polymer scaffolds, especially those intended for load-bearing applications such as bone fixation, tendon repair, and vascular grafts [40,42,43]. The tensile properties of polymer biomaterials are determined by their molecular architecture, crystallinity, molecular weight, and the presence of plasticizers or reinforcing fillers [42,43]. Mechanical characterization of the commercially available biopolymers reveals significant variability in tensile strength depending on polymer composition and the ratio of components in the blend. For example, the uniaxial tensile testing of Resomer^®^ LR 704 S (PLDLLA), manufactured by compression molding, demonstrated linear-elastic behavior with brittle fracture, showing an average tensile strength at break of 52.25 MPa and an elongation at break of approximately 5.8%, reflecting the stiffness and brittleness inherent in polyesters with a high lactide content. In contrast, Resomer^®^ LC 703 S (PLCL containing 30% PCL) exhibited a completely different mechanical response: an initial linear elastic region was followed by yielding and subsequent hardening under deformation, reaching a tensile strength of 19.65 MPa and an impressive elongation at break of 703%—a difference in ductility of more than two orders of magnitude. This sharp shift from brittle to ductile behavior highlights the profound influence of copolymer composition on mechanical properties, with the incorporation of flexible PCL segments imparting elastomeric characteristics to the PLA matrix [32,34]. Blending PLDLLA and PLCL in various ratios allows the mechanical properties to be tailored to the specific application requirements [40]. The mechanical properties of PLA-PCL blends depend heavily on the molecular weight and degree of crystallinity; PCL is not typically used alone in applications requiring structural integrity due to its ductile nature, whereas its combination with PLA combines the tensile strength of PLA with the elastic properties of PCL [42].

Young’s modulus (elastic modulus) quantitatively determines a material’s stiffness within its elastic deformation range. It represents the ratio of stress to strain under uniaxial tension or compression [40,41,43]. This parameter is of paramount importance in tissue engineering, as the elastic modulus of the scaffold should ideally match that of native tissue to ensure proper load transfer and cellular mechanics [40,43].

Research on two-photon polymerization (TPP) demonstrates that the Young’s modulus of printed structures can be adjusted within a range of 0.3 GPa to 1.43 GPa by varying the content of the crosslinking agent PEG600DMA. This mechanical tunability is particularly important for biomedical applications, enabling the fabrication of microscale devices with stiffness tailored to specific tissues. The study also demonstrated that the incorporation of MXene conductive nanomaterials into the photoresist composition did not compromise the mechanical integrity, with the Young’s modulus remaining at 0.8 GPa—a value comparable to that of the base composition [41].

The compressive modulus is a measure of a material’s stiffness under compressive loading. It is particularly important for the scaffolds intended for use in bone tissue, cartilage, and intervertebral disks, where compressive forces predominate. In contrast to the tensile properties, which are primarily determined by the polymer chain alignment and crystallinity, the compressive properties depend heavily on the scaffold’s porosity, pore architecture, and degree of crosslinking. The mechanical properties required for load-bearing applications vary significantly depending on the tissue type: bone tissue requires compressive moduli in the GPa range, whereas for soft tissues such as skin and cartilage, the moduli range from kPa to MPa. Therefore, when designing scaffolds for bone tissue engineering, it is necessary to strike a delicate balance between sufficient compressive strength to withstand physiological loads and sufficient porosity to facilitate cell infiltration and nutrient transport [43].

Ceramic materials and polymers each have their own advantages and limitations. Ceramics possess good osteoconductive and bone-binding properties but are brittle, whereas polymers offer greater design flexibility but often lack the compressive strength required for load-bearing applications [43]. The development of composite materials that combine biodegradable polymers with ceramic reinforcing elements, such as hydroxyapatite, represents a promising strategy for increasing the compressive modulus while maintaining biocompatibility and biodegradability. The use of polyesters, such as PLA, PLGA, and PCL, in bone tissue engineering has been extensively studied, with the mechanical properties adjusted through copolymerization and blending to achieve the desired balance of strength, ductility, and degradation kinetics [42,43].

Viscoelasticity is a time-dependent mechanical behavior that combines viscous and elastic characteristics. It is a fundamental property of polymeric biomaterials that significantly influences their performance under physiological loading conditions [40,42]. Biopolymers such as PLA and PCL exhibit viscoelastic behavior; that is, their mechanical response depends on temperature and the rate at which a load or strain is applied. This sensitivity to strain rate is characteristic of thermoplastic polymers and must be carefully considered when designing medical devices subjected to dynamic physiological loads, such as vascular stents, tendon repair devices, and orthopedic fixation systems [40,42]. Constitutive modeling of the viscoelastic behavior using state-of-the-art models has demonstrated superior accuracy in describing the complex mechanical response of biopolymers under various loading conditions [40].

Rheology provides critical insights into the processability and handling characteristics of polymer biomaterials, which aids in the selection of manufacturing methods and the prediction of in vivo performance [40,42]. The rheological behavior of polymer solutions and melts is determined by molecular weight, molecular weight distribution, chain entanglement, and the presence of additives or fillers [42]. In the context of tissue engineering, rheological characteristics are crucial for the optimization of processing methods such as electrospinning, 3D printing, and injection molding, where the flow properties of the polymer determine the feasibility and quality of the final scaffold [40,42]. The melt flow index and viscosity of polyesters, such as PLA and PLGA, are critical processing parameters; higher molecular weight generally leads to increased melt viscosity and improved mechanical properties, but reduced processability [42].

Furthermore, research on two-photon polymerization illustrates the importance of rheology in advanced manufacturing, where the viscosity and photopolymerization kinetics of the photoresist composition must be carefully controlled to achieve high-resolution printing. The addition of PEG600DMA to the photoresist composition improved the printability and increased the flexibility of the printed structures, demonstrating how rheological modification can expand the capabilities of additive manufacturing for biomedical applications [41].

Fatigue behavior is a material’s response to cyclic loading. It is of paramount importance for the polymer scaffolds intended for applications subject to repetitive mechanical loads, such as cardiovascular stents, orthopedic fixation devices, and tendon repair implants [40,43]. Unlike a monotonic load, which provides a single measure of strength, fatigue testing evaluates a material’s durability under repeated load cycles by determining of the stress level at which failure occurs after a specified number of cycles [40,43]. Implantable devices are subjected to various loading conditions in vivo, including shear, creep, and cyclic loading, requiring a comprehensive understanding of fatigue behavior for reliable device design [40].

The fatigue properties of biodegradable polymers depend on the molecular weight, crystallinity, and degradation kinetics, with lower molecular weight and higher crystallinity generally leading to reduced fatigue resistance [43]. The degradation of polymers over time further complicates the fatigue behavior, as progressive loss of molecular weight and the formation of microcracks can accelerate fatigue failure [42,43].

A comprehensive mechanical characterization of polymer scaffolds—including the tensile strength, Young’s modulus, compressive modulus, viscoelasticity, rheology, and fatigue behavior—enables the rational design of biomaterials with properties tailored to specific tissue engineering applications. An ideal scaffold should degrade at a rate that matches the formation of new tissue, gradually transferring the mechanical function from the implant to the regenerating tissue. The issue of material degradation will be discussed in more detail below.

### 3.4. Thermal Properties

Thermal properties determine not only the processing conditions and structural stability, but also the functional behavior of thermosensitive systems. Consequently, characterization of thermal transitions provides important information for understanding the relationship between polymer composition, molecular architecture, and biological characteristics [44,45,46].

Differential scanning calorimetry (DSC) is one of the primary methods used to study thermal transitions in smart polymer materials. The method measures the heat flux associated with the phase transitions and provides the information on the glass transition temperature (Tg), melting temperature (Tm), crystallization behavior, and other thermodynamic processes occurring within the polymer network [44,45]. For thermosensitive polymers, DSC is particularly valuable for assessing how polymer composition, molecular weight, crosslinking density, or the incorporation of biologically active small molecules affect molecular mobility and phase behavior. Changes in thermal transition temperatures often indicate modifications in intermolecular interactions, crystallinity, or flexibility of the polymer chain, thereby indirectly indicating the successful modification of the material [44].

Among the thermal parameters determined by DSC, the glass transition temperature is particularly important, as it reflects the transition of amorphous polymer domains from a rigid glass-like state to a flexible rubber-like state. This transition significantly affects the elasticity, mechanical stability, and molecular mobility of polymer scaffolds, thereby influencing their performance during implantation and tissue regeneration [44]. In contrast, the melting temperature characterizes the stability of crystalline regions and determines the processing conditions for semi-crystalline polymers, which are widely used in tissue engineering. Monitoring changes in Tg and Tm following the incorporation of drug compounds or modifications to the polymer’s chemical composition provides valuable information about the organization of smart polymer matrices and their suitability for biomedical applications [44,45].

**Figure 4:**
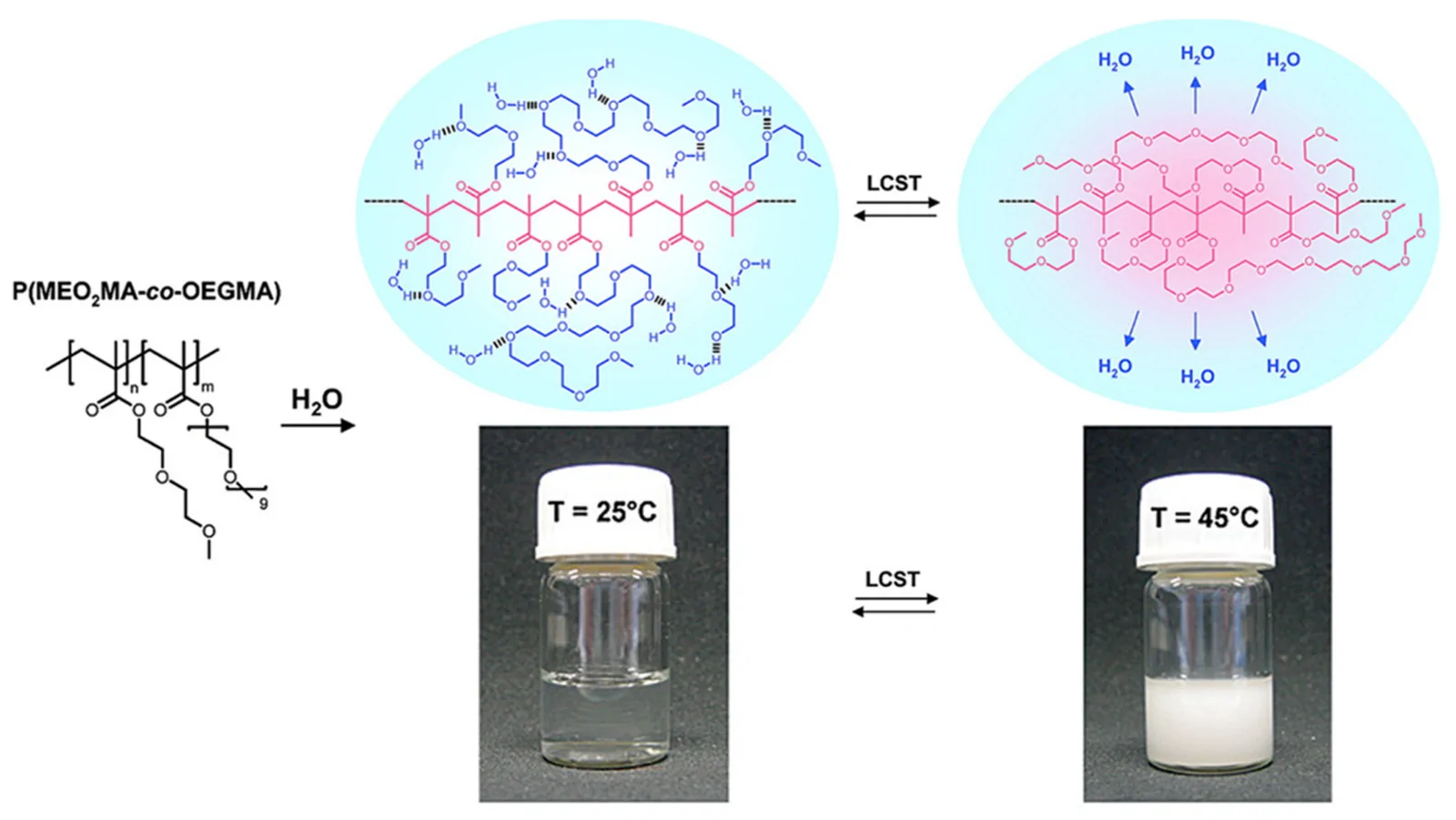
Examples of properties of LCST polymers [44]. Distributed under the terms and conditions of the Creative Commons Attribution (CC BY) license.

For thermosensitive polymers, particular attention is paid to the lower critical solution temperature (LCST), which defines the temperature at which the polymer undergoes a reversible transition from a hydrated, soluble state to a denatured, hydrophobic structure. An example of the properties of LCST polymers is presented in Figure 4. Since in many biomedical applications this transition must occur at a temperature close to the physiological temperature, precise thermal characterization is essential for optimizing the polymer composition and predicting its in vivo performance. Minor changes in the copolymer composition, molecular weight, or incorporated functional groups can significantly shift the LCST, thereby affecting the drug release kinetics, gel formation, and interaction with the surrounding tissues [44,45].

Thermogravimetric analysis (TGA) complements DSC by assessing the thermal stability of polymeric materials through continuous monitoring of mass loss during controlled heating [44]. This method allows for the determination of the onset temperature of degradation, the decomposition profile, and the residual mass, providing important information about the polymer’s stability during processing, sterilization, and long-term storage. In biomaterials containing incorporated drugs or bioactive molecules, TGA is also used to assess how small molecules alter the thermal stability of the matrix or the mechanisms of degradation [44,45]. Furthermore, comparing the thermogravimetric profiles before and after the material modification provides additional evidence of successful incorporation of functional components and changes in the organization of the polymer network [45].

Dynamic mechanical analysis (DMA) provides additional information by characterizing the temperature-dependent viscoelastic properties of polymeric materials. Unlike DSC, which detects thermal transitions via heat flow, DMA evaluates changes in the elastic modulus, loss modulus, and damping ratio during heating, allowing for highly sensitive determination of the glass transition temperature as well as the characterization of the mechanical response under physiologically relevant thermal conditions. For smart biomaterials, these measurements are particularly important, as temperature-induced changes in viscoelasticity directly affect the stability of the scaffold, its injectability, and the structural integrity after implantation. Furthermore, DMA allows for the assessment of the crosslinking efficiency and network architecture, which strongly influence the long-term performance of scaffolds for tissue engineering [44].

Taken together, DSC, TGA, and DMA provide additional information describing the thermal behavior of smart polymeric materials. These methods establish a relationship between polymer structure and functional characteristics, contributing to the rational design of thermosensitive biomaterials [44,45,46].

### 3.5. Degradation Behavior

The behavior of polymeric biomaterials during degradation is one of the key factors determining their clinical efficacy. Degradation influences the mechanical properties of the scaffold, the kinetics of therapeutic agent release, and the integration of the implant into tissue [47]. Unlike the static properties of the material, this process is dynamic in nature and is accompanied by a reduction in molecular weight, changes in porosity and the accumulation of degradation products, which together determine the body’s response and the effectiveness of regeneration [47,48].

An ideal biodegradable scaffold should degrade at a rate comparable to that of new tissue formation, gradually transferring its mechanical function to the new tissue. At the same time, the degradation products must be non-toxic and safely excreted or metabolized by the body [49,50]. Therefore, understanding the mechanisms of hydrolytic and enzymatic degradation, as well as the factors influencing the kinetics of the process, changes in pH and mass loss, is crucial for the rational design of polymer scaffolds and for the prediction of their behavior in vivo [47,48,49,50].

Hydrolytic degradation is the primary mechanism of degradation for most synthetic biodegradable polyesters used in tissue engineering. It occurs through the cleavage of ester bonds by water molecules without involvement of enzymes [47,49,50]. Hydrolysis is characteristic of polyesters, polyanhydrides, polyortho-esters, polycarbonates and a number of other biodegradable polymers [49]. The rate of the process is determined by the chemical structure of the material, its molecular weight, degree of crystallinity, hydrophilicity, porosity and cross-linking density, as well as environmental conditions [47,48,49]. Hydrophilic and amorphous polymers generally degrade more rapidly due to easier water penetration and high mobility of the polymer chains. Depending on the material’s properties, hydrolysis can lead to surface or volumetric erosion, accompanied by changes in the polymer’s morphology and crystallinity [48,49].

For aliphatic polyesters such as PLA, PLGA and PCL, hydrolytic degradation is driven by the cleavage of ester bonds. The rate of the process depends mainly on crystallinity, molecular weight and the autocatalytic action of acidic degradation products [47,49]. PLA is characterized by relatively slow degradation (around two years), which is due to its hydrophobicity and the absence of reactive side groups. PCL also degrades slowly (1–2 years); however, due to its higher crystallinity and hydrophobicity, it generally degrades more slowly than PLA [47]. In the case of PLGA, the rate of hydrolysis can be controlled by varying the ratio of lactic to glycolic acid: increasing the glycolic acid content accelerates degradation by increasing the hydrophilicity of the copolymer [47,49].

Enzymatic degradation is a biologically mediated process in which the polymer chains are cleaved by specific enzymes. Compared with hydrolysis, it often proceeds more rapidly and is characterized by greater specificity [47,48,50]. Degradation usually begins in the amorphous regions of the polymer, where increased mobility of the chains facilitates access by enzymes. As the amorphous phase breaks down, the relative proportion of crystalline domains increases, leading to a gradual slowing of the process. High crystallinity restricts the diffusion of water and enzymes; therefore, crystalline regions are significantly more resistant to enzymatic action [48].

The effect of molecular weight on the rate of enzymatic degradation is well illustrated by silk fibroin (SF) scaffolds. Scaffolds obtained after degumming with bromelain (142.2 kDa) degraded significantly more slowly than the materials treated with urea (126.4 kDa) or Na_2_CO_3_ (82.4 kDa). After 18 days, the residual mass was 33.3%, 11.0% and 4.0%, respectively. According to the scanning electron microscopy data, the three-dimensional structure of the high-molecular-weight scaffolds was preserved for up to 12 days, whereas the low-molecular-weight samples had already disintegrated after 6 days. This increased stability is attributed to the preservation of the integrity of the polymer chains and a more stable β-sheet structure, which restricts enzyme access [50].

A similar relationship is observed for polycaprolactone (PCL)-based composites, where the rate of enzymatic degradation is determined not only by the properties of the polymer itself, but also by the composition of the composite. Pseudomonas lipase hydrolyses the ester bonds of PCL after adsorption onto its surface, and the loss of material mass follows first-order kinetics. The addition of hydrophilic components (gelatine, collagen or PLGA) alters the rate of degradation by changing the wettability of the surface. For example, the PCL/gelatine (2:1) composite degraded almost as rapidly as pure PCL, as the rapid dissolution of gelatine led to the formation of micropores and facilitated enzyme penetration. In contrast, in the PCL/gelatine (3:1) system, degradation was slowed down due to the retention of gelatine on the surface. For composites with collagen and PLGA, the opposite relationship was observed, due to the specific characteristics of their swelling and dissolution [47].

Mass loss is one of the main quantitative indicators of polymer degradation, reflecting the release of soluble degradation products. It is usually assessed gravimetrically by measuring changes in residual mass over time [47,49,50]. A study of silk fibroin scaffolds showed that the rate of mass loss is inversely proportional to the molecular weight of the material: after 18 days of enzymatic degradation, the residual mass of the bromelain-, urea- and Na_2_CO_3_-treated scaffolds was 33.3%, 11.0% and 4.0%, respectively [50]. A similar pattern was observed for GelMA copolymers: an increase in the degree of methacrylation and cross-linking density slowed down the degradation. Following treatment with collagenase I, the L-GelMA scaffolds degraded completely within 24 h, whereas the H-GelMA scaffolds lost only about 45 per cent of their mass. The additional incorporation of VP and HEMA further increased the material’s resistance to enzymatic degradation [49].

Changes in pH are an important parameter in the degradation, as the accumulation of acidic hydrolysis products can cause local acidification of tissues, an inflammatory response, and accelerate further degradation of the material via autocatalysis [47,48,49]. For instance, the degradation of PLA and PLGA produces lactic and glycolic acids, whilst the hydrolysis of PCL is also accompanied by the formation of acidic products [47]. In contrast, polyanhydrides form non-toxic degradation products, and changes in pH can be regulated by their chemical composition. It should be noted that in physiological fluids in vitro, the degradation of most biodegradable polymers is predominantly driven by hydrolysis, whereas in vivo the presence of enzymes significantly accelerates this process [49].

Degradation kinetics describes the rate at which a polymer breaks down and is usually modeled using first-order exponential decay or more complex models that take into account hydrolysis, water diffusion and autocatalysis [47,48,49]. For PCL-based composites, mass loss is well described by first-order kinetics, and the degradation rate constant depends on the material composition and its wettability [47]. For example, PCL/gelatine composites degrade more rapidly than materials containing collagen or PLGA, reflecting the influence of the composite’s composition on the accessibility of the polymer chains to enzymatic hydrolysis [47]. Similarly, scaffolds made from the high-molecular-weight silk fibroin exhibited slower degradation kinetics compared with their low-molecular-weight counterparts [50].

The rate of enzymatic degradation is largely determined by the morphology of the polymer. Materials with a low degree of crystallinity and a high proportion of amorphous phase degrade more rapidly due to the greater mobility of the chains and the accessibility of hydrolysable bonds to the enzymes. This effect is most pronounced at temperatures above the glass transition temperature (Tg) but below the melting temperature (Tm), when the polymer chains become more mobile whilst retaining their structural integrity. Conversely, high crystallinity slows down degradation by limiting the penetration of water and enzymes. Furthermore, the rate of degradation may vary depending on the polymer architecture: random copolymerization typically accelerates degradation due to reduced crystallinity, whereas block copolymers exhibit more complex behavior due to phase separation [48].

A comprehensive assessment of degradation, including an analysis of the hydrolytic and enzymatic mechanisms, mass loss, changes in pH and the kinetics of the process, enables the rational design of polymer scaffolds for tissue engineering [47,48,49,50]. The rate of degradation must match the rate of tissue regeneration so that the implant retains its mechanical function during the early stages of recovery and gradually transfers this function to the newly formed tissue [49]. Thus, scaffolds for bone regeneration must retain their structural integrity for 6–8 weeks, whereas materials for skin regeneration must degrade in approximately 28 days [49,50]. The ability to regulate degradation kinetics by varying the polymer composition, molecular weight, degree of crystallinity and cross-linking density makes polymeric materials a versatile platform for the development of matrices with specified properties.

A promising area of research is the development of biodegradable polymers with immobilized enzymes. The incorporation of enzymes into the polymer matrix in the form of nanoparticles allows for the initiation of controlled degradation of the material and the achievement of virtually complete depolymerization under appropriate conditions. This approach opens up possibilities for the creation of polymer scaffolds with a programmable service life that more accurately corresponds to the rate of tissue regeneration [48].

### 3.6. Release-Related Properties

**Figure 5:**
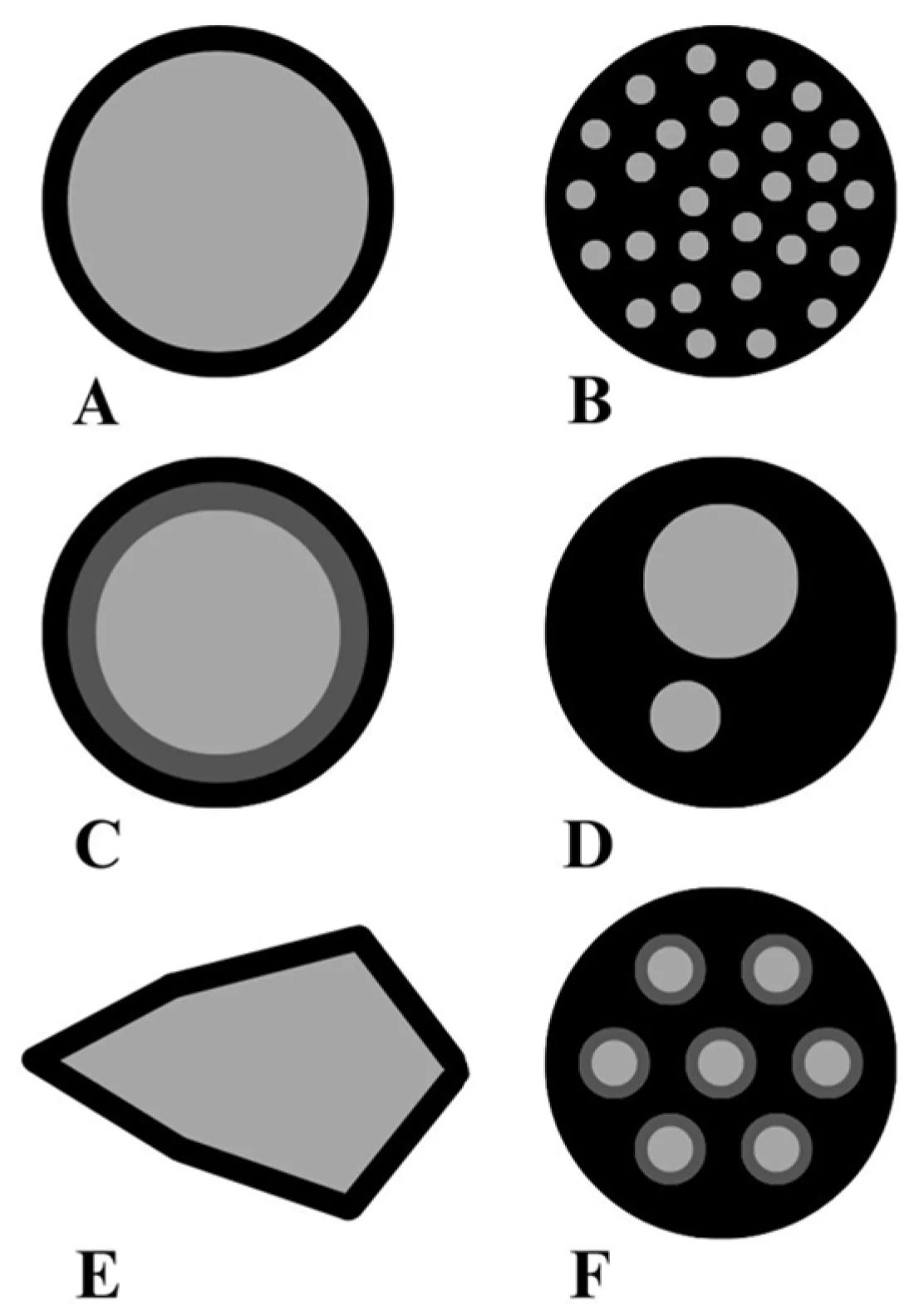
Some polymer capsules for drug delivery developed to date: (**A**) simple microcapsule, (**B**) microsphere, (**C**) multiwall microcapsule, (**D**) multicore microcapsule, (**E**) irregular microcapsule, (**F**) assembly of microcapsules. The polymer layer is represented in black and dark gray and the light gray represents the active substance [53]. Distributed under the terms and conditions of the Creative Commons Attribution (CC BY) license.

The capacity of polymeric scaffolds to serve as delivery vehicles for biologically active small molecules constitutes a central attribute of modern smart biomaterials, enabling the spatiotemporal control of therapeutic agent release and the modulation of the local biological microenvironment. Unlike conventional drug administration routes that often result in systemic toxicity and suboptimal therapeutic concentrations, polymeric delivery systems offer the potential for sustained, targeted, and stimuli-responsive release, thereby enhancing therapeutic efficacy while minimizing adverse effects [51,52]. The release behavior of the incorporated small molecules is governed by a complex interplay of factors encompassing the physicochemical properties of the polymer matrix, the encapsulation efficiency, the diffusion characteristics of the therapeutic agent, and the responsiveness of the system to environmental stimuli. Some examples of polymer capsules for drug delivery are shown in Figure 5. Consequently, a comprehensive understanding of the release-related properties—including the loading efficiency, encapsulation efficiency, release kinetics, diffusion mechanisms, stimuli-triggered release, and the influence of matrix properties on release—has become indispensable for the rational design of polymeric drug delivery systems for tissue engineering applications [51,52,53,54].

Loading efficiency (LE) and encapsulation efficiency (EE) represent fundamental parameters that quantify the capacity of a polymeric matrix to incorporate and retain therapeutic agents, directly influencing the dosing regimen and the therapeutic efficacy of the delivery system [51,52,54]. As defined in the pharmaceutical literature, drug loading efficiency is calculated as the ratio of the amount of drug incorporated into the carrier to the total weight of the carrier, while the encapsulation efficiency represents the percentage of the initially added drug that is successfully entrapped within the polymeric matrix [51,54]. The study on bacterial cellulose/bovine serum albumin nanoparticles (BSANP) for 5-fluorouracil (5-FU) delivery provides quantitative data illustrating the influence of carrier architecture on these parameters. The 5-FU@BC system achieved a loading efficiency of 25.49% and an encapsulation efficiency of 47.58%, while the dual biopolymer system 5-FU.BSANP@BC exhibited significantly higher values of 35.30% and 65.89%, respectively. This enhanced encapsulation performance was attributed to the synergistic combination of proteinaceous nanoparticles, which provide high drug-binding capacity, and the three-dimensional nanofibrous bacterial cellulose network, which offers abundant surface hydroxyl groups for drug immobilization [51].

The review on amoxicillin trihydrate encapsulation in biopolymer matrices further elucidates the role of polymer composition and crosslinking density in encapsulation efficiency. Alginate-chitosan matrices demonstrated superior encapsulation efficiency (>85%) compared with pectin-based formulations, which exhibited higher moisture sensitivity and faster drug release. This differential performance was attributed to the formation of a denser, more crosslinked polymer network in the alginate-chitosan system, which effectively limits drug diffusion and protects the therapeutic agent from environmental degradation [52]. The encapsulation of probiotics in alginate-starch beads similarly demonstrated that the combination of biopolymers can enhance encapsulation efficiency from 64.4% for pure alginate to 77% for the composite system, highlighting the importance of the matrix composition in determining loading capacity. The review on the encapsulation in natural polymer coatings emphasizes that the encapsulation efficiency depends on non-covalent interactions that may develop inside structures with cavities such as cyclodextrins, and that the entrapment depends on the specific structure of the active agent utilized [53]. Of particular interest is an approach that combines the computational prediction of binding with its experimental verification, implemented by a team from the Institute of Biochemical Physics of the Russian Academy of Sciences in collaboration with the Faculty of Chemistry at Moscow State University, using PLGA nanoparticles with carboplatin derivatives as an example. Using molecular dynamics docking on a PLGA matrix, the authors preliminarily estimated the binding energy of four synthesized platinum complex ligands to the polymer. Experimental verification using ICP-MS confirmed this trend: the incorporation efficiency (EE) for the derivative with a long alkyl chain reached 92 ± 2%, which is several times higher than the EE of carboplatin (54 ± 3%) and the other derivatives (60–62%), while the loading efficiency (DL) increased from 0.16% to 1.07%. The authors explicitly point out a limitation of the method: despite the good correlation between the calculated binding energy and the experimental EE/DL values, the increased lipophilicity of the synthesized derivatives limits their water solubility and requires the development of a special dosage form for parenteral administration when transitioning to in vivo studies. Overall, the described low initial EE of platinum drugs (<50%) in polymeric carriers and the strategy for increasing it through ligand structure modification are consistent with the practice, widely described in the international literature, of increasing the hydrophobicity of the encapsulated substance to enhance the EE in PLGA-based systems [55].

Release kinetics describe the temporal evolution of the drug liberation from the polymeric matrix, typically characterized by an initial burst release followed by sustained release phases governed by diffusion, polymer relaxation, or degradation mechanisms [51,52,54]. The study on 5-FU delivery systems provides a comprehensive kinetic analysis using the zero-order, first-order, and Korsmeyer–Peppas models. Free 5-FU exhibited zero-order kinetics (R^2^ = 0.97), indicating concentration-independent release, whereas the encapsulated formulations followed first-order kinetics with R^2^ values exceeding 0.97 for 5-FU.BSANP, 5-FU@BC, and 5-FU.BSANP@BC. The Korsmeyer–Peppas model revealed *n* values of 0.35 and 0.42 for 5-FU.BSANP and 5-FU.BSANP@BC, respectively, indicating Fickian diffusion (*n* < 0.43), while 5-FU@BC exhibited *n* = 0.49, signifying non-Fickian diffusion where both drug diffusion and polymer chain relaxation contribute to the release. The presence of BSANPs within the BC network appears to suppress polymer relaxation, shifting the mechanism toward Fickian diffusion [51].

The release profile of 5-FU.BSANP@BC demonstrated an initial burst release of approximately 14% within the first 6 h, followed by a sustained release phase reaching 37% over 358 h, significantly more prolonged than that reported for other bacterial cellulose-based systems. The drug release rate decreased in two stages from the BSANP carrier—initially due to drug diffusion from within the nanoparticles, and subsequently due to the gradual degradation of nanocarriers during the release study—whereas the cellulose networks exhibited a decline after the initial burst followed by stabilization [51]. The amoxicillin study similarly reported biphasic release patterns in alginate-chitosan systems, characterized by an initial burst followed by sustained release predominantly governed by diffusion and polymer relaxation mechanisms. Mathematical modeling of the release data indicated that the Higuchi model provided the best fit for most formulations (R^2^ > 0.98), confirming a diffusion-controlled mechanism [52].

Diffusion mechanisms govern the transport of therapeutic agents through the polymeric matrix, with Fickian and non-Fickian diffusion representing the two primary modes of release [51,54]. As elucidated in the review on active packaging, the release of an active compound from a polymer matrix is typically described by Fick’s law, which relates the flux of the diffusing species to the concentration gradient [54]. The Peppas equation, with its release exponent (*n*), provides a quantitative framework for distinguishing between diffusion mechanisms: *n* < 0.43 indicates Fickian diffusion (Case I), *n* between 0.43 and 1.0 indicates non-Fickian or anomalous transport (Case II), and *n* approaching 1.0 indicates zero-order kinetics [51,54]. In the context of hydrogel swelling, the Korsmeyer–Peppas equation has been widely applied to analyze water uptake kinetics, as the uptake of water into the polymer network follows identical Fickian or non-Fickian diffusion mechanisms [51].

The review on the controlled release in active packaging emphasizes that the diffusion coefficient increases exponentially with an increase in the particle size, and an even distribution of particles is important for the controlled release. The degree of crystallinity affects the release rate due to a reduction in the length of the migration path, with higher crystallinity generally reducing release rates. The inclusion of nanofillers reduces the polymer torsion and film flexibility, reducing the release rate due to an increase in the diffusion path length. Plasticizers such as ethylene glycol and glycerol increase the release and diffusion rates due to an increase in free volume inside the polymer matrix. The polarity of the film and the active compound affects the release rate depending on the interaction strength between the two components, with strong non-covalent interactions between the hydrophobic polymer matrices and low polarity active compounds resulting in slow-release rates [54].

Stimuli-triggered release represents an advanced paradigm in drug delivery, wherein the release of therapeutic agents is activated or modulated by specific environmental stimuli such as pH, temperature, enzymes, or magnetic fields [52,53,54]. The review on hydrogels for drug release and tissue engineering provides a comprehensive overview of responsive hydrogels, including pH-responsive, temperature-responsive, electric field-responsive, ion-responsive, magnetic field-responsive, pressure-responsive, photo-responsive, biomolecule-responsive, and redox-responsive systems. pH-responsive hydrogels, which contain acidic or basic groups capable of ionization, have been extensively investigated for targeted drug delivery to the gastrointestinal tract and cancerous tissues where pH values differ from the normal physiological state. The mechanism involves the protonation or deprotonation of functional groups, leading to hydrogel swelling or shrinking and the consequent controlled drug release [52].

Temperature-responsive hydrogels, such as those based on poly(N-isopropylacrylamide) (PNIPAM), undergo phase transition at the lower critical solution temperature (LCST), enabling drug release triggered by temperature changes [52,53]. The review on the encapsulation in natural polymer coatings describes the temperature-responsive behavior of PNIPAM-based microgels, where the hydrodynamic radius changes due to the deprotonation or protonation of the chemical functions in response to temperature variations at different pH values [53]. Enzyme-responsive hydrogels, which degrade in the presence of specific enzymes such as collagenase, protease, or esterase, offer targeted release at the sites of inflammation or disease [52,54]. The review on the active packaging highlights the potential of enzyme-responsive films that release natural antimicrobials in response to humidity and enzyme triggers, with the enzymatic degradation of zein enhancing the release of active ingredients from electrospun fibers [54]. Multi-responsive hydrogels combining two or more triggering mechanisms have attracted considerable attention because they enable cascade-controlled drug release and more accurately reproduce the complexity of pathological microenvironments [52].

The physicochemical properties of the polymer matrix—including the swelling behavior, crosslinking density, hydrophilicity, crystallinity, and degradation kinetics—profoundly influence the release characteristics of the incorporated small molecules. The study on bacterial cellulose nanocarriers demonstrates that hydrogel swelling plays a critical role in drug delivery systems, as it significantly affects the diffusion kinetics of pharmaceutical agents into the network. The BC hydrogel swelled to approximately 34.8 times its original weight, with the swelling process exhibiting a time-dependent increase until 24 h, at which point equilibrium was attained. This swelling behavior, governed by Fickian diffusion (*n* = 0.074), directly influences the loading capacity and release kinetics of the 5-FU delivery system. The swelling of the hydrogel facilitates water ingress, which in turn promotes drug diffusion and release, while the degradation of the BC network (approximately 11% over 21 days) contributes to the sustained release profile [51].

The review on amoxicillin encapsulation emphasizes that the degree of methacrylation of gelatin methacryloyl (GelMA) hydrogels significantly influences swelling behavior and degradation rates. Higher methacryloylation degrees lead to increased crosslinking density, reduced swelling, and slower enzymatic degradation, enabling the modulation of drug release kinetics through the control of the polymer network structure. The incorporation of hydrophilic comonomers such as vinylpyrrolidone (VP) and 2-hydroxyethylmethacrylate (HEMA) into GelMA copolymers further tunes swelling and degradation properties, allowing sustained drug release over extended periods [52]. The review on the encapsulation in natural polymer coatings highlights that the swelling ability of pectin hydrogels and the associated larger pore size under physiological conditions could be a limitation for certain lower molecular weight bioactive compounds [53]. The moisture affinity of pectin was noted as a factor facilitating the limited hydrolysis within the matrix, accounting for the differences in the stability relative to alginate-chitosan systems [52].

The review on the controlled release in active packaging identifies particle size and distribution as having the most significant effect on the retention inside the polymer matrix and hence the release rate. The diffusion coefficient increases exponentially with an increase in the particle size, and an even distribution of particles is important for the controlled release. The molecular weight of the active agent and its affinity to the polymer are important parameters for the effective controlled release. Polarity effects of the film and the active compound may affect the release rate depending on the interaction strength between the two components. Strong non-covalent interactions between hydrophobic cellulose acetate films and low polarity active compounds would result in slow-release rates. Furthermore, the external environment influences the release rate, with temperature and humidity affecting the plasticization of the polymer films and consequently the diffusion of the active compounds [54].

The comprehensive characterization of the release-related properties—encompassing loading efficiency, encapsulation efficiency, release kinetics, diffusion mechanisms, stimuli-triggered release, and the influence of matrix physicochemical properties—enables the rational design of polymeric scaffolds with drug delivery profiles tailored to specific therapeutic applications. The integration of these parameters with biological assays enables the correlation of material properties with therapeutic outcomes, facilitating the identification of the optimal scaffold formulations for tissue engineering applications.

## 4. Release Profiles and Mechanisms

**Figure 6:**
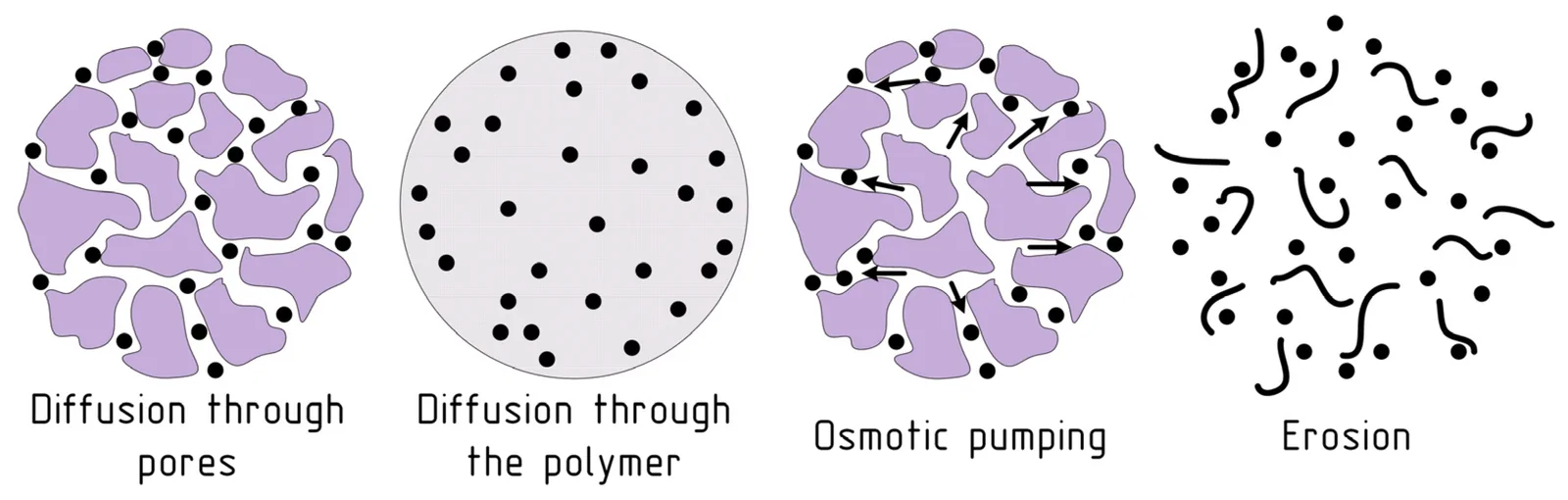
Key drug release mechanisms: diffusion through pores, diffusion through the polymer, osmotic pumping, and erosion. Redrawn from [56].

While the preceding section addressed the release-related properties primarily from the perspective of their characterization and the factors governing drug release, this section focuses on the underlying transport mechanisms responsible for the release of therapeutic agents from polymeric matrices. For polymeric drug-loaded materials, the terms “drug release” or “release mechanism” describe the process and rate of loaded molecules transport from the polymeric matrix into the surroundings [56,57]. The key release mechanisms can be divided into the following four “true” (ideal) release mechanisms: diffusion through pores, diffusion through the polymer, osmotic pumping, and erosion [57] (Figure 6).

Diffusion through pores is based on the random movement of drug molecules under the action of a chemical potential gradient, which can usually be described as a concentration gradient. The drug release rate depends on the drug diffusion through the pores into the polymeric system, and the structure of such pores changes as the polymer matrix degrades. For example, in the case of polymer nanoparticles, water absorption occurs drastically faster than drug release. Consequently, during the first stage, absorbed water creates internal pores in the polymer matrix, which further increase in diameter and number, allowing free transport of drug molecules [56,57].

In the case of diffusion through the polymer, drug release occurs due to diffusion process within the polymer matrix. Diffusion transport is a key limiting factor for drug release. The release rate for non-degradable and non-porous reservoir drug delivery systems is constant, but it can be changed by the properties of the polymer matrix and the structural and dimensional parameters of the drug delivery system [57].

The third transport mechanism, through the water-filled pores, is based on convection. Water permeation into a non-swelling polymer system is caused by the osmotic pressure, and the drug transfer is classified as osmotic pumping [57].

The erosion mechanism is based on the ratio between polymer chain destruction and the rate of the water permeation and can be divided into surface erosion and bulk erosion. The first case occurs at the phase boundary “polymer system–surrounding environment” when the rate of polymer degradation is much higher than that of water diffusion into its volume. This process leads to a gradual decrease in the geometrical dimensions of the polymer matrices. This mechanism is preferable for the modern drug delivery systems due to its decidability and protective effect on the encapsulated drug [57].

Bulk erosion is characterized by the uniform bulk destruction of polymers. In this case, water diffusion occurs at a higher rate than the hydrolysis of macromolecules. Bulk erosion is less predictable and does not protect the loaded drugs from the aggressive environmental conditions, and for this reason, it is not recommended for the prolonged or controlled drug delivery systems [57].

It is known that the development of a polymeric system having a single, ideal, and “true” drug release mechanism is impossible due to the complex processes taking place in the polymer matrix in contact with aqueous or biological fluids. Such processes include (but are not limited to) swelling, erosion, relaxation, degradation, and structural changes in the polymer system. At the same time, several examples of polymeric systems in which one release mechanism is primary are briefly discussed below.

### 4.1. Diffusion Through Pores

Porous microspheres based on ethyl cellulose and polyvinyl alcohol loaded with quercetin were prepared by the quasi-emulsion solvent diffusion method. The obtained microspheres were incorporated into a gel based on carbomer 934 to modulate the release kinetics, enhance the skin adhesion and capacity. In this case, ethyl cellulose acts as a non-swelling polymer matrix, while polyvinyl alcohol stabilizes the emulsion and acts as a pore-forming agent and a drug diffusion regulator [58].

Key limitations of this mechanism include:

- Percolation threshold and critical porosity. Below the lower percolation threshold, the drug release is frequently incomplete, as the active substance remains encapsulated and entrapped within the isolated pores of the polymer matrix [59,60].
- Scalability problems. Scaling up of the fabrication process from the laboratory to the semi-industrial and/or industrial batches may be associated with the pore reproducibility and material inhomogeneity, as well as with other quality problems [61].
- Low diffusion coefficients for high-molecular-weight substances. Low-molecular-weight substances can fill pores more effectively during the loading procedure than high-molecular-weight proteins, because small proteins are able to penetrate to the internal surfaces, whereas large proteins are restricted to the external surfaces [62].

### 4.2. Diffusion Through the Polymer

Cellulose acetate propionate, cellulose acetate phthalate and their mixtures were successfully used for the encapsulation of quercetin. The developed system has gastro-resistance and pH-sensitive drug release behavior, which is useful for the local delivery of hydrophobic pharmacological agents to the intestine via oral administration [63].

Key challenges of the diffusion mechanism through the polymer include:

- Swelling and relaxation effects. In the case of hydrophilic polymeric matrices, drug diffusion is accompanied by polymer swelling: water molecules penetrate into the polymer matrix and polymer chains relax. This process alters the diffusion mechanism due to the enlargement of the diffusion path. Moreover, the diffusion coefficient becomes dependent on the swelling ratio [64,65].
- Burst release. The rapid burst release may be associated with the weakly bound or absorbed small molecules on the surface of the polymer matrix [66].
- Low diffusion coefficients for high-molecular-weight substances. Diffusion coefficients of myoglobin within the poly(ethylene glycol)-diacrylate (PEG-DA) hydrogels ranged from one to two orders of magnitude lower than the diffusion coefficients in water [67].

### 4.3. Osmotic Pumping

**Figure 7:**
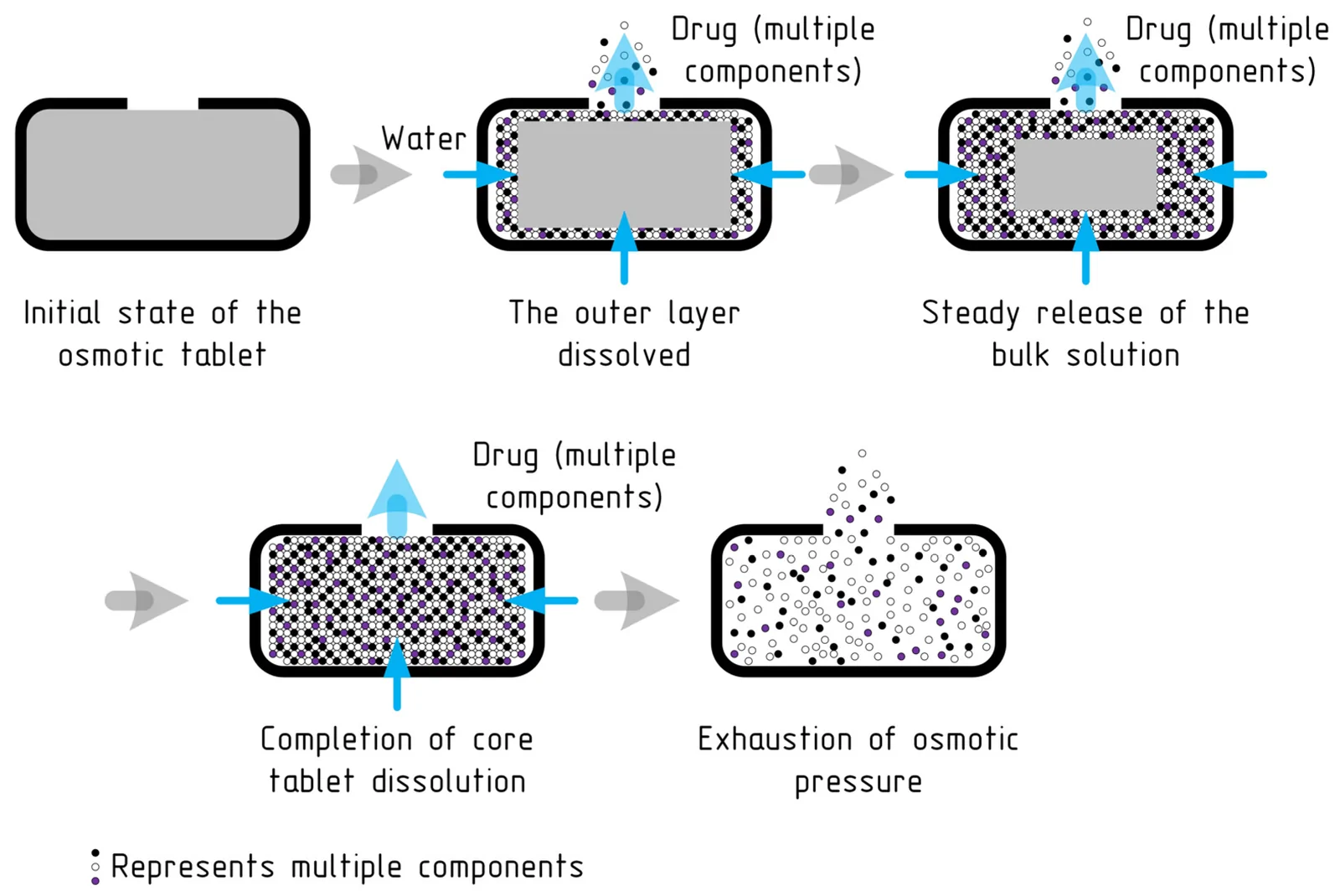
Schematic representation of the synchronized and sustained release of natural components from the osmotic system. Redrawn from [68].

Multiple polyphenol components in silymarin (from *Silybum marianum* L. Gaertn), consisting of taxifolin, silychristin, silydianin, silybin, and isosilybin, have varying aqueous solubilities and drug release kinetics, which hinder the development of drug delivery systems based on it. A monolithic osmotic tablet perforated with the release orifices and containing the solid dispersion of silymarin demonstrates the synchronized and controllable release kinetics of the mixture’s components. Polyvinylpyrrolidone was used as a solid dispersion agent. Sodium, agglomerated lactose, and mannitol were used as osmotic agents. Crospovidone was incorporated into the core of the osmotic system to improve water imbibition. Cellulose acetate and polyethylene glycol 4000 was used for the semi-permeable [68]. Schematic representation of the synchronized and sustained release of the natural components from the osmotic composition is depicted in Figure 7.

Osmotic systems have scientific challenges and commercial limitations:

- Technological complexity. The requirement of precise membrane coating, controlled drilling of the orifice, multilayered compression, and stringent dimensional uniformity collectively increase both manufacturing complexity and cost.
- High manufacturing cost. In comparison with conventional tablets, osmotic dosage forms necessitate specialized materials, fabrication techniques and equipment.
- Risk of rapid (burst) release. Membrane degradation or fabrication defects may alter the drug release profile.
- Restricted suitability for high-dose drugs. Drugs administered at very high doses may require larger capsule dimensions, thereby reducing patient acceptability.
- Solubility-related limitation. Hydrophobic drugs may require the use of solubility enhancers or the adoption of push-pull design.
- Susceptibility to ambient moisture. Certain osmotic systems require controlled packaging to maintain stability [69].

### 4.4. Erosion

Ouimet M.A. et al. used poly(anhydride-ester) as a hydrolytically degradable polymer matrix. Ferulic acid was incorporated into the polymer system by covalent binding. The drug transport in this case has the erosion mechanism—only during polymer degradation the release occurs over 30 days [70].

In spite of the prolonged release profile, the erosion mechanism has several limitations. Thus, polymer systems based on PLGA undergo bulk erosion instead of surface erosion [71], which alters the release profile and may lead to burst drug release. Secondly, there is a “competition” between drug diffusion and polymer erosion, which hinders the prediction of the drug release profile. Polymer molecular weight, as well as drug nature, can alter the dominant release mechanism and complicate the speed matching [56]. Thirdly, in the case of the PLGA-based systems, the autocatalytic degradation can lead to the accumulation of acidic oligomers, a pH gradient, accelerated bulk erosion, and size-dependent inhomogeneity, which alter total drug release [56,72].

The aforementioned limitations and inevitable deviations of the real drug delivery systems from the idealized mechanisms require the use of mathematical tools and approximations capable of quantitatively describing and predicting release kinetics, while accounting for the combined effect of the above-mentioned key mechanisms. Key mathematical models (zero-order, first-order, Higuchi, Korsmeyer–Peppas, Hixson–Crowell, and Weibull) widely used for describing drug release from polymer matrices are presented in Table 1.

## 5. Fabrication and Small-Molecule Incorporation Strategies in Polymeric Smart Materials

While Section 2 outlines the general principles and design criteria governing the selection of polymeric matrices and small-molecule loading approaches, the present section considers their practical implementation, focusing specifically on material fabrication and the different strategies used to incorporate small molecules into polymeric matrices.

### 5.1. Scope and General Design Considerations

In this review, a polymeric smart material is a polymer-based construct whose chemistry, network structure, surface, or macroscopic architecture is designed to provide an adaptive function in a biological environment. The response may involve a change in swelling, permeability, degradation, adhesion, stiffness, shape, or molecular release. Passive sustained-release systems are included as reference platforms and as components of multifunctional materials, but the passive release alone should not automatically be described as smart [75,76].

The term small molecule is used here for a chemically defined low-molecular-weight organic compound, generally below approximately 1000 Da. This operational group includes approved drugs, natural bioactive compounds, metabolites, and synthetic regulators of signaling pathways. Proteins, nucleic acids, polysaccharides, cells, extracellular vesicles, and inorganic ions are outside the main scope of this section [77].

In this review, the preparation and manufacturing workflow includes all operations through which a polymer or polymer precursor is converted into the final clinically relevant material. Fabrication specifically refers to the formation of the material by processes such as crosslinking, casting, phase separation, electrospinning, extrusion, or additive manufacturing. Washing, drying, sterilization, packaging, and storage are treated as subsequent manufacturing stages because they can further change the polymer structure, cargo content, and functional performance. The loading describes the amount of compound associated with the material, whereas incorporation also describes its physical or chemical state. A molecule may be physically entrapped, reversibly bound, encapsulated in a secondary carrier, deposited on a surface, or covalently connected to the polymer. These states are not equivalent because they produce different spatial distributions, retention mechanisms, and possibilities for release [78,79,80].

The design sequence should begin with the requirements of the target tissue and the properties of the small molecule: target-tissue requirements and cargo properties → processing compatibility → incorporation strategy → material structure and cargo distribution → local presentation. The target tissue defines the required architecture, mechanical function, implantation route, degradation period, and the duration of molecular exposure. Molecular weight, charge, pKa, solubility, lipophilicity, and chemical stability determine whether the compound can be dispersed in the processing medium and whether it can tolerate heat, light, oxygen, organic solvents, reactive monomers, initiators, or crosslinkers. Polymer chemistry and material architecture then determine the available binding sites, network mesh size, swelling, porosity, crystallinity, degradation, and the ability to meet the tissue-specific mechanical requirements. A process suitable for the polymer may still damage the active compound, whereas a loading method that preserves the compound may interfere with material formation or mechanical performance [78,79,80,81].

After fabrication, release may be governed by diffusion, desorption, swelling, dissolution, polymer erosion, or a combination of these processes. Stimuli-responsive presentation requires an additional causal relationship: a defined endogenous or external signal must produce a measurable material change and a reproducible change in the molecular release or accessibility. Smart behavior and the mechanism of small-molecule release should therefore be reported separately. A shape-memory scaffold, for example, may be smart even if its drug release is passive, whereas a macroscopically stable material may provide triggered release through a cleavable linker or responsive carrier [75,81].

No single strategy is optimal for every polymer–molecule pair. The selected method should preserve chemical integrity, provide the required spatial distribution and exposure period, maintain the architecture and mechanics of the material, and remain compatible with sterilization, storage, reproducible manufacture, and scale-up. The following subsections first compare the incorporation strategies and then examine the constraints imposed by the main fabrication platforms.

### 5.2. Strategies for Small-Molecule Incorporation

#### 5.2.1. Direct Bulk Loading and Physical Entrapment

Direct bulk loading introduces the small molecule into a polymer solution, melt, or precursor mixture before the final material is formed. Gelation, crosslinking, solvent removal, phase separation, fiber formation, extrusion, or printing then trap the compound in the polymer matrix without a specific covalent bond. The method is attractive because cargo incorporation and material fabrication occur in one preparation sequence and do not require a separate carrier or loading step [79,80].

This approach is most suitable when the compound is soluble or stably dispersible in the processing medium and remains chemically stable throughout the fabrication. The amount initially added to the formulation should not be treated as the amount retained in the final construct. Loss can occur through precipitation, partitioning into a removed solvent phase, washing, porogen leaching, adsorption to processing equipment, or chemical degradation. At minimum, studies should distinguish theoretical drug content, actual drug content, and incorporation efficiency. Actual drug content may be expressed as the mass of the intact drug in the final dry material divided by the total dry mass of the loaded material. Incorporation efficiency may be calculated as the mass of intact drug retained in the final material divided by the mass initially introduced. When a drug-to-polymer ratio or another denominator is used, it should be stated explicitly. For carrier-containing systems, the drug loading in the isolated carrier and final drug content in the complete scaffold should be reported separately. A process mass balance should account, where possible, for the intact drug recovered in the final product, wash solutions, removed solvents, and other process streams [82,83].

The main limitation is the weak retention of low-molecular-weight compounds. In highly hydrated networks, many small molecules are much smaller than the network mesh and can diffuse rapidly unless they interact with the polymer. In solid fibers and porous scaffolds, rapid solvent evaporation or phase separation may move the compound toward the surfaces or create drug-rich regions. Hydrophobic molecules in aqueous precursors may aggregate or crystallize, whereas hydrophilic compounds may be poorly compatible with hydrophobic polymers. These effects produce non-uniform loading and an initial burst even when the nominal formulation is unchanged [79,84,85].

Direct loading also exposes the compound to the complete processing history. Relevant stresses include heat during melt processing, organic solvents during electrospinning and phase separation, radicals during photopolymerization, changes in pH, and shear or pressure during extrusion. The active compound may also change viscosity, gelation, fiber morphology, polymer crystallinity, or printing accuracy. Chemical integrity and material properties must therefore be measured after fabrication rather than inferred from the successful scaffold formation [86,87].

Direct entrapment is the simplest strategy to evaluate when the compound is compatible with the polymer and processing conditions and when the method provides uniform distribution, acceptable retention, and preserved activity. Affinity binding, secondary carriers, post-fabrication loading, or cleavable conjugation are justified when direct loading produces excessive burst release, poor dispersion, or chemical damage.

#### 5.2.2. Affinity-Based Incorporation and Molecular Complexation

Affinity-based incorporation introduces reversible non-covalent interactions between the small molecules and the binding sites in the polymer matrix. Electrostatic attraction, hydrogen bonding, hydrophobic association, ionic complexation, and host–guest inclusion can reduce the effective mobility of a molecule without permanent attachment. Release is controlled by both diffusion and repeated association–dissociation events, which distinguishes the affinity systems from simple geometric entrapment and covalent tethering [88,89].

Electrostatic retention is useful when the cargo and the polymer carry opposite charges. Its strength depends on the pKa values of both components, loading and release pH, ionic strength, and competing ions. Physiological salts may screen the interactions that appear strong in low-ionic-strength buffers, while changes in the local pH can alter both the loading and the release. A study of supramolecular peptide hydrogels showed that the relative charges of the cargo and the network strongly affected small-molecule release, supporting the need to test ionic systems under biologically relevant conditions [90].

Hydrogen bonding and hydrophobic association may also improve retention, especially when several complementary groups act together. However, these interactions compete with water and can change with hydration, temperature, or polymer degradation. Excessive hydrophobic affinity may produce incomplete release, aggregation, or crystallization. The proposed interaction should therefore be supported by suitable controls, such as a chemically similar matrix without the affinity group, binding measurements, or the release studies at different pH and ionic strength.

Cyclodextrin-containing materials are a well-established host–guest platform for hydrophobic or amphiphilic compounds. Cyclodextrins provide a less polar internal cavity and a hydrophilic exterior. They can be grafted to polymer chains or crosslinked as part of a network, creating repeated binding sites that increase apparent solubility and reduce effective diffusivity. The outcome depends on the cavity–guest compatibility, cyclodextrin substitution, site density, and accessibility [91,92]. Mealy et al. used β-cyclodextrin-modified hyaluronic acid hydrogels to extend the delivery of several hydrophobic small molecules, demonstrating that the reversible host–guest retention can prolong release without covalently modifying the drug [93].

The strongest possible affinity is not always optimal. Weak binding provides little improvement over physical entrapment, whereas very strong binding may leave only a small fraction available to the surrounding cells. Retention should therefore not be considered equivalent to biological availability. Where feasible, the total associated amount, reversibly bound fraction, and freely available fraction should be evaluated separately. Release experiments should use media with the relevant pH, ionic strength, proteins, lipids, or competing solutes because these components can change the binding equilibria and produce behavior that differs from release in simple buffers. Affinity systems should be optimized for reversible local presentation rather than maximum sequestration. Association strength, binding-site density, actual content, release completeness, and possible changes in swelling, mechanics, and degradation should be evaluated together [88,89,90,91,92,93].

#### 5.2.3. Secondary-Carrier-Assisted Incorporation

In carrier-assisted incorporation, the small molecule is first loaded into a discrete carrier, and the loaded carrier is then placed in a larger polymer matrix. The carrier may be a polymeric micro- or nanoparticle, liposome, micelle, nanogel, mesoporous particle, or supramolecular assembly. This hierarchical design separates the cargo-specific encapsulation from the scaffold-specific fabrication and is especially useful for poorly water-soluble, rapidly diffusing, or process-sensitive compounds [82].

The carrier provides a local chemical environment that may be more compatible with the compound than the main matrix. Hydrophobic molecules, for example, can be placed in lipid bilayers, micellar cores, polymeric particles, or mesopores and then dispersed in aqueous hydrogel or printing ink. The molecule must first leave or pass through the carrier and then move through the surrounding scaffold. These two transport levels can reduce rapid loss and enable multiphase release, although the matrix may also change the carrier hydration, aggregation, degradation, or accessibility.

Representative studies show the range of this strategy. Simvastatin-loaded PLGA microparticles were incorporated into porous chitosan–gelatin scaffolds for bone tissue engineering [94]. Liposomes containing curcumin and α-tocopherol were dispersed in injectable chitosan hydrogels for dental applications [95]. More recent examples include the sildenafil-loaded nanomicelles in a printed pectin–fucoidan scaffold and baicalein-loaded carboxylated mesoporous silica incorporated into methacrylated gelatin for vital pulp therapy [96,97]. These systems illustrate the ability to combine cargo-specific solubilization with tissue-specific material architecture.

Additional control is accompanied by additional variability. Carrier size, size distribution, surface charge, encapsulation efficiency, drug content, and colloidal stability must be controlled before incorporation. During mixing or printing, particles may aggregate, liposomes may lose integrity, and larger carriers may sediment before gelation. Carriers can also change the rheology, crosslinking, porosity, swelling, degradation, and mechanical properties. The final composite must therefore be characterized as a new formulation rather than predicted from the measurements of the isolated carrier and unloaded scaffold.

A useful mass balance should include the starting drug amount, the drug retained in the isolated carrier, loaded carrier introduced into the matrix, and the intact drug measured in the completed material. Release studies should compare the free molecule in the matrix, the loaded carrier alone, and the carrier-in-matrix system. The fate of the secondary carrier should be evaluated separately from the degradation of the surrounding polymer matrix. Relevant parameters include carrier dissolution or degradation, persistence in tissue, release of carrier degradation products, cellular uptake, local accumulation, and possible clearance. This issue is particularly important when a biodegradable hydrogel or scaffold contains a carrier with a substantially slower degradation rate, such as selected polymeric particles or mesoporous inorganic materials. Biological effects should therefore not be attributed only to the released small molecule when the carrier itself may influence cells, inflammation, mineralization, or material mechanics [82,94,95,96,97]. Carrier-assisted incorporation is justified when these controls demonstrate a clear advantage over direct or affinity-based loading.

#### 5.2.4. Post-Fabrication Loading by Diffusion and Impregnation

Post-fabrication loading is performed after the material has obtained its final or nearly final structure. A scaffold, membrane, fibrous mat, film, or implant is contacted with a solution of the small molecule, which enters the accessible pores or polymer domains by wetting, capillary transport, diffusion, and partitioning. Common approaches include soaking, dropwise loading, solution impregnation, and, for selected polymer–molecule pairs, supercritical-fluid-assisted impregnation [82].

The main advantage is the separation of cargo loading from scaffold formation. A sensitive compound can avoid heat, reactive species, or solvents used during the main fabrication step, and one preformed scaffold design can be loaded with different compounds or doses. Nevertheless, the loading medium may swell, plasticize, hydrolyze, or partly dissolve the polymer. The method should be considered mild only after the compatibility of the material, molecule, and solvent has been demonstrated.

The loading depends on the solution concentration and volume, incubation time, temperature, scaffold dimensions, wettability, pore connectivity, and polymer–drug partitioning. Molecules contact the external surfaces first, and insufficient loading time or poor wetting can produce a surface-enriched distribution. Drying may further move the dissolved cargo toward the exterior. Total content alone therefore does not establish uniform penetration; spatial analysis across the construct is needed, particularly for thick or architecturally complex materials [83].

Supercritical CO_2_ can transport compatible compounds into preformed polymers while reducing conventional solvent residues. CO_2_ sorption may plasticize the polymer and increase free volume, but the loading depends on drug solubility in the supercritical phase, CO_2_ uptake by the polymer, and favorable partitioning. Pressure, temperature, treatment time, and depressurization can also change the crystallinity, dimensions, or pore structure [98,99]. Direct comparison of soaking and supercritical impregnation has shown that the latter can increase loading for selected polymer–drug combinations, but the outcome is material-specific [100].

Post-fabrication loading is suitable when processing the sensitivity is the main limitation and when moderate, modular loading is sufficient. Its typical risks are limited depth, content variability, weak retention, and burst release. Affinity sites, coatings, or secondary carriers may be added when simple impregnation does not provide the required local exposure.

#### 5.2.5. Surface Deposition, Coatings, and Layer-by-Layer Assembly

Surface-based strategies place the small molecule on the external interface or within a thin coating rather than throughout the full material volume. In this section, surface deposition refers to the application of a molecule-containing solution or suspension followed by solvent removal, whereas adsorption refers to molecular association with an existing surface during equilibration in a loading solution. These processes should be distinguished from bulk post-fabrication impregnation, which aims to transport the molecule into the internal volume of a porous material. Surface localization is particularly useful when the intended function is concentrated near the material–tissue interface, for example, during early inflammation, bacterial colonization, cell adhesion, or fibrotic encapsulation [101,102,103].

After simple deposition, the molecule may remain molecularly dispersed, amorphous, crystalline, or weakly adsorbed. A polymeric or hydrogel coating provides a distinct surface phase in which diffusion, degradation, and polymer–molecule partitioning can be adjusted. Coating a three-dimensional porous scaffold is more difficult than coating a flat film because viscous solutions may accumulate near the exterior, block the pore entrances, or provide incomplete coverage of the internal surfaces. Coating thickness, spatial coverage, pore accessibility, and cargo distribution should therefore be measured throughout the construct rather than inferred from the applied solution volume. Stable and cleavable covalent attachments are considered separately in Section 5.2.6 because they change the mechanism from physical surface loading to contact-dependent presentation or chemically controlled release.

Layer-by-layer assembly builds a thin film through sequential adsorption of complementary components, commonly oppositely charged polyelectrolytes. Film composition, thickness, and molecular location can be changed through the number and order of layers, pH, ionic strength, deposition time, and crosslinking. A small molecule can be incorporated in selected layers, complexed with a polymer component, or loaded into the completed film. This creates opportunities for surface-localized and staged presentation, although the molecular exchange during assembly may reduce the intended spatial separation [102,103].

Gonçalves et al. compared dexamethasone incorporated during electrospinning with dexamethasone placed in the chitosan/heparin layer-by-layer coatings on PLLA membranes. The number of layers changed the release profile, illustrating that a surface film can act as both a local reservoir and a transport barrier [104].

The main quality attributes are coating mass and thickness, coverage of the external and internal surfaces, layer number and sequence, stability in physiological media, delamination, and changes in wettability, pore access, or mechanics.

#### 5.2.6. Covalent Tethering and Cleavable Polymer–Drug Conjugation

Covalent incorporation forms a chemical bond between the small molecule and a polymer chain, network component, surface group, or polymer-bound linker. The molecule can be attached before material formation or coupled to a preformed construct. Covalent retention reduces premature diffusion, but the conjugation site must be selected using the structure–activity relationship of the molecule. Modification of an essential pharmacophore can change the solubility, target binding, cellular uptake, or biological activity [105,106].

Non-cleavable tethering produces an immobilized signal rather than a conventional delivery system. It is appropriate only when the molecular target is accessible at the material interface and the active region remains available after the attachment. Molecules that require cellular uptake or intracellular receptor binding generally cannot act while permanently attached. Linker length, orientation, surface density, and steric crowding are therefore important, and biological testing should include the activated polymer without the active molecule as a control.

Cleavable conjugates convert covalent immobilization into a delivery strategy. Hydrolysable bonds can provide time-dependent release, whereas enzyme-cleavable linkers can connect release to cell invasion, inflammation, or matrix remodeling. Redox- or ROS-sensitive bonds respond to the local chemical environment, and photolabile groups allow external timing and spatial control. These categories are not interchangeable: a hydrolysable ester is not necessarily tissue-responsive, extracellular reducing conditions may be insufficient for a disulfide linker, and proof of cleavage with a high oxidant concentration does not establish the in vivo selectivity [106,107].

Classical tissue-engineering studies demonstrate the main principles of cleavable polymer–drug conjugation. Nuttelman et al. attached dexamethasone to a monoacrylated PEG macromer through a hydrolytically degradable lactide-containing ester tether. The conjugate was incorporated into a PEG hydrogel during photopolymerization, and ester hydrolysis released the biologically active dexamethasone that supported the osteogenic differentiation of encapsulated human mesenchymal stem cells [108]. Yang et al. connected dexamethasone to an enzyme-sensitive peptide incorporated into a thiol–ene PEG network, allowing the cell-mediated and spatially restricted release [109]. Broader drug delivery studies have also demonstrated the independent adjustment of linker cleavage and matrix degradation, as well as repeated light-triggered release. Broader drug delivery studies have also demonstrated the repeated light-triggered release of the covalently retained small molecules. Shah et al. developed a photo-triggerable PEG hydrogel–nanoparticle scaffold that enabled repeated on-demand release of camptothecin. However, because the system was evaluated using a cancer-cell model, it should be regarded as a mechanistic proof of principle rather than direct evidence for small-molecule-based tissue engineering [110].

Conjugation efficiency, degree of polymer functionalization, residual free drug, purification, and stability during fabrication and storage should be reported. Release studies must identify the chemical species produced after cleavage because the product may be the parent drug, a linker-containing derivative, or a polymer-bound fragment. Conditions with and without the proposed trigger are required, together with the controls that separate linker cleavage from polymer erosion and passive diffusion. Covalent strategies are justified when strong retention, contact-dependent activity, or a defined trigger provides a clear benefit over simpler physical methods.

### 5.3. Fabrication Platforms and Process-Specific Constraints

The incorporation strategy defines how the molecule is retained, while the fabrication platform defines the processing history and architecture of the final material. The same loading principle can perform differently in a hydrogel, fiber mat, porous scaffold, printed construct, or membrane because each platform imposes different solvents, temperatures, pressures, shear stresses, light exposure, and diffusion distances. Platform selection should therefore consider both tissue function and molecular stability [88,111,112].

For comparative evaluation, the fabrication platforms should be described in terms of both critical process parameters and the critical quality attributes they control. The same nominal formulation may produce a different cargo distribution, chemical integrity, and material performance when processing conditions change. The main relationships are summarized in Table 2 [78,79,84,87,111,112,113,114,115,116,117,118,119,120].

#### 5.3.1. Hydrogels and In Situ-Forming Systems

Hydrogels are water-swollen polymer networks formed by physical or chemical crosslinking. Physical networks are stabilized by reversible ionic, hydrophobic, hydrogen-bonding, crystalline, or host–guest interactions. They can be prepared under mild conditions but may show limited long-term stability. Chemical networks are generally more stable, although the cargo may be exposed to crosslinkers, catalysts, photoinitiators, radicals, or light [79,113].

Transport is controlled by the network mesh size, swelling, degradation, and polymer–molecule affinity. Small molecules that are much smaller than the mesh can leave rapidly unless they bind to the matrix or are placed in a carrier. Increasing polymer concentration or crosslink density may slow diffusion but also change water uptake, mechanics, degradation, nutrient transport, and cell migration. Network geometry and chemical affinity should therefore be optimized together rather than independently.

In situ-forming hydrogels are administered as liquids or weak gels and solidify after injection. Gelation may be triggered by temperature, pH, ions, enzymes, light, or reactions between complementary functional groups. The precursor must be injectable but sufficiently viscous to remain at the defect. Gelation that is too fast may block the needle or create an uneven network, whereas slow gelation may allow dilution, leakage, or early cargo loss. The compound must also remain stable during the trigger and should not inhibit the network formation [81,113].

Hydrogels are particularly suitable for soft tissues, irregular defects, and local delivery under aqueous conditions. Their main constraints are weak mechanics, rapid diffusion of small hydrophilic compounds, dilution before gelation, and possible incompatibility with the crosslinking chemistry. Affinity groups, secondary carriers, degradable linkers, interpenetrating networks, or reinforcement with fibers and porous frames can address these limitations, but each addition increases formulation complexity.

#### 5.3.2. Electrospun Fibrous Materials

Electrospinning produces micro- or nanometer-scale fibers with high surface area and an extracellular-matrix-like architecture. Fiber diameter, alignment, porosity, and surface chemistry can be adjusted through solution and process parameters. The same high surface area and short diffusion distance, however, promote rapid wetting and burst release of surface-associated molecules [84,114,115].

Blend electrospinning is simple but requires compatibility among the polymer, solvent, and small molecule. Rapid solvent evaporation may trap an amorphous dispersion or move the compound toward the fiber surface. Emulsion electrospinning can place a hydrophilic phase within a hydrophobic polymer solution, while coaxial or multiaxial processing creates core–shell or multicompartment fibers. These approaches can reduce surface accumulation and provide a longer transport path, but they require control of phase stability, solvent compatibility, flow-rate ratios, and interface formation.

The process can expose the cargo to organic solvents, strong interfacial and phase-separation effects, and rapid drying. Residual solvent, crystallization, and chemical integrity should be examined after spinning. Secondary carriers may protect the compound but can change viscosity, conductivity, fiber uniformity, and jet stability. Post-spinning adsorption avoids exposure to the spinning solution but usually gives surface-localized loading and weak retention.

Conventional fiber mats can also be thin and densely packed, limiting cell penetration and internal mass transport. Three-dimensional collection, sacrificial fibers, multilayer structures, melt electrospinning, or combination with hydrogels can increase thickness and pore accessibility. These modifications change both mechanics and release and should be evaluated in the complete material system [86].

Accordingly, electrospinning is most suitable for thin or interface-dominated con-structs in which an ECM-like fibrous architecture and local small-molecule delivery are required. It is less suitable for thick constructs requiring extensive cell infiltration or for molecules that are unstable in the required solvent system, unless three-dimensional fiber architectures, hybrid hydrogel systems, or post-fabrication loading are used [84,86,114,115].

#### 5.3.3. Porous Scaffolds Produced by Conventional Methods

Freeze-drying, thermally induced phase separation, solvent casting with particulate leaching, gas foaming, and emulsion templating are widely used to produce porous scaffolds. Their architecture depends on polymer concentration, solvent composition, temperature history, porogen characteristics, gas pressure, emulsion stability, and drying conditions [112,116].

During freeze-drying, the solvent crystals create the pore template. Freezing rate, temperature gradient, sample size, and polymer concentration affects the pore size, orientation, and wall thickness. Solutes are excluded from the growing crystals and may become concentrated in the remaining liquid phase; therefore, the small molecule can migrate, aggregate, or crystallize during freezing and drying. In thermally induced phase separation, the compound must remain stable and appropriately partitioned during solvent exposure, phase separation, and solvent removal.

Particulate leaching provides relatively direct control of pore size through the porogen, but the water-soluble cargo may be lost during leaching. Gas foaming reduces solvent use but may produce closed pores unless combined with another porosity-forming method. Emulsion templating can generate highly porous structures but introduces requirements for surfactant selection, polymerization, removal of the internal phase, and the control of residual monomers or initiators [117].

For all porous scaffolds, increasing porosity generally reduces the load-bearing solid fraction and often decreases mechanical strength. Pore volume, interconnectivity, mechanics, degradation, and cargo transport must therefore be optimized as a connected system. Direct bulk analysis should be complemented by regional measurements when freezing, leaching, or phase separation can create concentration gradients.

Conventional porous-scaffold methods are therefore most useful when interconnected volumetric porosity is a primary design requirement and the cargo can tolerate freezing, phase separation, leaching, or solvent removal. They are less suitable for molecules that readily redistribute, crystallize, or are lost during these operations, in which case post-fabrication loading or carrier-assisted incorporation may provide better control [82,112,116,119].

#### 5.3.4. Additive Manufacturing

Additive manufacturing forms a construct layer by layer from a digital model and offers greater control over external shape, pore geometry, channels, and spatial variation in composition. This makes it possible to design defect-specific constructs and separate small-molecule-containing regions. However, the digital design does not guarantee that the final molecular distribution is retained after mixing, deposition, curing, washing, and storage [65,89,118].

Extrusion printing requires a formulation that flows through the nozzle and recovers sufficient viscosity or crosslinks after deposition. Nozzle diameter, pressure, speed, shear, and gelation rate affect the filament geometry and cargo distribution. In fused filament fabrication, the compound must tolerate both filament production and repeated heating during printing. Thermolabile molecules may instead be loaded after printing or placed in a separately prepared hydrogel phase.

Stereolithography and digital light processing require photocurable groups, a photoinitiator, and defined light exposure. Photosensitive compounds can degrade, while radicals may react with the cargo. Unreacted monomers, photoinitiators, and soluble products must be removed without excessive cargo loss. Inkjet printing permits local deposition but is limited by viscosity, surface tension, particle size, and the small amount delivered per droplet.

Hybrid printing can combine a mechanically strong thermoplastic frame with a drug-loaded hydrogel, coating, fiber layer, or particulate depot. This separates the load-bearing and delivery functions, but introduces interfaces, additional process stages, and new failure modes. Adhesion between components, diffusion across interfaces, regional drug content, sterilization, and reproducibility must be evaluated. Multi-material printing has been used to incorporate two small-molecule signals in distinct phases of a composite scaffold for mandibular bone regeneration [121].

Additive manufacturing is most justified when defect-specific geometry, controlled pore architecture, or regional placement of small molecules is essential. When these spatial advantages are not required, simpler fabrication routes may be preferable because the printing introduces additional rheological, thermal, or photochemical constraints and increases the number of process parameters that must be controlled for reproducible manufacture [87,111,118,121].

#### 5.3.5. Thin and Implantable Material Formats

Films, membranes, patches, and implantable depots are material formats rather than a single fabrication process. They are relevant when regeneration occurs at a tissue surface or interface or when a local reservoir must remain near a defect. These formats may be produced by casting, electrospinning, hydrogel formation, hot-melt processing, coating, multilayer assembly, or combinations of these methods. Their preparation should be assessed through the format-specific requirements, including thickness, water uptake, permeability, adhesion, flexibility, crystallinity, degradation, directional transport, and tissue-contacting surface area [122,123].

Thin constructs provide close tissue contact but also short diffusion pathways. Multilayers, barrier films, affinity sites, and secondary carriers can extend release or produce directional presentation. Hydration may change the dimensions, adhesion, and transport, while poor interlayer adhesion can cause delamination. Implantable depots can maintain local exposure over longer periods but must control the dose, mechanical integrity, degradation products, fibrous encapsulation, sterilization, and possible surgical removal. These formats should be included in tissue engineering only when the polymeric construct contributes to protection, guidance, structural support, or regulation of the regenerative microenvironment.

### 5.4. Programming Stimuli-Responsive and Spatiotemporal Functions

Responsiveness is programmed during material preparation by placing the responsive element at a defined structural level. The element may be an ionizable or thermoresponsive polymer segment, a reversible or degradable crosslinker, a cleavable polymer–drug bond, a responsive secondary carrier, or a functional additive that converts light, ultrasound, electricity, or a magnetic field into a material change. Core–shell, multilayer, and compartmentalized architectures provide an additional level of spatial control [75,124,125].

A responsive system should connect the following three experimentally demonstrated events: the relevant stimulus, a change in the material structure or properties, and a change in the small-molecule presentation. A responsive chemical group alone is not sufficient. The response must occur within the range expected at the target site and must remain distinguishable from passive diffusion, non-specific heating, or material damage.

The performance of a stimuli-responsive system should be described using measurable parameters. Relevant characteristics include the stimulus threshold, response time, magnitude of the change in release relative to the unstimulated material, off-state leakage, reversibility, number of activation cycles, functional fatigue, and spatial resolution. For externally activated systems, the attenuation and penetration of the applied stimulus through the surrounding tissue should also be considered. These parameters provide a more rigorous comparison than the cumulative release profiles alone and help distinguish a useful responsive system from a material that shows only a small or non-specific change under stimulation [75,81,124,125,126,127,128,129,130,131].

#### 5.4.1. Endogenous Stimuli

Endogenous signals include pH, reactive oxygen species, redox conditions, enzymes, and metabolites. Their main advantage is the activation without an external device, but their intensity varies among tissues, patients, and stages of healing. The responsive range should therefore be selected using measured conditions at the intended site rather than a general concept of a diseased microenvironment.

pH-responsive polymers contain acidic or basic groups that change ionization, swelling, or molecular affinity. Alternatively, acid- or base-labile bonds can be placed in a crosslinker, carrier, or polymer–drug conjugate. Small pH differences may be insufficient to generate a useful response, and the release remains dependent on mesh size and polymer–drug interactions [126]. ROS-responsive materials use oxidation-sensitive groups in the backbone, crosslinker, carrier, or linker. Because individual ROS differ in reactivity and concentration, response to a high laboratory concentration of hydrogen peroxide should not be treated as proof of physiological selectivity.

Enzyme-responsive systems use cleavable peptides or other substrates to link release to inflammation, cell invasion, or matrix remodeling. The substrate must remain accessible, and the enzyme concentration should be relevant to the target tissue. Closely related enzymes may reduce specificity, while insufficient activity may delay release. Controls with and without enzyme and, where possible, selective inhibitors are needed [127].

A biological microenvironment often contains several signals at the same time. Multi-responsive systems can improve selectivity by requiring two changes or can amplify a weak signal. However, each responsive component should first be characterized separately so that the contribution of pH, ROS, enzymes, or metabolites can be identified.

#### 5.4.2. Exogenous Stimuli

External stimuli provide greater temporal control because activation can be started, stopped, repeated, or focused. The complete stimulation protocol—the intensity, duration, frequency, geometry, and distance from the material—must be treated as part of the preparation and testing strategy.

Temperature can trigger in situ gelation or change polymer hydration and permeability. These are different functions: gel formation after the injection does not necessarily provide temperature-triggered drug release. Light can activate photocleavable groups, photoisomerization, photochemical crosslinking, or photothermal additives. Ultraviolet light is effective for many chemistries but has limited tissue penetration and can damage cells or compounds; visible and near-infrared systems may improve access but usually require additional functional components [128].

Ultrasound can induce mechanical deformation, cavitation, heating, or increased permeability and is attractive for deeper tissues, but its effects depend strongly on frequency, intensity, and duty cycle [129]. Many electrically activated systems require electrodes or another interface that can deliver a reproducible electric field or potential to the material. Their performance therefore depends on electrode placement, stable electrical contact, tissue conductivity, and charge and mobility of the incorporated molecule [130]. Magnetic activation should also be described according to the applied field. Alternating high-frequency magnetic fields are commonly used to produce local heating, whereas the magnetic-field gradients, rotating fields, or lower-frequency fields can generate force, movement, or deformation. In all cases, particle aggregation may cause non-uniform heating or actuation and may alter the mechanical properties of the polymer matrix [131].

Before stimulation, the material should retain the molecule and preserve activity. After stimulation, the response should be large enough to change the local presentation without causing uncontrolled leakage, structural failure, or tissue damage. Triggered release should be demonstrated against unstimulated control and, where relevant, against controls that reproduce non-specific heating or mechanical stress.

#### 5.4.3. Spatially Patterned, Sequential, and Multi-Agent Systems

Spatial and temporal presentation can also be programmed through architecture. Core–shell fibers, particles, and scaffolds use the shell as a diffusion, degradation, or stimulus-responsive barrier. Multilayer constructs place different molecules or polymers in separate regions. Multi-material printing, patterned photopolymerization, and localized coatings can create discrete depots or concentration gradients [132].

Passive and triggered mechanisms can be combined. Slow background diffusion may maintain a basal concentration, while an external signal produces an additional pulse. Such systems offer flexibility but are more difficult to manufacture and interpret. The simplest architecture that provides the required biological sequence should be preferred.

### 5.5. Post-Processing, Sterilization, Storage, and Critical Quality Attributes

The final properties of a loaded polymeric material are established only after completion of the intended manufacturing workflow, including washing, solvent removal, drying, sterilization or aseptic processing, packaging, and storage. These operations can change both the polymer matrix and the incorporated small molecule and should therefore be considered as part of preparation and quality assessment. Characterization should be performed on the same clinically relevant form that is used in biological studies, including the intended terminal sterilization treatment or the aseptic manufacturing process and, where relevant, the final packaging configuration.

Washing removes the monomers, crosslinkers, catalysts, photoinitiators, surfactants, salts, and weakly retained material. It can also remove a significant part of a physically loaded small molecule. Solvent drying may change polymer-chain mobility and drug crystallization, while freeze-drying creates the pore structure through the freezing and sublimation history. Freezing rate, sample size, polymer concentration, and drying conditions can therefore alter morphology, mechanics, and cargo distribution [119]. Photocrosslinked systems additionally require control of conversion, oxygen inhibition, residual photoinitiator, and the influence of the initiation system on swelling and degradation [120].

Sterilization must be selected for the complete loaded formulation. Steam and dry heat can deform or hydrolyze many polymers and degrade thermolabile compounds. Ethylene oxide operates at a lower temperature but requires control of residual gas and may affect fibers and polymer molecular weight. Gamma and electron-beam irradiation can cause chain scission, crosslinking, oxidation, or changes in crystallinity. Reported compatibility of one polymer or scaffold cannot be transferred directly to another formulation [133,134,135,136].

A suitable terminal sterilization method may not be available when heat, irradiation, or ethylene oxide damage the small molecule, secondary carrier, cleavable linker, coating, or the responsive function. In such cases, aseptic manufacturing from sterile-filtered solutions or separately sterilized components may be required. The feasibility of sterile filtration depends on the component size, viscosity, and possible loss of the molecule through adsorption to the filter. Assembly, loading, and packaging must then be performed under controlled aseptic conditions. Surface exposure to ultraviolet light should not be treated as equivalent to validated sterilization of a thick, porous, or multilayer construct because penetration is limited and shadowed internal regions may remain untreated [133,134].

The physical state of the small molecule is a critical quality attribute. A compound may be molecularly dispersed, amorphous, crystalline, aggregated, or phase-separated. These states differ in stability and release. Electrospinning, hot-melt processing, drying, sterilization, and storage can cause transitions between them. Differential scanning calorimetry and X-ray diffraction provide bulk information, while Raman or infrared mapping and microscopy can reveal local domains that are missed by a single bulk method [85,137,138].

Chemical identity should be evaluated separately from the physical state using validated chromatographic methods selected according to the expected degradation pathways. HPLC with ultraviolet or fluorescence detection may be sufficient for chemically stable compounds with suitable chromophores, whereas LC–MS can be required when oxidation products, hydrolysis products, linker-containing derivatives, or structurally related impurities must be distinguished [106,139]. Extractables and leachables may include polymer additives, unreacted monomers, oligomers, processing aids, and compounds transferred from packaging or manufacturing equipment. Non-targeted chemical analysis may therefore be required for complex implantable systems [139].

Storage studies should use the terminally sterilized or aseptically manufactured product in its final packaging configuration. Temperature, humidity, oxygen, light, and residual water can change the polymer molecular weight, small-molecule potency, molecular mobility, crystallization, and responsive behavior. Accelerated testing is useful for identifying possible failure modes but should not replace real-time studies because the mechanism of change may differ.

Raw-material variability should also be included in the assessment of manufacturing reproducibility. For natural and semisynthetic polymers, relevant parameters may include biological source, molecular weight and molecular-weight distribution, degree of substitution or deacetylation, residual proteins and salts, endotoxin content, and batch-dependent viscosity. For chemically modified polymers, such as methacrylated gelatin or hyaluronic acid derivatives, the degree and distribution of functionalization can influence the crosslinking conversion, swelling, degradation, cargo binding, and mechanical properties. These raw-material attributes should be recorded and linked to the critical quality attributes of the final formulation [78,81,82,113]. Table 3 summarizes the main risks and ways to control them during the post-processing of materials and delivery systems.

For comparative studies, the critical quality attributes should cover the following five connected groups: (i) composition and chemical purity; (ii) actual small-molecule content, chemical integrity, solid state, and spatial distribution; (iii) scaffold dimensions, morphology, porosity, swelling, degradation, and mechanics; (iv) bioburden, endotoxin, and sterility; and (v) preservation of the intended passive or responsive function. Batch-to-batch reproducibility should be evaluated using independently prepared batches and linked to the defined process parameters. A high average loading is not sufficient when the content varies strongly between regions or the batches.

### 5.6. Comparative Assessment and Selection of Preparation Strategies

Strategy selection should start with the required biological exposure and the physicochemical properties of the small molecule. The target tissue defines the necessary architecture, mechanics, implantation route, and treatment period. The molecule then defines compatible solvents, temperatures, reactive conditions, binding interactions, and possible conjugation chemistry. The simplest strategy that provides adequate molecular integrity, retention, and spatial control should be selected. Responsive carriers, multilayers, or cleavable linkers should be added only when passive methods cannot meet the required function.

The comparisons in Table 4 should not be interpreted as fixed numerical rankings. Attainable loading depends on the definition and denominator used, as well as on the molecule, polymer, carrier fraction, material geometry, and analytical method. High nominal loading is not necessarily advantageous when the compound is chemically degraded, phase-separated, strongly bound but unavailable, concentrated only near a surface, or released outside the biologically useful period. Comparisons should therefore be based on the amount of intact compound in the final sterilized or aseptically manufactured material.

Dose and scale should be normalized according to the intended application. Drug content per dry material mass is useful for formulation comparison, but the dose per implant volume or tissue-contacting area may be more relevant for the local biological exposure. Material dimensions should be reported because the diffusion distance, surface-area-to-volume ratio, and pore accessibility change during the scale-up. Regional content measurements are required when the process can produce the gradients across thick, multilayer, coated, or printed constructs [82,83].

Strategy selection should follow a defined sequence. First, the target tissue and implantation route should establish the required architecture, mechanical function, degradation period, and treatment duration. Second, the physicochemical properties and processing stability of the molecule should be matched to compatible loading routes. Third, the simplest strategy that provides sufficient intact-drug content, spatial distribution, retention, and biological availability should be selected. Responsive carriers, multilayers, or cleavable conjugates should be introduced only when the simpler passive approaches cannot provide the required temporal or spatial presentation. Finally, the complete material should be evaluated after post-processing, sterilization or aseptic manufacture, packaging, and storage using independently produced batches. Clinical translation is more likely for the simplest reproducible system that preserves molecular integrity, material function, and the required spatiotemporal presentation [82,140].

## 6. Pre-Clinical Investigations on the In Vitro and In Vivo Models

### 6.1. Current Scope and Principles of Preclinical Evaluation

Preclinical investigation of a smart polymeric material begins with the complete construct rather than with the polymer and incorporated molecule considered separately. Molecular loading can influence the network formation, mechanical behavior and degradation, while the matrix determines the cargo retention, temporal availability and exposure to the surrounding tissue [141,142]. In a hydrogen-bonded cartilage hydrogel, for example, tannic acid contributed to the network reinforcement and early control of oxidative and inflammatory conditions, while the same construct provided the delayed kartogenin release to support cell recruitment and chondrogenic differentiation [143]. A related interdependence was observed in a polyimidazolium hydrogel containing N-acetylcysteine: antibacterial activity originated primarily from the polymer network. The incorporated molecule supplied antioxidant and differentiation-promoting functions required for the tissue repair [144]. Such results indicate that the preclinical performance emerges from interactions among material structure, cargo activity and release behavior, not from the simple addition of their isolated properties [145,146].

Physicochemical characterization acquires biological meaning only when it is connected to the proposed therapeutic mechanism [142]. Loading efficiency and cumulative release describe the amount of cargo present and liberated, but neither parameter confirms that the molecule remains active after incorporation. Yang et al. related the sequential release of tannic acid and kartogenin to distinct biological phases: initial attenuation of oxidative stress and inflammation followed by endogenous cell recruitment and cartilage formation, instead of treating release kinetics as an independent material property [143]. Pranantyo et al. verified N-acetylcysteine activity through antioxidant assays and keratinocyte differentiation in a three-dimensional human skin equivalent before examining the repair of infected diabetic wounds [144]. In a redox-active periodontal hydrogel, sustained H_2_S delivery was likewise linked to mesenchymal stem-cell recruitment, angiogenesis and osteogenesis instead of being inferred solely from the donor release [147]. Thus, the relevant endpoint is preservation of the intended pharmacological function under the conditions created by the final material, while it is important to exclude the separate testing of the free compound.

Collectively, the preclinical evaluation is intended to establish that the final construct retains the intended physicochemical organization, responds to the relevant biological trigger, preserves the activity of the incorporated molecule and produces a tissue-level benefit through the proposed mechanism, not merely through nonspecific material effects. Taken together, the preclinical evaluation is a continuous chain of evidence linking material composition, molecular activity, release or activation, cellular mechanism and tissue-level outcome [142].

### 6.2. Verification of the Polymeric Construct

The material subjected to biological testing is more appropriately defined by its composition after fabrication rather than by the nominal formulation of its precursors [146]. Because crosslinking, solvent removal, washing, swelling and other processing steps may alter both the polymer network and the incorporated molecule, the evidence obtained for the isolated components cannot substitute for the characterization of the final construct [145]. The required analytical strategy depends on how the active compound is integrated. Physically entrapped molecules must be distinguished from the species retained through non-covalent interactions, covalently immobilized groups and bioactive products generated during the network degradation. These states are not interchangeable because they determine whether the molecule remains freely diffusible, contributes to network formation or becomes available only after cleavage of the material [145,148]. In a polyimidazolium-alginate dressing, for example, N-acetylcysteine was incorporated as an intrinsic, low-leaching component; the authors quantified thiol functionalization and examined the release of individual network constituents from the completed fibers [144]. Conversely, TCDI crosslinking of decellularized heart valves produced cleavable thiourea and thiocarbamate bonds that formed part of the supporting network and generated H_2_S during degradation. Verification of this system required evidence of the linkage chemistry, double-network formation, fatigue resistance and H_2_S-generating degradation, making a conventional drug-loading assay insufficient [148,149].

The identification of the active compound in the precursor solution does not establish its amount or chemical state in the final material. The nominal input, the fraction retained after processing and the fraction subsequently recoverable or released under the defined conditions represent the separate quantities [145,150]. This distinction becomes particularly important when the low cargo concentrations preclude direct detection by bulk spectroscopy or when the matrix components interfere with the quantification. In the tannic-acid- and kartogenin-containing polyurethane hydrogel reported by Yang et al., FTIR supported the hydrogen-bond formation between tannic acid and the polymer, but the characteristic kartogenin peaks could not be resolved because of its low concentration. The two compounds were quantified during release by different analytical methods—UV spectroscopy for tannic acid and HPLC for kartogenin—illustrating that the structural characterization of the network and quantitative analysis of its molecular cargo provide the complementary, rather than equivalent, information [143]. A spectral feature assigned to a functional group may confirm a chemical interaction, but it does not by itself establish the molecular recovery, purity or the absence of processing-induced transformation [151]. Direct quantification requires a method with demonstrated selectivity for the active compound in the presence of the polymer, residual reagents and the relevant degradation products.

Total loading is also insufficient when the function depends on localization within a defined phase or compartment. This applies particularly to multilayered, core–shell, and carrier-containing constructs, in which the loss of compartmentalization may change the local concentration, transport distance and interfacial properties without altering the overall molecular content [145,151]. Fang et al. incorporated NaHS into the bovine serum albumin nanoparticles before dispersing this H_2_S-generating subsystem, together with the conductive PEDOT-coated silk microfibers, within a dual-crosslinked alginate/gelatin hydrogel. The final construct comprised chemically and structurally distinct functional phases whose integration could not be inferred from the characterization of the hydrogel matrix alone [147]. In such hierarchical materials, the relevant question is not merely whether every component is present, but whether each remains in the intended domain after fabrication. Spatially resolved analysis is thus warranted when compartmentalization is part of the proposed mechanism. Routine mapping of every homogeneous formulation would add little mechanistic value.

The incorporation of a small molecule may also modify the material independently of its pharmacological activity [146]. Changes in the intermolecular bonding, crosslink density, crystallinity or hydration can alter the mechanics, swelling, adhesion and the degradation, introducing material-related effects that may later be mistaken for the direct molecular bioactivity [141,151]. In the polyurethane system developed for cartilage repair, tannic acid formed additional hydrogen bonds with the polymer, reduced pore dimensions, increased fracture strength and markedly improved resistance to cyclic loading. Kartogenin, by contrast, produced no detectable change in the microstructure, tensile properties or adhesion under the conditions tested [143]. The comparison among unloaded, single-component and dual-loaded formulations was essential for allowing the structural contribution to tannic acid to be distinguished from that of kartogenin. A related principle is evident in the PolyLA-Na/PolyLA oral patch, where hydrogen bonding between the two lipoic-acid-derived polymers suppressed PolyLA depolymerization, reduced PolyLA-Na crystallinity and produced extensible, water-responsive material capable of sustained generation of LA-based small molecules [152]. Here, the active molecular species and the structural polymers shared the same chemical origin, making the network composition, polymerization state and wet-state performance inseparable elements of the construct verification.

Based on these considerations, the verification of the final test article encompasses at least four system-dependent attributes as follows: chemical identity, actual molecular content, localization where compartmentalization is functionally relevant, and the structural consequences of the incorporation. Mechanistic interpretation generally requires an identically processed unloaded matrix and, in multicomponent systems, formulations omitting individual functional constituents where technically feasible. Such comparisons help to determine whether the subsequent release or biological behavior arises from the intended molecular activity, altered material properties or both. Only after this baseline has been defined can stimulus-dependent transformation and release kinetics be interpreted without ambiguity [146].

These characterization steps also define the boundaries within which subsequent biological models can be interpreted. Simple cellular systems are advantageous at this stage because they provide reproducible conditions for assessing cytocompatibility, concentration-dependent responses and direct cellular effects of the complete construct. However, such models do not reproduce tissue architecture, spatially heterogeneous molecular exposure or multicellular interactions that may determine the performance of a responsive material in vivo. Their primary value is in establishing an initial biological baseline and identifying overt material- or molecule-related effects, rather than in predicting tissue-level therapeutic performance.

### 6.3. In Vitro Stimulus Responsiveness and Molecular Availability

In vitro release experiments need to distinguish stimulus-responsive behavior from passive sustained delivery. A prolonged or sequential profile may arise from molecular solubility, partitioning within the polymer, non-covalent affinity, diffusion length or gradual matrix erosion without any stimulus-dependent transition. In the tannic acid- and kartogenin-containing polyurethane hydrogel developed for cartilage regeneration, tannic acid was released predominantly during the first week, whereas kartogenin remained associated with the construct for several weeks. This temporal separation was attributed to differences in hydrophilicity, interactions with the network and dependence on polymer degradation rather than to activation by a pathological cue [143]. The system demonstrates programmed temporal delivery, but it also illustrates why a multistage release curve does not, by itself, constitute evidence of responsiveness. A smart-material response is established only when the variation in a defined stimulus produces a reproducible change in the material and in the liberation of the incorporated molecule under otherwise comparable conditions.

The distinction becomes particularly important in the constructs described as microenvironment responsive. A convincing in vitro design compares the same formulation under a basal condition and across a physiologically justified range of the proposed trigger. Binary comparisons between the absence and presence of an arbitrarily selected stimulus establish chemical susceptibility, but provide little information on the activation threshold, dynamic range or selectivity. In the ROS-sensitive core–shell microneedles, the boronate-containing crosslinks in the polyvinyl alcohol shell were designed to degrade in an oxidative environment, thereby exposing the heparin-based core and releasing verteporfin as the wound entered later healing phases. The authors examined peroxide-dependent shell degradation and molecular release, linking the chemical sensitivity of the crosslinker to a change in the accessibility of distinct structural compartments [153]. This concentration-dependent design is more informative than testing a single oxidative condition because it begins to define whether the construct responds gradually, exhibits a threshold or becomes rapidly exhausted above a certain stimulus level.

Electrospun systems further illustrate why release kinetics become mechanistically informative only when interpreted together with the transport pathway within the construct and not in isolation. The same incorporated molecule may exhibit markedly different release profiles depending on whether it is distributed throughout a homogeneous fiber, concentrated near the fiber surface or confined within a core–shell architecture. These configurations modify diffusion distance, polymer–molecule interactions and relative contributions of diffusion and matrix degradation without necessarily involving the stimulus-dependent activation. Tipduangta et al. demonstrated that the phase separation occurring during electrospinning altered the internal fiber microstructure and consequently the release behavior of the incorporated drug despite comparable precursor compositions [154,155]. Molecularly imprinted electrospun fibers containing ferulic acid or khellin showed that the specific polymer–molecule interactions slowed the release of one compound but not the other, indicating that the retention mechanisms cannot be generalized across different cargos even within the same fibrous platform. Accordingly, changes in the release rate do not constitute evidence of responsiveness unless they are accompanied by independent confirmation of the proposed activation mechanism and distinguished from the architecture-dependent diffusion or affinity effects.

Physiological relevance cannot be inferred from the chemical identity of the trigger alone. Hydrogen peroxide concentrations, pH values, enzyme activities, glucose levels and temperatures used in vitro are most informative when they reflect the magnitude and duration expected at the intended implantation site. Excessively strong conditions may demonstrate the maximal degradability while bypassing the rate-limiting processes that govern activation in the tissue. For example, the glucose/ROS-responsive AAT-ZCG hydrogel developed for the diabetic bone defects relied on the dynamic borate ester chemistry and an enzyme–nanozyme cascade to couple hyperglycemia, oxidative stress and local acidification with release of tannic acid, functional nanoparticles and, subsequently, zoledronic acid [156]. The study establishes that the material can react to the chemical features associated with the diabetic microenvironment; however, the translation of such responsiveness requires more than exposure to one high concentration of glucose or peroxide: the relevant question is whether the response persists across heterogeneous and time-dependent ranges encountered during the bone repair. A trigger concentration selected solely because it produces the rapid degradation may overestimate the in vivo activation, whereas an overly mild condition may fail to reveal the operational range of the material.

The claimed responsiveness must also be demonstrated as a biologically consequential process. Changes in swelling, network integrity or molecular release in a simplified buffer establish the physicochemical sensitivity, but do not prove that the same response will occur at a sufficient magnitude, duration or spatial range in diseased tissue [142,157]. This issue becomes particularly important when activation depends on a pathological microenvironment. Fang et al. evaluated their redox-active H_2_S-releasing hydrogel in diabetic periodontitis, where hyperglycemia, oxidative stress and persistent inflammation were integral to the therapeutic rationale [147]. Cheng et al. developed a double-layer dressing in which the gradual reaction of the polymer with the reactive oxygen species both reduced the oxidative stress and generated succinic acid as a regenerative degradation product; its performance was confirmed in the diabetic mouse and porcine wounds extending the evidence beyond cell-free oxidative conditions [158]. For mechanically active cartilage, Yang et al. combined cyclic loading, body-temperature-induced shape recovery, molecular release and defect repair because these functions were expected to operate simultaneously after implantation [143]. Responsiveness is biologically substantiated when the relevant trigger modifies material behavior and the resulting change can be causally linked to a predefined cellular or tissue-level effect.

For this reason, the quantitative analysis of stimulus–release relationships are essential; several stimulus levels are required to determine baseline leakage, onset of accelerated release, the steepness of the response and the approach to saturation. Repeated or reversible systems additionally require cyclic testing to establish whether the response can be reproduced after the first activation. Ideally, the experimental readout includes both the absolute amount released and the fraction of the verified molecular content remaining in the construct. Reporting only cumulative percentages may not display the substantial differences in loading or incomplete molecular recovery. Also, the mechanistic origin of the altered release profile must be demonstrated independently. A faster increase in the concentration of cargo in the surrounding medium may result from cleavage of a responsive bond, increased swelling, loss of crosslink density, bulk disintegration, desorption, dissolution of a secondary carrier or conversion of a bound precursor into a diffusible product. Bond cleavage may preserve the macroscopic construct while increasing the mesh size, bulk erosion reduces both material integrity and residual dose, liberation of an embedded nanoparticle introduces a second transport step, and degradation-generated molecules may not exist in their active form before the network cleavage. Release measurements gain mechanistic value when paired with a physicochemical readout that directly reflects the proposed transformation, such as responsive-bond cleavage, changes in swelling, rheology, mass loss, pore structure or carrier integrity.

This mechanistic pairing is particularly important in hierarchical materials. In the programmed microneedle system, ROS did not merely accelerate the diffusion through an otherwise unchanged polymer. Oxidative cleavage altered the shell architecture and exposed an internal functional compartment [153]. In the AAT-ZCG hydrogel, the initial glucose- and ROS-dependent response was coupled to further acidification and decomposition of the zoledronic acid–cerium complex, creating a cascade in which the matrix response, nanoparticle transformation and molecular release occurred at different levels [156]. A single cumulative curve would not resolve these individual steps. Such systems require separate measurements of the matrix degradation, carrier liberation and final small-molecule availability if the proposed cascade is to be regarded as experimentally demonstrated directly instead of inferred from biological efficacy.

Layer-by-layer assemblies further illustrate that the release kinetics are most informative when interpreted together with the structural mechanisms governing the molecular transport through the construct. In multilayer systems, delayed or sequential release may arise from interlayer diffusion, diffusional barrier layers or progressive disassembly of the individual compartments without involvement of stimulus-dependent activation. Howard et al. modified the self-assembled multilayer films by introducing laponite barrier layers between the BMP-2-containing compartments, extending the release from approximately 2 to 30 days while maintaining the same incorporated dose [159]. The study demonstrated that release behavior was governed primarily by molecular redistribution during film assembly and by the diffusion across the multilayer architecture instead of the amount of incorporated cargo, and that the prolonged release profile produced superior bone regeneration compared with the rapid release of the same growth factor. These findings emphasize that the cumulative release curves alone cannot distinguish the architecture-controlled transport from genuine material responsiveness and become mechanistically informative only when interpreted together with physicochemical evidence of multilayer organization throughout the release process. A similar principle applies to the externally activated multilayer coatings, in which the electrical stimulation or other external cues trigger the release only after controlled structural transformation of the film, reinforcing need to be related to the underlying activation mechanism instead of the release kinetics alone.

External triggers require an additional assessment of controllability. Heat-, light- or ultrasound-responsive materials can offer temporal precision, but the in vitro experiment must define the exposure required to activate the release, the background leakage between the activations and the stability of the construct during the repeated stimulation. An all-small-molecule dynamic covalent hydrogel formed from tobramycin, tannic acid and a formylphenylboronic-acid linker showed the heat-triggered liberation of its antibacterial constituents through disruption of dynamic covalent interactions [160]. Because the therapeutic molecules also contributed to the network formation, molecular release was inseparable from the partial network dissociation. Drug release after thermal activation is only one aspect of the construct performance. Equally important is whether the activation protocol is safe for the surrounding tissue and whether the repeated stimulation leaves the construct capable of continued therapeutic delivery.

Pulsatile delivery still imposes stricter requirements. A photo-induced imine-crosslinked wound dressing containing biodegradable microcapsules was designed to release a TGF-β inhibitor at a selected stage of healing instead of continuous release from implantation onward [161]. For such platforms, a conventional cumulative release profile obscures the principal function. The critical parameters are background release before activation, the dose liberated by each pulse, temporal reproducibility and retention of the remaining cargo between pulses. Demonstration of a single light-induced increase is insufficient to establish programmable delivery unless subsequent activations remain effective and uncontrolled leakage remains low.

The interpretation of burst, sustained and sequential release is meaningful only when tied to the intended biological sequence and should not be viewed as an intrinsic quality criterion. Rapid exposure may be advantageous when an early antimicrobial, antioxidant or anti-inflammatory effect is required, but detrimental when the same molecule must remain locally available throughout matrix deposition or tissue remodeling. Conversely, delayed delivery is useful only if the active compound remains stable and reaches an effective concentration at the relevant stage. The tannic acid–kartogenin hydrogel illustrates this principle: early tannic acid release was aligned with the modulation of the inflammatory environment, whereas the prolonged kartogenin delivery supported later chondrogenic processes [143]. Nevertheless, different release rates do not by themselves prove that the chosen temporal order is necessary: stronger evidence would compare the programmed formulation with systems in which the timing is altered while the total dose and composition are retained.

Release media are best selected according to the mechanism under investigation. Phosphate-buffered saline provides a reproducible baseline but cannot reproduce the oxidative, enzymatic, protein-rich or mechanically loaded tissue environments. Mechanistic testing is most informative when performed sequentially: first under the simplified conditions that isolate the proposed trigger, then across a dose range, and finally in a biologically enriched medium that introduces the relevant competing reactions. This progression distinguishes genuine stimulus sensitivity from the effects caused by non-specific ionic strength, protein adsorption or accelerated matrix degradation.

Chemical detection of a released compound does not establish the preservation of its biological function. Processing, prolonged residence within the polymer and the stimulus-induced cleavage may alter the molecular structure or generate inactive and potentially harmful products, so the collected release fractions are ideally analyzed for chemical integrity and, where feasible, tested in a mechanism-relevant assay against an equivalent concentration of free compound. Antibacterial activity, antioxidant capacity, pathway inhibition or induction of a regenerative cell phenotype can provide this functional link, provided that the assay separates the contribution of the released molecule from that of soluble polymer fragments. In vitro responsiveness is demonstrated not by release under a single nominal trigger condition, but by a quantitative and mechanistically resolved relationship between a biologically relevant stimulus, transformation of the polymeric construct and liberation of an intact, active small molecule. Establishing this relationship provides the necessary basis for subsequent cellular experiments, in which the material response must be connected to changes in cell behavior rather than inferred from release kinetics alone.

Finally, the interpretation of stimulus-responsive behavior is also dependent on the experimental model in which molecular availability is assessed. Acellular release systems provide the highest degree of control over the stimulus concentration, exposure time and material composition and are consequently appropriate for establishing the intrinsic responsiveness of the construct. Their limitation is that they do not account for cellular uptake, extracellular-matrix interactions, enzymatic activity or other biological processes that may modify the molecular availability. Cellular models can address some of these factors but remain limited in reproducing tissue-scale transport and spatial heterogeneity. Also, acellular and cellular release studies should be regarded as complementary rather than interchangeable approaches, with the choice of model determined by whether the primary uncertainty concerns material responsiveness itself or the biological availability of the released molecule.

### 6.4. Mechanistic Validation in Advanced In Vitro Models

In vitro models provide the first opportunity to establish this mechanistic link. Conventional viability assays remain necessary for excluding overt toxicity, but they are insufficient for a material designed to regulate inflammation, infection, oxidative stress or tissue-specific differentiation. Such claims require the mechanism-linked cellular endpoints and experimental conditions that reproduce the relevant biological challenge [146,162]. N-acetylcysteine-containing hydrogels were examined through antibiofilm activity, mammalian-cell compatibility and keratinocyte differentiation, thereby addressing both microbial control and restoration of epithelial function [144]. The H_2_S-releasing periodontal system was assessed through oxidative and inflammatory regulation, stem-cell recruitment, angiogenic responses and osteogenic differentiation, reflecting interacting processes required for the periodontal reconstruction [147]. Kartogenin-containing hydrogels were evaluated through chondrogenic differentiation, cartilage-matrix formation and mitochondrial regulation, consistent with the proposed action of the molecule in cartilage repair [143]. The choice of cellular endpoints must follow a defined mechanism of action and the biological requirements of the target tissue [162,163].

A persistent limitation in the preclinical evaluation of smart polymeric materials is the gap between reductionist cell-based assays and in vivo implantation. Responsive constructs are commonly characterized through acellular release studies and conventional mono- or co-cultures before direct progression to animal models. This sequence can confirm cytocompatibility, pathway activation and regenerative potential, but it provides limited information on whether the designed polymer–molecule interaction remains operative under the tissue-scale transport constraints. In vivo, material activation is governed by spatially heterogeneous concentrations of reactive oxygen species, enzymes, metabolites or inflammatory mediators, while the biologically available dose of the incorporated molecule is further shaped by the diffusion through the extracellular matrix, convective removal, protein binding, cellular uptake and degradation. These variables are integral to the function of smart biomaterials, which both regulate and respond to the surrounding cells rather than acting as inert delivery reservoirs [145,146].

The selection of an advanced in vitro model is best guided by the uncertainty that remains after the acellular and conventional cellular testing. Three-dimensional cultures are relevant when diffusion through the extracellular matrix or spatially restricted activation may limit the molecular availability. Multicellular models are required when the therapeutic activity depends on interactions among immune, stromal, endothelial or tissue-specific cells. Perfused systems are particularly informative when convective removal, repeated exposure to the activating stimulus or transport across a tissue barrier may determine the construct performance. Ex vivo tissues retain the native extracellular-matrix organization and tissue interfaces and can support the short-term assessment of penetration, retention and local material–tissue interactions. These models are most useful when selected according to the proposed mechanism and anticipated mode of failure instead of serving as progressively more sophisticated substitutes for conventional culture.

Three-dimensional multicellular cultures, organoids, perfused microphysiological systems and ex vivo tissues could address selected uncertainties at this intermediate stage, including stimulus penetration, local retention, barrier crossing, repeated activation, washout and the multicellular feedback. Their value, however, depends on a clearly defined context of use rather than on the model complexity itself. An advanced model is more informative when it reproduces the particular tissue variable expected to determine material performance or failure. Adding cell types or architectural features that are unrelated to the responsive mechanism does not necessarily improve the predictive validity [163,164]. Organoids and organ-on-chip platforms can reproduce selected features of the tissue organization, dynamic flow and compartmental communication, but their matrices, device materials and culture conditions may themselves alter the stimulus availability or sequester released small molecules and require the material-specific controls [165,166]. At present, these approaches are not routinely incorporated into the validation pipeline for responsive polymeric systems containing small molecules. Their most defensible role is not to replace animal studies, but to resolve defined mechanistic and transport-related uncertainties before in vivo testing and to provide a more rational basis for the selection of material composition, dose, activation range and experimental endpoint.

The increasing complexity of these models should not be interpreted as a linear increase in the predictive validity. Each model provides greater relevance only when the additional biological feature it reproduces, such as three-dimensional diffusion, multicellular signaling, dynamic transport or native tissue organization, is directly implicated in the proposed mechanism of action or represents a plausible source of translational failure.

### 6.5. In Vivo Evaluation in Regenerative and Disease-Relevant Models

The transition from advanced in vitro models to in vivo implantation represents a fundamental shift in the evaluation of the stimulus-responsive biomaterials. Under controlled experimental conditions, activating cues are deliberately introduced, their concentration and duration are predefined, and their accessibility to the material is experimentally controlled. Following implantation, however, material activation becomes entirely dependent on endogenous pathological signals generated within the surrounding tissue microenvironment. Unlike conventional drug delivery platforms, whose therapeutic performance is largely determined by the predetermined release characteristics, smart biomaterials rely on the continuous reciprocal interactions with the surrounding cells and tissues, allowing the material behavior to adapt dynamically to the local biological conditions. This capacity to both regulate and respond to the biological environment has become one of the defining characteristics of contemporary smart biomaterials [142,146].

The endogenous signals responsible for the material activation originate directly from the pathological processes rather than experimental intervention. Reactive oxygen species, inflammatory mediators, proteolytic enzymes, pH alterations, bacterial metabolites and other disease-associated biochemical cues are widely exploited as physiological triggers for responsive biomaterials designed to release therapeutic molecules only under pathological conditions [157]. Consequently, successful activation after implantation depends not only on the polymer architecture but also on whether these pathological signals are generated, maintained and spatially localized within the target tissue.

Immediately after implantation, the material enters a dynamic biological environment shaped by the foreign body response. Adsorption of plasma proteins onto the implant surface is rapidly followed by the recruitment of neutrophils, monocytes and macrophages, formation of foreign body giant cells, extracellular matrix remodeling and, when the implantation persists, the development of a collagen-rich fibrotic capsule surrounding the biomaterial [167]. These sequential events continuously modify the molecular composition and structural organization of the material–tissue interface, thereby altering the biological context in which the stimulus-responsive activation occurs. Contemporary perspectives on biomaterial implantation describe the host response not simply as a determinant of biocompatibility, but as a dynamic process capable of influencing long-term implant performance while simultaneously being modulated by the material properties [167,168]. Conventional and stimulus-responsive delivery systems are affected differently by these processes. In sustained-release platforms, the implantation primarily influences the local distribution and persistence of the released therapeutic agent. By contrast, in responsive biomaterials, pathological signals become integral components of the therapeutic mechanism itself because they directly regulate material activation. The functional behavior of the implanted construct emerges from the interaction between material design and the evolving pathological microenvironment and cannot be explained by the polymer composition alone [142,146]. As tissue repair progresses, the same biological processes that drive the resolution of disease simultaneously reshape the biochemical environment responsible for the activation of the material, making the stimulus-responsive behavior inseparable from the host–material interactions.

These principles also determine the selection of appropriate in vivo models. Because stimulus-responsive biomaterials are designed to react to the disease-associated biochemical signals, the experimental model depends on reproducing not only the anatomical site of implantation but also the pathological microenvironment responsible for the material activation. Animal models that adequately evaluate biocompatibility may provide limited mechanistic information if the relevant inflammatory, oxidative, enzymatic or infectious stimuli are absent or poorly represented. The translational value of in vivo studies depends on matching the biological mechanism of the responsive material to the pathological characteristics of the experimental model beyond anatomical similarity alone [169].

From this perspective, the demonstration of tissue regeneration alone cannot establish that a stimulus-responsive biomaterial has functioned as intended after implantation. Improvement in tissue morphology or restoration of function represents the cumulative outcome of numerous biological processes and may result from the constitutive release, material degradation or intrinsic pharmacological activity of the incorporated small molecule independently of the stimulus-responsive activation. The primary objective of in vivo evaluation is not simply to determine whether regeneration occurs, but to establish that the pathological signals remain functionally coupled to the material behavior under physiological conditions. Mechanistic validation requires evidence that the disease-associated biochemical cues regulate the material activation, that this activation governs the local therapeutic availability of the incorporated molecule, and that the preservation of this regulatory sequence directly contributes to the subsequent molecular, cellular and regenerative responses. Only by demonstrating this causal chain can stimulus-responsive biomaterials be distinguished from conventional sustained-release systems, providing the mechanistic foundation for in vivo studies discussed in the following section.

Demonstrating the therapeutic efficacy after implantation is not sufficient to validate a stimulus-responsive biomaterial. A reduction in inflammation or accelerated tissue repair may result from the intrinsic bioactivity of the incorporated small molecule even if the material no longer responds to the intended pathological stimulus. The central objective of the in vivo evaluation is therefore to establish that the disease-associated signals remain functionally coupled to the material activation under physiological conditions, where the transport limitations, protein adsorption, cellular uptake and continuous tissue remodeling simultaneously influence the availability of both the activating cue and the released therapeutic agent. Mechanistic validation requires evidence that the pathological microenvironment regulates the material behavior and that this regulation directly drives the disease-relevant cellular and molecular events underlying tissue repair rather than simply accompanying regeneration [143,170].

Recent studies illustrate that the same biological objective can be achieved through different responsive mechanisms while addressing the same biological question. In a ROS-responsive hyaluronic acid hydrogel incorporating curcumin liposomes and silver nanoparticles, the elevated oxidative stress within diabetic wounds initiated the material activation, resulting in the coordinated antioxidant, antibacterial and anti-inflammatory effects together with enhanced angiogenesis and tissue regeneration, linking the ROS-rich pathology to the controlled local therapeutic activity through the responsive matrix [170]. A conceptually different strategy was implemented in a multiple hydrogen-bond hydrogel for cartilage regeneration, where the matrix provided stage-dependent release of tannic acid and kartogenin. Instead of relying on the immediate cargo release, the material coordinated the sequential availability of tannic acid and kartogenin with distinct phases of cartilage repair, illustrating that temporal regulation can provide mechanistic control even in the absence of a single dominant pathological trigger [143]. In diabetic periodontal defects, a polyphenol-mediated redox-active hydrogel combined sustained H_2_S delivery with conductive silk microfibers to exploit the gaseous-bioelectric coupling within the pathological microenvironment. Here, bone regeneration resulted from continuous interactions between redox regulation, endogenous bioelectric signaling, immune modulation and bone repair, demonstrating that responsive biomaterials may integrate multiple disease-dependent mechanisms instead of depending on a single activation pathway.

Importantly, the mechanistic validation is not restricted to hydrogel platforms. ROS-responsive selenium-containing electrospun polyurethane nanofibers loaded with deferoxamine, indomethacin and gold nanorods likewise exploited the pathological oxidative stress to regulate the local therapeutic activity, leading to reduced inflammation, enhanced angiogenesis, and improved wound healing. The successful translation of stimulus-responsive behavior from injectable hydrogels to fibrous membranes indicates that the preservation of functional coupling between the pathological signals and the material activation, more than biomaterial architecture itself, governs the performance of smart therapeutic systems in vivo [171].

The choice of the in vivo model should similarly be matched to the specific translational question addressed by the study. Small-animal models offer advantages in experimental accessibility, controlled comparison of formulations and initial assessment of efficacy, biocompatibility and mechanism, but may inadequately reproduce the anatomy, tissue dimensions, mechanical environment or clinically relevant delivery conditions of the target application. Larger-animal models can provide greater anatomical and biomechanical relevance when these parameters are critical to material performance, although they require substantially greater resources and may still differ from humans in immune, metabolic and tissue-specific responses. Thus, the animal model selection should be justified by the physiological variable that the model is intended to reproduce, rather than by the model size or complexity alone.

### 6.6. Experimental Strategies for Mechanistic In Vivo Validation

Recent reviews increasingly recognize that the principal challenge in the preclinical evaluation of stimulus-responsive biomaterials is not demonstrating the therapeutic efficacy itself but establishing that the endogenous pathological signals continue to regulate the material behavior after implantation. Unlike simplified in vitro systems, the in vivo microenvironment is continuously reshaped by inflammation, tissue remodeling and dynamic changes in the concentration of the activating stimuli, making preservation of the stimulus responsiveness considerably more difficult to verify. Thus, mechanistic validation requires independent evidence that the disease-associated cues remain functionally coupled to the material activation under physiological conditions instead of inferring responsiveness solely from improved tissue repair [172].

One approach is to examine whether material activation changes in parallel with the intensity of the pathological stimulus. Joshi et al. addressed this question using an enzyme-responsive triglycerol monostearate (TG-18) hydrogel loaded with triamcinolone acetonide. Drug release accelerated in rheumatoid arthritis synovial fluid and was markedly reduced by matrix metalloproteinase inhibition, directly linking the hydrogel activation to the disease-associated enzymatic activity [173]. The authors then modulated the inflammatory severity in vivo using different doses of arthritogenic K/BxN serum and monitored the hydrogel disassembly through the release of an encapsulated fluorescent tracer. Material degradation increased progressively with the disease severity, demonstrating that the implanted construct dynamically responded to changes in the pathological microenvironment instead of simply following predetermined degradation kinetics. The verification of the material activation, however, represents only one component of the mechanistic pathway. In the TG-18 study, the hydrogel disassembly was inferred from the release of a fluorescent probe, whereas the local pharmacokinetics of triamcinolone acetonide were not quantified directly. The comparison with free drug and blank hydrogel confirmed that the therapeutic efficacy depended on incorporation into the responsive matrix, yet the absence of a compositionally matched nonresponsive hydrogel limited direct discrimination between stimulus-responsive release and prolonged local drug retention. These observations highlight that the material transformation, local therapeutic availability and biological response constitute distinct mechanistic events that are best evaluated independently before the causal relationships between responsiveness and therapeutic efficacy are inferred.

An equally important, yet less frequently discussed, aspect of mechanistic validation is the selection of appropriate experimental controls. Recent reviews emphasize that the therapeutic superiority of a responsive biomaterial cannot, by itself, be interpreted as evidence of stimulus-responsive regulation, because similar outcomes may arise from sustained local drug retention, altered pharmacokinetics, or intrinsic biological activity of the incorporated molecule without any contribution from the pathological stimulus-dependent activation [172]. Robust experimental design requires the controls that interrogate different steps of the causal pathway. The comparison with the free therapeutic molecule distinguishes the contribution of the carrier from that of the drug itself, while blank scaffolds determine whether the biomaterial exerts independent biological effects. Equally important, the comparison with a compositionally matched nonresponsive material enables the direct assessment of the added value of the stimulus-responsive regulation over conventional sustained-release systems. Although complete implementation of this control strategy remains relatively uncommon, combining complementary controls substantially strengthens the causal interpretation of the in vivo findings by separating material activation from downstream therapeutic efficacy.

A complementary strategy focuses on the validation of the biological consequences of responsive activation across multiple levels of tissue organization. Liu et al. developed a ROS-responsive hydrogel based on the dynamic phenylboronate ester crosslinks between aminophenylboronic acid-modified hyaluronic acid and poly(vinyl alcohol) for co-delivery of chondroitin sulfate and resveratrol. Oxidative stress progressively disrupted the dynamic network, enabling ROS-responsive sustained release while simultaneously consuming reactive oxygen species through the cleavage of phenylboronate bonds. The responsive construct exhibited the prolonged intra-articular retention and consistently improved outcomes across molecular, cellular, and tissue levels, including macrophage repolarization, suppression of inflammatory mediators and matrix-degrading enzymes, preservation of cartilage matrix and attenuation of osteoarthritis progression in both chemically induced and surgically induced animal models [174]. Endogenous ROS levels, hydrogel activation and local resveratrol concentrations were not monitored simultaneously in vivo. As a result, although the study convincingly demonstrated therapeutic functionality of the responsive construct, it did not directly establish the complete mechanistic sequence linking pathological ROS accumulation to the local drug exposure.

**Figure 8:**
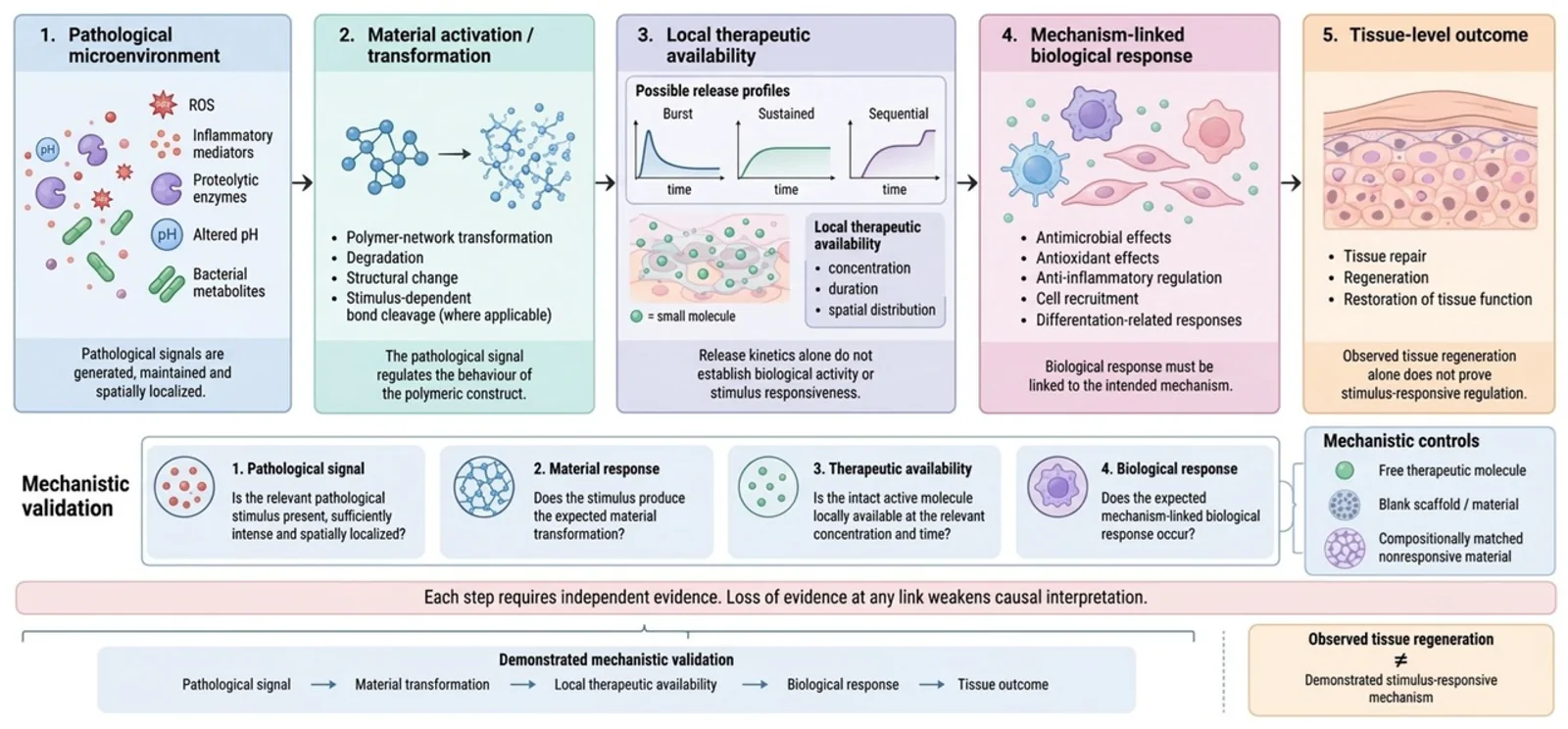
Mechanistic framework for validating stimulus-responsive function in polymeric biomaterials.

These studies illustrate complementary rather than equivalent levels of mechanistic validation. Joshi et al. experimentally demonstrated that disease severity regulates material activation; Liu et al. combined responsive material design with comprehensive evaluation of the downstream biological effects. Integrating these approaches remains an important challenge for the field. Future in vivo studies would benefit from the combination of quantitative assessment of the activating pathological signal, direct monitoring of the material transformation, measurement of local therapeutic availability and comprehensive biological characterization within the same experimental framework (Figure 8). Such designs would provide direct evidence that the stimulus-responsive regulation persists after implantation and that this regulation, instead of sustained drug retention alone, contributes to the observed therapeutic outcome.

### 6.7. Perspective on Preclinical Evaluation

Taken together, the preclinical evaluation of smart polymeric biomaterials is not simply a collection of independent characterization assays, biological experiments and animal studies. It represents a continuous process of mechanistic validation in which each experimental stage addresses specific uncertainty while providing evidence required for the next level of investigation. Physicochemical characterization establishes the integrity of the final construct, stimulus-responsive studies define the relationship between pathological cues and material activation, cellular models verify the preservation of the intended biological mechanism, and the in vivo experiments determine whether this mechanistic sequence remains functional within the complexity of living tissues. The loss of continuity at any stage weakens the causal interpretation, even when regenerative outcomes appear favorable.

From this perspective, the future development of smart polymeric materials will depend not only on the discovery of increasingly sophisticated responsive chemistries, but also on the evolution of preclinical evaluation strategies. Greater emphasis should be placed on quantitative mechanistic validation, the integration of advanced in vitro models with disease-relevant animal studies, and the experimental designs capable of linking pathological stimulus, material transformation, local therapeutic availability and biological response within a single framework. Ultimately, the principal objective of the preclinical evaluation is not to demonstrate that a material regenerates tissue, but to determine whether tissue regeneration occurs because the material preserves its intended stimulus-responsive mechanism after implantation.

From this perspective, stimulus responsiveness is better regarded not as an additional material property to be characterized, but as the central therapeutic mechanism that must be verified throughout preclinical development.

Accordingly, the most informative preclinical strategy is not necessarily the most complex one, but the one in which each model is selected because it resolves a defined biological uncertainty that cannot be adequately addressed by a simpler experimental system.

### 6.8. Scope and Limitations

Scope and limitations of approaches and materials development. Current development strategies for the incorporation of small molecules into polymeric systems extend from the direct entrapment and adjustment of the network density to affinity-based retention, covalent conjugation, compartmentalized carrier-in-matrix architectures, and stimulus-responsive or sequential delivery. This diversity provides substantial control over the molecular localization and exposure but also reveals a fundamental constraint: construct performance cannot be inferred from the polymer or cargo considered separately. Release emerges from the interplay between molecular size, charge and hydrophobicity, scaffold hydration and architecture, polymer–molecule interactions, degradation, and the conditions generated by the surrounding tissue. The relevant design unit is therefore the complete polymer–molecule system rather than the scaffold alone [145,175]. This interdependence is particularly restrictive for small molecules, which may diffuse through highly hydrated networks too readily to be retained by steric confinement alone. Electrostatic, hydrophobic and affinity interactions can reduce premature loss, while covalent attachment can markedly suppress burst release; however, stronger retention is not synonymous with more effective delivery. Increasing polymer concentration or crosslink density simultaneously alters the mesh size, swelling, mechanics and degradation, whereas high-affinity binding may reduce the freely diffusible fraction. Permanent conjugation is appropriate when surface presentation is intended, but less suitable for compounds whose activity requires release and cellular uptake. Incorporation into liposomes, micelles or polymeric particles can improve solubility and protection yet introduces additional transport barriers between the nominally incorporated dose and the amount that becomes available to cells. Such hierarchical systems exchange simpler release control for greater architectural and analytical complexity.

Architectural miniaturization further expands the design space. Hydrogel microspheres offer injectability, high surface area, interparticle transport pathways, and the capacity to separate cargoes into distinct compartments. Their function, however, depends strongly on particle size distribution and fabrication history. Microfluidic methods provide close control over the diameter and monodispersity but remain limited by throughput, equipment complexity and cost; emulsion and mechanical fragmentation methods are more scalable but generally provide poorer control over the particle uniformity and batch consistency. These differences are functionally important because particle size influences diffusion distance, surface area, cargo content and response kinetics. In addition, fabrication processes and materials can interact directly with the cargo; for example, polydimethylsiloxane microfluidic devices may absorb hydrophobic small molecules and alter their effective concentration. The manufacturing route is not a neutral step following the material design, but one of the determinants of composition and release behavior [176].

Temporal control presents a separate biological limitation. Sustained release is often treated as an intrinsic improvement, although tissue regeneration proceeds through partially overlapping phases that may require different, and sometimes opposing, pathway modulation. A molecule beneficial during the early inflammation or cell recruitment may become ineffective or detrimental during differentiation and remodeling. Sequential systems attempt to reproduce this biological sequence by exploiting differences in solubility, scaffold affinity, carrier degradation or stimulus sensitivity. Nevertheless, most reported constructs achieve only relative differences in release rate rather than clearly separated therapeutic time windows, leaving substantial overlap and premature leakage. The appropriate objective is thus not maximal release duration but delivery of an effective dose at the stage during which the targeted pathway is functionally relevant [175,177].

Mechanistic interpretation is likewise limited by the frequent conflation of diffusion, release, erosion and degradation. Loss of mass may reflect polymer dissolution, fragmentation, erosion, additive leaching or cargo release without demonstrating cleavage of the polymer backbone. Conversely, chemical degradation may occur before substantial mass loss or mechanical failure becomes apparent. A release profile can be described as degradation-controlled only when the changes in molecular flux are experimentally linked to chain cleavage or matrix erosion. Gravimetry and imaging are insufficient on their own and should be combined with chemical or molecular-weight analysis, while degradation products require separate safety assessment because the biocompatibility of the intact scaffold does not guarantee the safety of its breakdown products [148].

These constraints favor minimal sufficient complexity as a development principle. Additional carriers, compartments or responsive chemistries are justified only when they provide a measurable and mechanistically justified improvement in retention, molecular stability, temporal exposure, or biological specificity. Rational development requires quantitative characterization of polymer–molecule interactions, differentiation of release from material degradation, verification of responsiveness within tissue-relevant stimulus ranges, and incorporation of manufacturability from the outset. The most promising future systems are likely to be those with the largest number of nominally smart functions, but those in which the material transformation is reproducibly connected to an intact, biologically available dose within the required therapeutic window.

## 7. Clinical Trials

Traditional dosage forms, despite their effectiveness, have a number of properties that may reduce the efficacy of the treatment and lead to the search for new methods of drug delivery [178]. One of the most common problems is the low solubility of the active substances [179]. For example, paclitaxel has very low water solubility, which is why Cremophor EL is used to formulate it in its conventional dosage form. However, this excipient may cause hypersensitivity reactions and exacerbate the drug’s toxic effects [180,181].

A fast metabolism or chemical instability can also reduce the effectiveness of treatment, as the duration of drug action is shortened. For example, the use of camptothecin was limited by its low solubility, the instability of its active lactone form, and its toxicity. Under physiological conditions, the active form of camptothecin can rapidly convert to a less active form, which reduces the drug’s effectiveness [182,183].

Furthermore, the nonspecific distribution of medicines means that the active substance affects not only the site of the disease but also healthy tissue. Consequently, an increase in the dose may be required to achieve the desired effect. However, increasing the dose also increases the likelihood of side effects occurring [184]. This problem is particularly common with cytotoxic anti-tumor drugs, which damage not only tumor cells but also normal, rapidly dividing cells [182].

All of this leads to high toxicity and presents a therapeutic dilemma in which the use of medicine to treat a condition may place a significant burden on the body. In some cases, the adverse effects prove to be comparable to the expected therapeutic benefit, or even more significant for the patient [185]. Therefore, when developing new dosage forms, it is necessary to take into account not only the drug’s ability to treat the condition, but also its safety, duration of action and distribution within the body [186].

The introduction of polymer encapsulation as a delivery system may have positive effects. Controlled release, modified pharmacokinetics, molecular protection and localized delivery may enhance the drug’s efficacy [179,182]. This can prolong the duration of the action of the active substance through its sustained release, reduce the frequency of administration and lower the overall toxicity to the body by minimizing the impact on healthy tissues. Furthermore, a polymeric carrier can improve the solubility of poorly soluble substances and alter their distribution within the body [184].

An example of such a system is the Genexol-PM polymeric micelle, which contains paclitaxel. Its development was driven by the need to address the issue of paclitaxel’s low solubility and to move away from the use of Cremophor EL. In clinical trials, Genexol-PM enabled paclitaxel to be administered in an aqueous formulation and demonstrated efficacy comparable or superior to that of the traditional formulation [181]. Another example is the CRLX101 polymer system containing camptothecin, which is designed to enhance the stability of the active substance and ensure its gradual release within the body [187,188].

However, despite all the anticipated positive effects, there is a significant gap between the number of systems under development that show promising results in in vitro models and those that reach the clinical trial stage or are granted marketing authorization [189]. A quantitative analysis of nanoparticles, published in 2023, identified 486 clinical trials conducted between 2002 and 2021. However, only 8 percent of the trials were phase III trials, while the majority were phase I or phase II trials. Among the studies conducted between 2016 and 2021, polymeric nanoparticles accounted for around 7 percent [190]. It should be borne in mind, however, that such data relate to nanomedicine as a whole or to polymeric nanoparticles as a class, rather than solely to the small-molecule encapsulation systems. Therefore, despite the high proportion of polymer systems under development, only a small percentage successfully reach clinical practice, indicating the existence of a significant translational gap.

This discrepancy may be due to the fact that results obtained in cell cultures and animal models do not always reflect the behavior of the system within the human body. The efficacy of polymeric particles may be influenced by their size, charge, stability, interaction with plasma proteins, uptake by cells of the immune system, and the heterogeneity of the pathological tissue. Furthermore, even successful accumulation of particles in the diseased area does not always result in the release of drug in the required quantity and at the desired site [179,182].

In this section, we will examine the evolution of polymer encapsulation technology for small molecules from the perspective of clinical challenges. The main focus will be on the problems associated with the traditional dosage forms that the polymeric carriers have been used to address, the benefits achieved through their use, and why the results obtained in vitro models and at the preclinical stage do not always lead to the successful completion of the clinical trials.

### 7.1. The Evolution of Polymer Systems: From Depot Systems to Multifunctional Systems

Like any field of development, the evolution of polymer-based drug delivery systems has moved towards greater complexity and increased functionality. The first stage involved depot systems, which addressed the problem of frequent administration and fluctuations in drug concentration within the body. In such systems, the drug is gradually released from the polymer matrix, allowing for longer intervals between doses and maintaining the therapeutic effect for a longer period of time [189]. However, even for modern depot preparations, the initial rapid release of the active substance—or “burst release”—remains a significant problem. The rapid release of active molecules can lead to a reduction in exposure time, an increase in the rate of metabolism and elimination from the body, as well as the occurrence of local toxic effects due to a temporary increase in the concentration at the site of administration.

In 2003, the FDA approved Risperdal Consta [191]. This is an injectable form of risperidone encapsulated in microspheres based on a PLGA copolymer of lactic and glycolic acids. Risperidone is distributed within the polymer matrix, and its release occurs through drug diffusion and gradual degradation of the polymer. Consequently, the active substance is released over a prolonged period rather than being released into the body all at once [192]. This relatively simple system resolved the problem of fluctuations in drug concentration and extended the intervals between doses. However, a new challenge arose: controlling the initial release phase, preventing premature drug release and ensuring the reproducibility of microspheres [182,193].

The next stage in the development of technology addressed issues relating to the solubility and bioavailability of poorly soluble small molecules. The forms of these systems also changed: nanoparticles and polymeric micelles emerged, which could be used to produce aqueous formulations of hydrophobic drugs and to modify their pharmacokinetics.

Paclitaxel is an effective anticancer drug; however, its use in its traditional form is complicated by its very low solubility in water. Cremophor EL is therefore used to prepare a solution. However, in this form, the drug exhibited high toxicity and could cause hypersensitivity reactions. It was precisely the replacement of solvent with the polymeric micellar form Genexol-PM that made it possible to resolve one of the drug’s main pharmaceutical problems [180,194]. In clinical trials, Genexol-PM was compared with the conventional paclitaxel formulated with Cremophor EL. In a phase III trial, Genexol-PM demonstrated efficacy at least equivalent to that of the standard formulation, whilst the objective response rate was even higher: 39.1% for Genexol-PM versus 24.3% for the conventional paclitaxel. However, no statistically significant differences were observed in overall survival or progression-free survival [182]. Although this case demonstrates that the removal of an excipient can provide a compelling clinical rationale, the polymer matrix does not eliminate all the limitations of paclitaxel: the toxicity of the molecule itself, the need for intravenous administration, and the dependence of efficacy on tumor sensitivity remain. Furthermore, replacing Cremophor EL does not completely eliminate side effects such as neutropenia and peripheral neuropathy.

Further modification of micelles does not always lead to improved clinical outcomes. In a phase III trial of the polymeric micellar formulation of NK105 with paclitaxel, it was not possible to demonstrate the required non-inferiority in progression-free survival compared with standard paclitaxel [195]. Thus, unlike Genexol-PM, which demonstrated a benefit in terms of objective response rate, NK105 did not show a sufficient benefit in terms of the primary clinical endpoint, progression-free survival.

This example demonstrates that improving pharmacokinetics, increasing the circulation time of nanoparticles or reducing a specific type of toxicity does not always lead to an improvement in the most important clinical outcomes. Therefore, for a polymer-based system to progress to the later phases of clinical trials, it is necessary to demonstrate not only a change in the drug’s properties but also a real benefit for the patient [196].

The next milestone in this field is the use of active targeting in polymer systems. The surface of the particles is modified by attaching ligands, antibodies, peptides or other recognition fragments. It was thought that this would enable the drug to act selectively, thereby reducing its effective dose and the systemic burden on the body.

An example of such a system is BIND-014—a polymeric nanoparticle containing docetaxel, the surface of which is modified with a ligand for the prostate-specific membrane antigen (PSMA). This system was developed to enhance the delivery of docetaxel to PSMA-positive tumor cells. In a phase II clinical trial, BIND-014 demonstrated antitumor activity in patients with metastatic castration-resistant prostate cancer: the median radiographic progression-free survival was 9.9 months [197].

However, active targeting simultaneously complicated the formulation of the drug and increased the demands on quality control. It was necessary to monitor the size of the nanoparticles, the docetaxel content, the properties of the polymer matrix, the number of ligands on the surface, and the preservation of their ability to bind to PSMA. Furthermore, the presence of the ligand could affect the stability of the system, its interaction with plasma proteins, and its uptake by immune system cells.

Another problem was that the presence of the receptor did not guarantee the same level of efficacy in all patients. PSMA expression levels can vary between tumors and change over the course of the disease and the treatment. Furthermore, even when a particle binds to the receptor, this does not guarantee that it will effectively penetrate the tumor tissue and release the required amount of drug. Consequently, active recognition of the tumor receptor did not always result in a clinical benefit [191].

The next stage involved the development of modern, multifunctional systems. These combine several functions as follows: targeting, sensitivity to pH or enzymes, combined delivery of multiple drugs, and diagnostic imaging. Such systems are potentially more effective, but at the same time become more complex in terms of production, composition control, determination of the mechanism of action, and the regulatory approval [198].

An example of a multifunctional system at the preclinical stage is the pH- and ROS-sensitive PEG-PMT polymeric micelles containing docetaxel. This system was designed to respond to the acidic environment and elevated levels of reactive oxygen species characteristic of the tumor tissue. In preclinical experiments, the micelles released more than 85% of the drug under conditions mimicking the tumor microenvironment and inhibited tumor growth in the CT-26 model [199].

Multifunctionality increases the complexity of the system, raising the number of parameters that need to be taken into account and monitored. This leads to longer clinical trial durations and makes it more difficult to determine the pharmacokinetic and pharmacodynamic properties of the system. There is also the issue of production scalability: complex systems require more production stages. This complicates the management of quality control and safety assurance [196]. The system must have sufficient capabilities to address a specific clinical problem, whilst remaining reproducible, stable, scalable and understandable in terms of its mechanism of action.

### 7.2. Studies of Clinical Trial Data

Clinical translation of polymer-based delivery systems for small molecules has primarily focused on polymeric micelles, nanoparticles and polymer–drug conjugates. These systems have been investigated to improve aqueous solubility, reduce toxicity and modify the pharmacokinetics of anticancer drugs. Among the most frequently studied molecules are doxorubicin, epirubicin, cisplatin, oxaliplatin, SN-38, docetaxel and paclitaxel. However, despite many successful preclinical developments, only a limited number of polymer-based delivery systems have progressed to the early-phase clinical development, and even fewer have been approved for actual clinical use. Table 5 summarizes selected polymer-based delivery systems that have entered clinical trials, undergoing or having completed clinical trials.

The clinical examples presented in this section were identified through a targeted search of published literature data and clinical trial registries using combinations of terms related to polymer-based drug delivery, polymeric carriers, small-molecule therapeutics, and clinical development. The selection was intended to provide representative examples covering different polymer architectures, therapeutic agents, clinical development stages, and translational outcomes, rather than to constitute an exhaustive systematic review of all clinical trials involving polymer-based drug delivery systems. Particular attention was given to the examples illustrating common challenges in clinical translation, including limited efficacy, toxicity, pharmacokinetic limitations, and discontinuation of the development.

Targeted or multifunctional systems are still at the preclinical stage. Table 6 lists the targeted and multifunctional systems that are currently under development.

A separate area of development in polymer systems involves the combination of active targeting and controlled release. In such systems, a ligand that recognizes a tumor cell receptor is attached to the polymer particle, whilst the release of the drug is made sensitive to pH, enzymes, or reactive oxygen species.

For example, the folate-targeted dextran nanoparticles loaded with doxorubicin combined receptor-mediated recognition of tumor cells with the pH- and redox-dependent drug release [223]. Another example is cRGD-modified nanoparticles carrying doxorubicin, designed to interact with integrins on the tumor endothelium and tumor cells [224,225].

Most such developments remain at the preclinical stage, as adding more functions simultaneously complicates the system’s design, quality control, production scaling and interpretation of the mechanism of action [226].

### 7.3. Barriers for Clinical Transition

The clinical translation of polymer-based drug delivery systems can be limited by several interconnected barriers. These include insufficient clinical efficacy despite improved drug delivery, inadequate drug release or tissue and cellular penetration, differences between preclinical models and human physiology, manufacturing and reproducibility challenges, increased complexity of the multifunctional systems, and economic and regulatory constraints. The following examples illustrate these barriers and show why promising pharmacokinetic or formulation characteristics do not necessarily translate into improved clinical outcomes.

Improved drug distribution does not necessarily translate into improved clinical efficacy. An example of this is NC-6004, or Nanoplatin, a polymeric cisplatin delivery system based on PEG-poly-L-glutamic acid. It was hypothesized that such a system would alter the distribution of cisplatin, reduce its toxicity and increase the concentration of the drug in the tumor tissue [215]. However, the presence of a polymeric carrier did not in itself guarantee improved efficacy compared with the standard therapy. In clinical trials, the system was studied primarily in combination with other treatments, and its further development was limited by the lack of a convincing advantage in terms of key clinical outcomes [227].

A second barrier is insufficient drug release or cellular penetration despite successful drug targeting or redistribution. The presence of targeting does not necessarily lead to improved treatment outcomes. In addition to the direct delivery of the drug to the tumor, it must be released in an effective concentration and penetrate into the cell.

Another barrier is the absence of a sufficiently clear clinical advantage over the existing therapy. SP1049C is a polymeric micellar formulation of doxorubicin intended for the treatment of inoperable or metastatic adenocarcinoma of the esophagus and the esophagogastric junction. The system comprised Pluronic L61 and F127 block copolymers, which were intended to improve the distribution of doxorubicin and alter its interaction with tumor cells. Although the formulation demonstrated antitumor activity and reached phase II clinical trials, the available clinical results did not establish a sufficient benefit to support the widespread regulatory adoption [215].

A further barrier is manufacturing complexity and the associated cost of production and quality control. Even if the new system is as effective as the original therapy but entails higher production and quality control costs, its clinical application may be limited. In this case, the barriers are commercial and manufacturing risks rather than biological ones.

NC-6300, a pH-sensitive polymeric micelle containing epirubicin, illustrates the challenge of manufacturing reproducibility in complex polymeric systems. In this system, the drug is bound to the polymer matrix via a hydrazone bond, which must be relatively stable in the bloodstream and break down in the acidic environment of the tumor or in intracellular compartments. Therefore, the efficacy of the system depends not only on epirubicin content and particle size, but also on the stability of the chemical bond, the rate of its breakdown, the release profile and properties of the polymeric micelle [201].

Such a large number of parameters may require changes to the polymer composition in order to simplify the manufacture of the formulation and maintain the stability of the system. As a result, we end up with a system which, in clinical trials, may differ significantly from the preclinical findings and require additional time to optimize the composition and the manufacturing process [215].

Another major barrier is the poor predictability of preclinical efficacy in humans. An example of a system that illustrates the gap between preclinical and clinical practice is the pH- and ROS-responsive PEG-PPMT micelles containing docetaxel [221]. This system was developed to respond to the characteristics of the tumor microenvironment—a lower pH and elevated levels of the reactive oxygen species. In preclinical models, the micelles facilitated controlled release of docetaxel and inhibited tumor growth [220].

Promising results in cell cultures and animal models do not necessarily predict clinical efficacy in humans. Within a patient’s body, the concentration of the reactive oxygen species, the acidity of tumor tissue, the rate of polymer degradation and the accessibility of the particles to tumor cells may differ significantly from the experimental conditions. PEG–PPMT exemplifies the typical gap between the promising preclinical characteristics and a lack of clinical evidence of efficacy.

The increasing formulation complexity represents another important barrier to clinical translation. This is particularly relevant to systems that combine active targeting with several controlled-release mechanisms. For example, the folate-targeted dextran nanoparticles loaded with doxorubicin simultaneously utilize binding to folate receptors, pH sensitivity, and redox-dependent drug release. It was hypothesized that the folate ligand would enable the recognition of tumor cells, whilst the characteristics of the intracellular environment would facilitate the release of doxorubicin [222].

Such a system clearly illustrates the advantage of a multifunctional approach, but at the same time highlights its main drawback. For a single dosage form, it is necessary to control the particle size, the composition of the dextran matrix, the degree of folate binding, the stability of the preparation, its sensitivity to pH and reducing agents, as well as the doxorubicin release profile [182]. Thus, each additional functional component introduces additional critical quality attributes that must be controlled during the formulation, scale-up, and quality assessment. Increasing the number of functions also increases the possibility of interactions between the formulation parameters, potentially complicating reproducibility and regulatory evaluation. Consequently, the increasing formulation complexity can hinder reproducible manufacturing, scale-up, and regulatory evaluation, potentially limiting the progression of the multifunctional nanoparticles into clinical development [228].

The examples given show that there can be various reasons for early termination of clinical trials. The transition from in vitro models and in vivo animal studies to scaling up and transferring the findings to the human body can yield completely different results [229]. Systems that focus on concentrations of specific compounds or on receptor binding may encounter difficulties due to individual patient characteristics, as gene expression or the receptor sensitivity can vary considerably between patients [230,231]. Solving the problem of drug redistribution does not always result in its release at a sufficient concentration, which also negates the results achieved. The increasing complexity of these systems not only leads to an expansion of the parameters used to assess the drug’s effect on the body and complicates the conduct of pharmacokinetic and pharmacodynamic studies, but also requires a meticulous approach to manufacturing, the introduction of additional safety protocols and product quality assessment [232]. If the new system produces results similar to those of the conventional therapy, its roll-out may be limited due to production barriers and the financial costs of manufacturing [233].

### 7.4. Design Principles for Successful Clinical Translation

To bridge the gap between preclinical and clinical research on polymer systems, it is necessary to identify the key areas that can increase the likelihood of a successful transition from development to clinical application. As discussed above, increasing the complexity of the design and number of functions does not always lead to improved efficacy. On the contrary, multifunctional systems require more complex manufacturing, quality control and evaluation of the mechanism of action. Therefore, one of the important principles is to avoid unnecessary complexity and focus the system on a specific clinical problem, such as increasing solubility, prolonging the duration of action or reducing the toxicity of drug.

It is also necessary to assess in advance the system’s scalability and to take into account the technological changes that will be required for industrial production. When scaling up from the laboratory to industrial scale, particle size, encapsulation efficiency, surface properties and the drug release profile may change. The stability of the polymer matrix and the reproducibility of the characteristics across individual batches are key prerequisites for the successful conduct of clinical trials and the subsequent registration of the medicinal product. Therefore, manufacturing reproducibility and scalability should be considered from the early stages of formulation development rather than only during later clinical development.

An important area of research is the determination of key pharmacokinetic and pharmacodynamic parameters. It is necessary to assess not only the concentration of the total drug molecule in the blood, but also its rate of release, distribution in tissues, the duration of the active form, and the relationship between these parameters and the therapeutic effect. For the targeted and stimulus-responsive systems, it is necessary to use more realistic cellular and animal models that take into account tumor heterogeneity, receptor expression, and the interaction of particles with plasma proteins and cells of the immune system. This may help to reduce the gap between the pre-clinical results and the actual behavior of the system in patients.

Finally, it is necessary to compare not only the efficacy but also the cost of the new system with that of the standard therapy. If clinical outcomes are comparable, a polymeric formulation that is more expensive and complex to manufacture may prove to be economically unviable. Accordingly, another important principle is that the new formulation should provide a demonstrable clinical advantage over existing therapy that justifies its additional complexity and cost. Therefore, systems with the greatest chance of successful translation are those that address a specific clinical problem, remain stable, can be manufactured reproducibly and offer a proven advantage over existing treatments.

## 8. Conclusions

This systematic review compiles the current approaches for the development of versatile delivery systems for small drug molecules. In addition, the review identifies the key challenges that need to be addressed along the translational pathway from fundamental and applied research to clinical application. Simplifying the design of multifunctional systems to facilitate their manufacturing, integrating machine learning algorithms to predict their properties and optimize system composition, and developing standardized protocols for preclinical evaluation using animal models represent key promising directions for future development in this area. The knowledge covered in this review may be useful for researchers in planning future studies and selecting appropriate methods and approaches for the development and characterization of polymer-based delivery systems.

## Figures and Tables

**Table 1:** Adapted from [73] with permission from John Wiley and Sons, 2023.

Model	Equation	Key Parameters	Specific Notes	Application
Zero-order	$Q_{t}=Q_{0}+K_{0}\cdot t$	*Q*_t_: amount of drug dissolved *Q*_0_: starting amount of drug in solution (usually *Q*_o_ = 0) *K*_0_: Zero-order release constant (g m^−3^s^−1^) *t*: time of drug release	For pharmaceutical dosage forms with a slow drug release rate and without disaggregation, the zero-order model is applicable. This model assumes that the dosage area remains constant and that the equilibrium is not reached. The impact of time on the release rate constantly changes with changes in concentration.	Zero-order drug delivery systems offer the potential to precisely tune release kinetics to extend the drug concentrations within the therapeutic window. As a result, these systems can recover the drug efficacy and reduce the side effects. They have the potential to dominate the issues facing the immediate release. Zero-order drug delivery systems have been bounded to wide and costly devices that are not attractive for the most use cases because of their high value, finite time, and inconvenient application, which may need the surgery.
First-order	$\ln\left(Q_{t}\right)=\ln\left(Q_{0}\right)-K_{F}\cdot t$	*K*_F_: first-order release constant (s^−1^) *Q*_t_: drug released *Q*_0_: initial drug concentration *t*: time of drug release	With the release data plotted against time and a straight line with a slope of *K*, percentage of remaining drug is represented. Utilization for the dissolution rate is proportional to the difference between the instantaneous concentration and the saturation solubility. The drug concentration has a direct relationship with its release rate.	By changing the model parameters, this straightforward model may be adjusted to fit a variety of nanocarriers. This model perfectly describes the release kinetics of water-soluble agents in porous matrices and ionizable oil or water-soluble materials from W/O/W emulsions. First-order systems by releasing drugs at a constant rate, thereby holding the drug concentrations for an extra time.
Higuchi	$Q_{t}=K_{H}\cdot t^{\frac{1}{2}}$	*Q*_t_: drug released *K*_H_: release rate constant in the Higuchi model *t*: time of drug release	The Higuchi model makes the following assumptions: (1) the drug’s final concentration in the matrix is considerably higher than its solubility; (2) drug diffusion is one-dimensional, (3) the size of the dosage form is smaller than the thickness of the information gathering phase, (4) the swelling and dissolving of the matrix could be ignored, (5) the drug diffusion coefficient is steady, and (6) ideal sink situations are always achieved in the release surrounding.	Usage with nanoparticles in the system and a concentration higher than the solubility of the active component, active substances are continuously and one-dimensionally released from the matrix. The Higuchi model is not too difficult to use, even with the complex public transit system. By using this kind of model, it is simpler to comprehend how water-soluble pharmaceuticals are released from the managed drug delivery devices such as transdermal patches, matrix capsules, and encapsulated materials with low solubility that are encapsulated in solid or semisolid matrices. Determining the mechanisms by which various matrix systems release medicines that are lipid- and water-soluble.
Korsmeyer–Peppas	$\frac{Q_{t}}{Q_{\infty }}=K_{KP}\cdot t^{n}$	*Q*_t_/*Q*_∞_: fraction of drug release at a given time *K*_KP_: release constant incorporating structural and geometric dosage form characteristics	This model is used for the description of drug release from polymeric systems considering non-Fickian mechanisms. The model is useful when the release mechanism is unknown or when more than one type of drug release phenomenon is involved. This model is applicable only for the first 60% of the release curve [74]	Active ingredients that release from a polymeric system. Concurrently a few release mechanisms are explained, such as the diffusion of water into the matrix, matrix swelling, and matrix dissolution. Data gathered from the release profile of various formulations of microcapsules and microspheres can be linearized with the help of this type of model. The Fickian and non-Fickian drug release patterns are described by this model. When the release mechanism is unknown or more than one release mechanism is employed, this model is applicable.
Hixson–Crowell	$Q_{0}^{\frac{1}{3}}-Q_{t}^{\frac{1}{3}}=K_{s}\cdot t$	*Q*_t_: cumulative amount of drug release *Q*_0_: initial drug concentration K_S_: Hixson–Crowell release rate constant *t*: time of drug release	K_S_ is a release constant. It applies to systems in which the release rate is limited by the drug particles’ dissolution rate instead of diffusion through the polymeric matrix.	The release kinetics of planned shapes in which dissolution occurs in planes parallel to the bioactive agent surface area is best described. It is applicable to the tablets with a correspondingly decreasing size; dissolution occurs in planes parallel to the drug surface. The particles are spherical, dissolution proceeds normally to the solute particle surface, and agitation is homogeneous over the exposed surface. This model has been applied to describe the release of drugs through a non-disintegrating isotropic device.
Weibull	$\frac{Q_{t}}{Q_{\infty }}=1-e^{-{\alpha \cdot t}^{\beta }}$	*Q*_t_: drug released Q_∞_: amount of substance released upon complete “depletion” of the polymer matrix (capsule); *α* = scale parameter, defines the timescale of the process *β* = curve shape factor [74]	Providing a physical link between its parameters and the geometry of the system. Both immediate and prolonged drug release patterns adopt this empirical model. Release curve: *β* = 1: exponential curve *β* > 1: sigmoidal (S-shaped) curve *β* < 1: parabolic curve	This model compares fast and slow release patterns of the active components in the drug delivery systems. This model is suitable for almost all types of release cases. One element influencing total drug release is the effective surface area, which is solely dependent on the mass.

**Table 2:** Representative critical process parameters and resulting quality attributes of the main fabrication platforms.

Fabrication Platform	Representative Critical Process Parameters	Main Resulting Critical Quality Attributes
Hydrogels and in situ-forming systems	Polymer concentration and molecular weight; degree of functionalization; crosslinker stoichiometry; initiator concentration; light dose; gelation temperature and time	Degree of conversion; gel fraction; swelling and mesh size; gelation time; injectability; mechanical properties; residual reagents; cargo integrity and uniformity
Electrospinning	Polymer–solvent system; solution viscosity and conductivity; cargo solubility; flow rate; applied voltage; needle–collector distance; temperature and humidity	Fiber diameter and defects; porosity and alignment; residual solvent; cargo solid state; surface enrichment; loading uniformity; mechanical properties
Freeze-drying and phase-separation methods	Polymer and solvent concentration; cooling rate; temperature gradient; sample dimensions; phase-separation temperature; primary and secondary drying conditions	Pore size and anisotropy; pore interconnectivity; residual solvent and water; cargo concentration gradients; crystallization or phase separation; mechanical properties
Porogen leaching, gas foaming, and emulsion templating	Porogen size and content; leaching conditions; gas pressure and depressurization rate; emulsion composition; surfactant and initiator concentration	Total and open porosity; pore interconnectivity; residual porogen, surfactant, or monomer; cargo loss; dimensional stability; mechanical strength
Additive manufacturing	Ink or melt rheology; printing temperature; extrusion pressure; nozzle diameter; printing speed; layer height; light dose; post-curing and washing	Dimensional fidelity; filament continuity; interlayer adhesion; regional cargo content; cargo degradation; residual monomers or photoinitiator; mechanical anisotropy
Surface coating and layer-by-layer assembly	Surface pretreatment; polymer concentration; pH and ionic strength; deposition time; number and order of layers; washing; crosslinking	Coating thickness and coverage; surface cargo content; coating stability; delamination; pore blockage; surface chemistry; batch-to-batch uniformity

**Table 3:** Main post-processing risks and recommended controls.

Stage	Main Risks for the Polymer	Main Risks for the Small Molecule	Minimum Controls
Washing and solvent removal	Swelling, dissolution, loss of soluble fractions, incomplete removal of reagents	Loss of loosely retained cargo; redistribution	Mass balance; analysis of wash solutions; residual solvent/reagent testing
Drying and lyophilization	Changes in pores, dimensions, crystallinity, and mechanics	Migration, aggregation, amorphous–crystalline transition	Morphology; water content; actual loading; DSC/XRD; spatial mapping
Sterilization	Chain scission, crosslinking, oxidation, deformation, delamination	Degradation, premature cleavage or release, potency loss	Polymer molecular weight; mechanics; HPLC/LC–MS; loading; functional response
Packaging and storage	Hydrolysis, oxidation, water uptake, interface changes	Crystallization, phase separation, degradation, sorption to packaging	Real-time and accelerated stability; degradation products; content uniformity; package compatibility
Aseptic manufacturing	Changes caused by separate sterilization of components; contamination during assembly	Loss during sterile filtration; adsorption to filters or equipment; instability during sterile holding	Filter compatibility and recovery; component sterility; environmental monitoring; aseptic process validation; final sterility testing

**Table 4:** Comparative assessment of small-molecule incorporation strategies.

Strategy	Most Suitable Cargo Properties	Compatible Material Platforms	Main Determinant of Attainable Loading and Distribution	Release or Smart-Response Potential	Main Advantages	Principal Limitations and Manufacturing Considerations
Direct incorporation during fabrication [79,80,82,84,85,86,87]	Compounds stable in the selected solvent, temperature, crosslinking, and printing conditions and compatible with the polymer phase	Hydrogels, fibers, porous scaffolds, films, membranes, and printed constructs	Solubility and miscibility in the precursor; partitioning during solidification; loss during washing or solvent removal	Mainly passive diffusion-, swelling-, or degradation-controlled release; responsiveness may be introduced through the matrix	Simple formulation; few processing stages; bulk incorporation	Exposure to solvents, heat, radicals, light, or shear; crystallization and surface enrichment; effects on rheology, crosslinking, pore structure, and mechanics
Affinity-based incorporation [88,89,90,91,92,93]	Charged, hydrophobic, aromatic, or host–guest-compatible compounds capable of reversible binding	Functionalized hydrogels, ionic polymers, cyclodextrin-containing networks, supramolecular matrices, and coatings	Number, accessibility, and association strength of the binding sites; competition from ions, proteins, and metabolites	Passive affinity-controlled release or stimulus-dependent release when binding changes with the environment	Improved retention without permanent modification of the molecule; tunable binding strength	Incomplete release; limited freely available fraction; sensitivity to pH, ionic strength, and biological competitors; need for binding controls
Secondary-carrier-assisted incorporation [82,94,95,96,97]	Poorly soluble, rapidly diffusing, chemically unstable, or multi-agent cargo	Hydrogels, porous scaffolds, fibers, membranes, coatings, and printed constructs	Carrier drug loading, carrier fraction in the matrix, carrier distribution, and loss during final fabrication	Multistage, sequential, or carrier-triggered release	Cargo-specific microenvironment; molecular protection; separation of cargo loading from scaffold fabrication	Multistep processing; carrier aggregation or sedimentation; changes in rheology and mechanics; separate evaluation of carrier persistence and degradation
Post-fabrication loading [82,83,98,99,100]	Compounds that cannot tolerate the main scaffold-forming process but can diffuse into or partition into a preformed material	Porous scaffolds, fibers, hydrogels, membranes, printed constructs, and implantable depots	Accessible pore volume, wettability, incubation time, molecular diffusion, and polymer–drug partitioning	Mainly passive release; affinity or responsive coatings can be added after loading	Avoids exposure to the main fabrication conditions; modular adjustment of the dose or the cargo	Surface-enriched distribution; limited penetration in large constructs; cargo loss during handling and drying; burst release
Surface deposition, coatings, and layer-by-layer assembly [101,102,103,104]	Compounds intended for interface-localized action or compatible with a coating component	Films, membranes, fibers, porous scaffolds, implants, and printed constructs	Accessible surface area, coating thickness, layer number, and internal-surface coverage	Passive, staged, directional, or stimulus-dependent release from the surface layer	Strong spatial localization; limited modification of the bulk material; control of layer composition and the order	Limited reservoir volume; pore blockage; incomplete internal coverage; delamination; repeated deposition and washing steps
Non-cleavable covalent tethering [105,106]	Molecules that retain activity when immobilized and act through extracellular or surface-accessible targets	Functionalized surfaces, coatings, fibers, membranes, and hydrogel networks	Density and accessibility of reactive groups; conjugation efficiency; linker length and molecular orientation	Contact-dependent presentation without release of free cargo	Strong localization; minimal premature loss	Not suitable for molecules requiring cellular uptake; possible loss of activity; steric crowding; residual coupling reagents; should not be described as conventional delivery
Cleavable polymer–drug conjugation [106,107,108,109,111]	Molecules containing a modifiable functional group that can be released as an active parent compound or characterized derivative	Functionalized hydrogels, polymer chains, fibers, coatings, surfaces, and carrier–matrix systems	Degree of conjugation, linker stability, accessibility of the cleavage site, and preservation during fabrication and storage	Time-dependent or pH-, enzyme-, ROS-, redox-, or light-triggered release	Strong retention before activation; direct chemical connection between the trigger and release mechanism	Complex synthesis and purification; possible modification of the pharmacophore; incomplete cleavage; uncertain activity or toxicity of the linker-containing products

**Table 5:** Undergoing and completed clinical trials of polymer-based delivery systems for small molecules.

System Name	Condition/Indication	Small Molecule	Polymer Matrix	Clinical Trial Phase	Clinical Development Status/Outcome	Primary Translational Barrier	Reference
NK-911	Solid tumors	Doxorubicin	PEG-b-polyaspartic acid	I/II	Outcome unavailable	Not reported/established	[200]
NC-6300 K-912	Soft tissue sarcomas, solid tumors	Epirubicin	PEG-b-polyaspartic acid with a pH-sensitive hydrazone bond	I/II	Outcome unavailable	Not reported/established	[201,202]
NC-4016	Common solid tumors, lymphomas	Oxaliplatin	PEG-b-poly-L-glutamic acid	I	Clinically promising but not further developed	Clinically promising but not further developed	[203] [NCT03168035]
NK-012	Small-cell lung cancer, breast cancer, solid tumors	SN-38	PEG-b-poly-L-glutamic acid	II	Clinically promising but not further developed	Not reported/established Safety/toxicity Efficacy	[204] [NCT00951613]
NC-6004/Nanoplatin	Pancreatic cancer and other solid tumors	gemcitabine	PEG-b-poly-L-glutamic acid	II/III	Completed; no NDA submitted	Efficacy Safety/toxicity	[205]
SP1049C	Advanced gastric adenocarcinoma	Doxorubicin	A mixture of Pluronic L61 and F127	II/III	Clinically promising but not further developed	Not reported/established	[205]
Nanoxel^®^-M *	Breast cancer, non-small cell lung cancer, other tumors	Docetaxel	mPEG-b-poly(D,L-lactic acid)	I/III	Completed approved/marketed in South Korea, further clinical evaluation ongoing or indication-dependent	-	[206]
Nanoxel-M/SYP-0704A * A polymeric micelle of docetaxel	Squamous cell carcinoma of the esophagus	Docetaxel	An amphiphilic block copolymer; the exact composition depends on the protocol	II	Outcome unavailable	Not reported/established	[NCT03585673]
Nanoxel^®^M A polymeric micelle of docetaxel	BCG-refractory non-muscle-invasive bladder cancer	Docetaxel	Polymeric micellar system	III	Terminated	Not reported/established	[NCT02982395]
A polymeric micelle of docetaxel	Common solid tumors	Docetaxel	Polymeric micellar system	II	Not yet recruiting	Not applicable	[NCT05254665]
PM-paclitaxel Squamous cell carcinoma of the head and neck Paclitaxel A polymeric micelle not belonging to the Genexol-PM or NK105 groups				II	Not yet recruiting	-	[NCT06366945]
NC-6300 in combination with Pembrolyzumab				III	Completed	Favorable efficacy compared with conventional paclitaxel	[NCT02667743]
Chitosan-coated miconazole nanoparticles				Not Applicable	Completed	-	[NCT02064829]
The poloxamer formulation of raltegravir				II	Terminated	Poor patient accrual	[NCT03008512]
NK-911-like doxorubicin micelles	Solid tumors	Doxorubicin	PEG-polyaspartate	II	Discontinued/canceled	Not reported/established	[200,207]
NC-6300 polymeric micelle of epirubicin	Advanced solid tumors; advanced, metastatic or unresectable soft-tissue sarcoma	Epirubicin	Polymeric micelle based on PEG–poly(aspartic acid	I/II	Active/ongoing	-	[201] [NCT03168061]
Chitosan-coated miconazole nanoparticles	Oropharyngeal candidiasis	Miconazole	Chitosan	I	Completed	Failed to demonstrate superior efficacy	[208] [NCT06072716]
Poloxamer film-coated raltegravir tablet	HIV infection	Raltegravir	A poloxamer system	I/II	Completed	-	[NCT00485264]
CT-2103/Xyotax	Non-small cell lung cancer, ovarian cancer	Paclitaxel	Polyglutamic acid	III	Discontinued/not approved	Failed to demonstrate superior efficacy; EU application withdrawn	[209]
PNU-166945	Solid tumors	Paclitaxel	A paclitaxel conjugate with an HPMA copolymer	I/II	Discontinued prematurely	Severe neurotoxicity	[209,210]
PNU-166148	Solid tumors	Camptothecin	A conjugate of camptothecin and an HPMA copolymer	I/II	Discontinued/withdrawn from clinical development	Subsequent pharmacodynamic studies and dose-limiting bladder toxicity	[211,212]
AP5280	Common solid tumors	Cisplatin	HPMA copolymer platinum conjugate	I	Development discontinued/deferred	Development was shifted to the next-generation AP5346 (ProLindac); limited clinical efficacy was also reported	[213]
ProLindac/AP5346	Recurrent ovarian cancer and other solid tumors	Oxaliplatin	HPMA copolymer with a pH-sensitive bond	I/II	Discontinued	Not reported/established	[214] [2010-020030-25]

* The study data relate to a broad drug platform—docetaxel polymer micelles—but the cited sources do not provide sufficient information to confirm that it is the same commercial product.

**Table 6:** Targeted and multifunctional delivery systems in preclinical and clinical trials.

Name	Small Molecule	Targeting	Additional Feature	Status	References
BIND-014	Docetaxel	PSMA	Controlled release, prolonged circulation	Phase II	[197,215]
Dextran nanoparticles	Doxorubicin	Folate receptor	pH/redox sensitivity	Preclinical	[216]
PEG-b-PTMBPEC	Doxorubicin	cRGD/αvβ3	pH sensitivity	Preclinical	[217,218]
F–P-DOX	Doxorubicin	Folate receptor	pH sensitivity, MRI	Preclinical	[219]
PEG-PPMT	Doxorubicin	None or passive accumulation	pH/ROS sensitivity	Preclinical	[220,221]
Folate nanomicelles	Doxorubicin + paclitaxel	Folate receptor	pH/redox sensitivity, co-delivery	Preclinical	[222]

## Data Availability

No new data were created or analyzed in this study. Data sharing is not applicable to this article.

## Abbreviations

The following abbreviations are used in this manuscript:

5-FU	5-fluorouracil
AFM	atomic force microscopy
AI	artificial intelligence
BC	bacterial cellulose
BSANP	bovine serum albumin nanoparticles
CARS	coherent anti-Stokes Raman scattering
CNT	carbon nanotubes
cRGD	cyclic arginine-glycine-aspartic acid (cyclic RGD peptide)
DMA	dynamic mechanical analysis
DRIFT	diffuse reflectance infrared Fourier transform
DSC	differential scanning calorimetry
ECM	extracellular matrix
FDA	Food and Drug Administration
FTIR	Fourier-transform infrared spectroscopy
GelMA	gelatin methacryloyl
HEMA	2-hydroxyethylmethacrylate
HPMA	N-(2-hydroxypropyl)methacrylamide
HPLC	high-performance liquid chromatography
LC-MS	liquid chromatography-mass spectrometry
LCST	lower critical solution temperature
micro-CT	micro-computed tomography
ML	machine learning
MRI	magnetic resonance imaging
NMR	nuclear magnetic resonance
PCL	polycaprolactone
PEG	polyethylene glycol
PGA	polyglycolic acid
PLA	polylactic acid
PLCL	poly(L-lactide-co-caprolactone)
PLDLLA	poly(L-lactide-co-D,L-lactide)
PLGA	poly(lactic-co-glycolic acid)
PNIPAAm	poly(N-isopropylacrylamide)
PSMA	prostate-specific membrane antigen
ROS	reactive oxygen species
SEM	scanning electron microscopy
SF	silk fibroin
TEM	transmission electron microscopy
TGA	thermogravimetric analysis
TPP	two-photon polymerization
UV-Vis	ultraviolet-visible spectroscopy
VP	vinylpyrrolidone
XPS	X-ray photoelectron spectroscopy
